# SRF3 couples cellular iron homeostasis and defense responses through ubiquitin-dependent nano-organization

**DOI:** 10.1126/sciadv.aeh4671

**Published:** 2026-10-14

**Authors:** Matthieu Pierre Platre, Lukas Brent, Matias F. Gleason, Preyanka Mehta, Ling Zhang, Laia Armengot, Julie Neveu, Olivier Kaakeh, Min Cao, Laura M. Martins, Julie A. Law, Santosh B. Satbhai, Nuria S. Coll, Marc Valls, Grégory Vert, Wolfgang Busch

**Affiliations:** ^1^ Salk Institute for Biological Studies, Plant Molecular and Cellular Biology Laboratory, 10010 N Torrey Pines Rd, La Jolla, CA 92037, USA.; ^2^ IPSiM, Univ Montpellier, CNRS, INRAE, Institut Agro, 34060, Montpellier, France.; ^3^ Centre for Research in Agricultural Genomics (CRAG), CSIC-IRTA-UAB-UB, Campus UAB, Bellaterra, Catalonia, Spain.; ^4^ Departament de Bioquímica I Biologia molecular, Universitat Autònoma de Barcelona, Bellaterra, 08193 Barcelona, Catalonia, Spain.; ^5^ Plant Science Research Laboratory (LRSV), UMR5546 CNRS/University of Toulouse/Toulouse-INP, Toulouse, France.; ^6^ Consejo Superior de Investigaciones Científicas (CSIC), Barcelona 08001, Spain.; ^7^ Department of Genetics, Microbiology and Statistics, Universitat de Barcelona, Av. Diagonal 643, 08028 Barcelona, Catalonia, Spain.

## Abstract

Iron homeostasis is a central component of host defense responses. In mammals, iron availability is rapidly adjusted through receptor mediated pathways, whereas it remains unclear how plants regulate iron homeostasis during the earliest stages of immune activation. Here, we identify a rapid iron regulatory mechanism centered on the plasma membrane receptor kinase STRUBBELIG RECEPTOR FAMILY 3 (SRF3), which integrates bacterial immune signaling with ferrous iron homeostasis. Perception of flagellin triggers a burst of reactive oxygen species that induces a rapid and transient depletion of the labile ferrous iron pool. This state promotes SRF3 ubiquitination and clathrin-dependent endocytosis, a process required to restore intracellular ferrous iron levels while dampening immune responses. Using super resolution microscopy, we show that this functional integration relies on ubiquitin dependent nano organization of SRF3 at the plasma membrane, enabling distinct signaling outputs from a single receptor. Thereby, SRF3 modulates both immune signaling and the proliferation of commensal and pathogenic bacteria. Together, our findings uncover a fast-acting iron homeostasis pathway in plants that dynamically couples nutrient availability with immunity, thereby shaping host microbe interactions at the onset of infection.

## INTRODUCTION

Iron is a key nutrient essential for cellular functions. It plays critical roles not only in controlling cellular homeostasis through ROS regulation and serves as a cofactor for approximately one-third of the proteome (*1*). However, in excess, iron triggers the generation of cell damaging reactive oxygen species (ROS) via the Fenton and the Haber-Weiss reactions (*2*, *3*). Consequently, iron levels are tightly regulated as iron deficiency or iron excess reduces overall organismal fitness. Therefore, a sophisticated iron homeostasis network monitoring and balancing cellular iron levels is fundamental to ensuring cellular function (*1*).

Upon microbe-host interaction, iron is a critical determinant for the infection outcome. Invading microbes scavenge iron from the host to fuel their virulence and hosts restrict iron to limit pathogen proliferation. This iron limitation, known as nutritional immunity, is one of the first lines of defense and concerns primarily the ferrous form of iron [Fe^2+^, (*4*, *5*)]. However, as the host restricts iron availability, it must still retain sufficient iron amounts to sustain its own cellular functions and immune responses. Therefore, the host needs to strike a delicate balance during microbial invasion, limiting iron to impede bacterial growth while ensuring sufficient levels for its own essential cellular processes and overall survival (*4*). In mammals, the finely tuned sensory mechanism that interconnects immunity with iron sensing and homeostasis responses has been described: In response to inflammatory intracellular signals, systemic iron sequestration occurs through the interaction of ferroportin with the hormonal peptide hepcidin (*4*). Importantly, the decrease in intracellular iron availability triggers a fast-acting compensatory mechanism mediated by the clathrin-dependent endocytosis of the transferrin receptor (TfR), which releases ferrous iron (Fe^2+^) intracellularly (*6*, *7*). A negative feedback loop mechanism fine tunes the iron uptake, notably, by promoting lysosomal targeting of ubiquitinated TfR (*8*). This regulatory network plays a pivotal role in nutritional immunity, establishing an iron-restricted environment that impedes the growth of invading pathogens while ensuring sufficient iron levels for vital cellular functions (*4*). Much like epithelial tissues in animals, plant roots are constantly in contact with bacteria in soils, triggering host nutritional immunity responses (*9*). Yet, land plant genomes lack the TfR to adjust cellular iron uptake virtually instantly upon infection (*10*). In non-gramineous plants, the iron transporter IRON-REGULATED TRANSPORTER 1 (IRT1) requires 12–24 hours to be induced to high levels and can be repressed upon perception of pathogen-associated molecular patterns [PAMPs, (*11*–*14*)]. Similarly, gramineous plants rely on phytosiderophore-mediated iron uptake, which also operates on longer time scales (*15*). It is therefore unclear how plant cells cope with a rapid decrease in Fe^2+^ upon pathogen perception while still meeting their cellular iron demands for survival.

Recently, a fast-acting regulatory mechanism for responses to iron and immune cues has been identified (*16*). The receptor kinase STRUBBELIG RECEPTOR FAMILY 3 (SRF3) coordinates immune and iron homeostasis responses within the first hours of immune challenges in roots (*16*). Here, we explored the molecular mechanism by which SRF3 coordinates iron homeostasis upon bacterial perception. We found that upon recognition of flg22 by its cognate plasma membrane-associated receptor FLAGELLIN SENSING 2 (FLS2), the ROS burst produced by RESPIRATORY BURST OXIDASE HOMOLOGUE (RBOH) causes a transient decrease in ferrous iron. The decrease in ferrous iron facilitates SRF3 ubiquitination and subsequent plasma membrane removal through clathrin-dependent endocytosis. The resulting SRF3 internalization is required to replenish intracellular ferrous iron by a yet unknown mechanism. The non-ubiquitinated form of SRF3 is essential for flg22-mediated immune transcriptional responses, whereas the ubiquitinated form dampens these responses. Super-resolution microscopy revealed that SRF3’s tight coordination of iron homeostasis and immunity is mediated by its ubiquitin-dependent nano-dynamics at the plasma membrane. Finally, SRF3 plays a role in modulating the proliferation of pathogenic and commensal bacteria. Overall, our findings reveal a rapid iron homeostasis mechanism that fine-tunes immune responses to drive the host-microbe interactions.

## RESULTS

### ROS burst triggers a transient decrease of ferrous iron upon flg22 treatment

We set out to explore the mechanism by which the SRF3 receptor kinase links iron homeostasis and defense responses. A previous study had shown that in elongated epidermal root cells, a one-hour treatment with the pathogen-associated molecular pattern (PAMP) flagellin 22 (flg22) reduces the labile pool of Fe^2+^, which represents the usable form of iron within the cell (*16*). PAMP perception, in particular flg22 perception, leads to a massive ROS burst that can involve the oxidation of Fe^2+^ to Fe^3+^ or the reduction of Fe^3+^ to Fe^2+^. Therefore, we hypothesized that the ROS burst links defense responses and cellular iron. To test this, we assessed the kinetics of Fe^2+^ levels upon flg22 treatment using a RhoNox-1-based time-lapse analysis. RhoNox-1 is quantitatively sensitive to different levels of Fe^2+^ in the root epidermis of the elongation zone (*16*). We observed a transient decrease in Fe^2+^ levels starting 10 minutes after flg22 application, continuing to decline until 60 minutes, and then returning to initial levels by 120 minutes, as indicated by the total RhoNox-1 intensity (Fig. 1A). This transient decrease matches the period of the well-documented flg22-induced ROS burst (*17*), thereby supporting our hypothesis that the ROS burst chemically links defense responses and cellular iron. To further test this, we treated roots with the ROS, H_2_O_2_, while monitoring Fe^2+^ and Fe^3+^ levels using RhoNox-1 and the Reversible Fluorescent Fe^3+^ Sensor (RPE), respectively. Upon H_2_O_2_ treatment, the level of RhoNox-1 decreased while RPE fluorescence increased, indicating that iron is being oxidized, with a decrease in Fe^2+^ and an increase in Fe^3+^ levels (Fig. 1B). To further confirm that H_2_O_2_ induces Fe^2+^ oxidation to Fe^3+^ when produced in response to flg22, we repeated the experiment with flg22 treatment. Consistent with the initial findings, we observed a similar trend, showing a reduction in Fe^2+^ levels and an increase in Fe^3+^ levels following the flg22-induced ROS burst (Fig. 1B). To further corroborate these findings, we utilized a genetic approach using loss-of-function mutant lines of the *FLS2* and *RBOHD* genes in which the ROS burst upon flg22 treatment is absent or reduced, respectively, and monitored Fe^2+^ levels using RhoNox-1 staining (*17*). After one hour of flg22 treatment, ferrous iron levels remained unchanged in *fls2* and *rbohD* mutants compared to WT, indicating that ROS production is required for the observed drop in ferrous iron levels (Fig. 1C). Taken together, we revealed that upon flg22 treatment the FLS2-RBOH pathway-mediated ROS burst causes a decrease in ferrous iron.

**Fig. 1.**
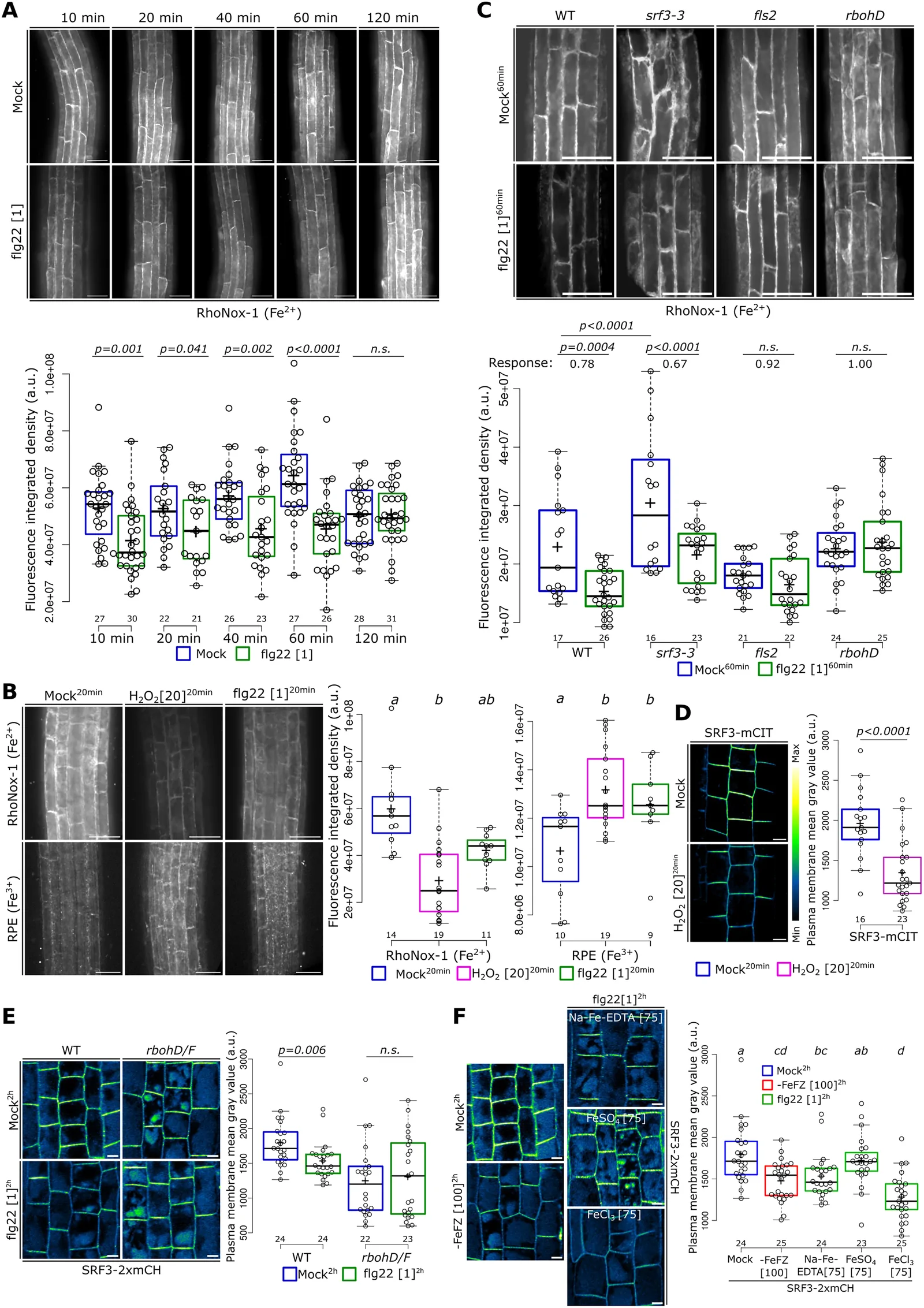
Flg22-induced ROS-driven ferrous iron depletion triggers SRF3 plasma membrane removal and engages SRF3-dependent iron homeostasis regulation. (**A**) Confocal images of RhoNox-1-stained 5-day-old seedlings after mock or flg22 (1 μM, flg22[1]) at the indicated time points, with corresponding quantification (two-way Student’s *t* test, *P* < 0.05). (**B**) Confocal images of 5-day-old seedlings stained with RhoNox-1 or RPE in mock, H_2_O_2_ (20 μM) and flg22 (1 μM, flg22[1]) and with the corresponding quantification [one-way ANOVA, Tukey test, (*P* < 0.05)]. (**C**) Confocal images of RhoNox-1-stained 5-day-old seedlings after mock or flg22 (1 μM, flg22[1]) for 60 minutes for the indicated genotypes and with the corresponding quantification [two-way student’s test (*P* < 0.05)]. (**D**) Confocal images of 5-day-old seedlings expressing SRF3-mCIT, under mock or H_2_O_2_ (20 μM) for 20 minutes and with the corresponding quantification [two-way student’s test (*P* < 0.05)]. (**E**) Confocal images of 5-day-old seedlings expressing SRF3-2xmCH, under mock or flg22 (1 μM, flg22[1]) for 2 hours in WT and *rbohD/F* and with the corresponding quantification [two-way student’s test (*P* < 0.05)]. Pseudo-coloring highlights changes in fluorescence intensity. (**F**) Confocal images of 5-day-old WT seedlings expressing SRF3-2xmCH after 2 hours of mock, -Fe+100 μM ferrozine (-FeFZ[100]), flg22 (1 μM, flg22[1]), or flg22 supplemented with 75 μM Na-Fe-EDTA, FeSO_4_, or FeCl_3_, with corresponding quantification [one-way ANOVA, Tukey’s test, (*P* < 0.05)]. Mock images and the corresponding mock or flg22 quantifications are identical in panels (E) and (F), as both experiments were performed concurrently. Numbers below the box plots indicate individual measurements from one root image, pooled across three independent experiments. n.s., non-significant and letters indicate significant differences. Scale bars, 50 μm [(A) to (C)], 10 μm [(D) to (F)].

### Flg22-induced ROS production mediates low ferrous iron levels and engages SRF3-dependent iron homeostasis regulation

Previously, we demonstrated that *srf3* mutants are partially unresponsive to flg22 and that SRF3 controls iron homeostasis under this condition (*16*). We therefore hypothesized that *SRF3* might regulate Fe^2+^ levels during the flg22-mediated ROS burst. To test this, we evaluated Fe^2+^ levels using RhoNox-1 staining in the *srf3*–*3* mutant upon flg22 treatment. As previously described (*16*), we observed that the roots of *srf3*–*3* seedlings accumulate ectopic iron in standard conditions according to the increased RhoNox-1 fluorescence level (Fig. 1C). Nonetheless, they displayed a strong decrease in Fe^2+^ in response to flg22 treatment compared to WT, yet RhoNox-1 fluorescence level did not reach that of WT levels (Fig. 1C). This indicates that *SRF3* is required to fine tune cellular ferrous iron levels upon flg22 treatment. While it might be possible that *SRF3* regulates the ratio of Fe^2+/^Fe^3+^ upon flg22, we first focused on characterizing *SRF3*’s roles in regulating Fe^2+^ levels. As ROS acts upstream of lowering ferrous iron, we then tested whether the observed increase in iron levels in standard conditions is due to a mis-regulation of ROS in *srf3* mutants. Using Dihydroethidium dye (DHE) to monitor the ROS, superoxide, we did not observe differences between WT and *srf3*–*3*, indicating that *SRF3* does not control ROS levels in standard conditions and therefore cannot explain the observed accumulation of Fe^2+^ (fig. S1A). To test *SRF3*’s role in controlling ROS levels upon bacterial elicitation, we performed ROS measurements in leaves while eliciting immune responses with flg22 in *srf3*–*4* mutant and *SRF3* over-expressing lines. Compared to the *fls2* negative control, both genotypes mounted a ROS burst, albeit significantly reduced in *srf3*–*4* compared to the WT positive control (fig. S1B). Since we observed similar ROS burst trends in plants lacking or over-accumulating SRF3, this indicates that SRF3 might play an indirect role in regulating ROS burst under flg22 treatment as we did not observe a direct correlation between SRF3 levels and ROS production (fig. S1B). Moreover, upon flg22 treatment, the relative decrease in Fe^2+^ levels is more pronounced in *srf3*–*3* compared to WT (0.67 versus 0.78, respectively, Fig. 1C), and the ROS response to flg22 in that mutant is reduced by nearly half relative to WT (fig. S1B). Taken together, our data suggest that *SRF3* does not directly regulate ROS production under either standard or flg22 conditions, nor does it reduce in Fe^2+^ levels; rather, it plays a role in maintaining Fe^2+^ homeostasis upon flg22 perception. Therefore, we concluded that SRF3 might function downstream of ROS production to regulate the ferrous iron pool in response to flg22.

SRF3 plasma membrane levels are decreased in the elongated epidermal root cells upon flg22 and low ferrous iron levels using the strong iron chelator Ferrozine, but not in the control line Lti6b (fig. S1, C and D). Additionally, in this zone, flg22 mediates ROS-dependent reductions in Fe^2+^ levels (Fig. 1, A to C). We set out to test whether SRF3 plasma membrane removal occurred in a ROS-dependent manner. For this, we monitored SRF3 plasma membrane levels using *pUBQ10::SRF3-mCITRINE* (*SRF3-mCIT*) or *pUBQ10::SRF3-2xmCHERRY* (*SRF3-2xmCH*) for which the functionality of the protein fusion has already been demonstrated (*16*). In agreement with our hypothesis, H_2_O_2_ treatment caused a decrease in SRF3 plasma membrane levels (Fig. 1D). We next assessed SRF3 plasma membrane abundance (*SRF3-2xmCH*) in the *rboh-d*,*f* double mutant, which shows only trace ROS levels relative to the *rbohd* single mutant under flg22 treatment (*18*). In contrast to the wild type background, we did not observe a reduction of the SRF3 plasma membrane levels in the *rbohD/F* mutant background upon flg22 treatment compared to mock conditions (Fig. 1E). As such, SRF3 plasma membrane removal upon flg22 treatment requires ROS generation. We note that the initial SRF3 levels in WT and *rbohD/F* differ, as these lines originate from independent transgenic events and may exhibit distinct basal expression levels. Notably, SRF3 appears to accumulate in intracellular compartments resembling the vacuole in *rbohD/F* background. Altogether, these experiments support a model in which the flg22-induced ROS burst triggers a transient decrease in ferrous iron, leading to SRF3 plasma membrane removal. To further test this model, we co-treated roots with flg22 in addition to either chelated iron (Fe-NA-EDTA), ferrous iron (FeSO_4_), which is directly bioavailable, or non-ferrous iron (FeCl_3_), which is not directly bioavailable, and evaluated SRF3 plasma membrane levels. The addition of chelated iron with flg22 caused SRF3 depletion to levels observed under low ferrous iron (-FeFZ[100]), while non-ferrous iron caused a further decrease (Fig. 1F). Importantly, ferrous iron combined with flg22 treatment led to SRF3 plasma membrane levels comparable to the untreated control (Fig. 1F). In summary, we found that (i) flg22 treatment through ROS generation promotes a transient decrease of ferrous iron, (ii) this process is fine-tuned by SRF3 most likely through its role in regulating ferrous iron, and (iii) SRF3 is decreased at the plasma membrane specifically in a Fe^2+^-dependent manner.

### Clathrin-dependent endocytosis controls SRF3 plasma membrane levels upon flg22-induced low ferrous iron

We demonstrated that *SRF3* fine tunes ferrous iron levels upon flg22 treatment independently of an RBOH-dependent ROS burst, and that ROS burst limits ferrous iron and triggers SRF3 plasma membrane removal upon flg22. Moreover, SRF3 levels are dependent on the intracellular ferrous iron levels (Fig. 1F and fig. S1, C and D). To explore whether the decrease in SRF3 at the cell surface contributes to regulating ferrous iron homeostasis during bacterial elicitation, we aimed to identify the cellular mechanisms underlying its reduction at the plasma membrane. Immunoprecipitation of SRF3-mCIT followed by mass spectrometry on root material uncovered the clathrin heavy chain 1 and 2 (CHC1 and CHC2) proteins among the interactors with more than 20 counts (table S1). These proteins are involved in mediating endocytosis of cell surface proteins to control their levels and, therefore, represented strong candidates for mediating SRF3 removal from the plasma membrane (*19*). To test this hypothesis, we assessed in vivo co-localization of SRF3 and clathrin using total internal reflection microscopy (TIRFm). This experiment revealed a partial colocalization of stable diffraction-limited spots between SRF3 and CLATHRIN LIGHT CHAIN 2 (CLC2) a constituent associated with CHC proteins, and was not observed with the unrelated plasma membrane control protein Lti6b in standard conditions (Fig. 2, A and B). To further test whether these proteins are in close proximity (less than 10 nm) in vivo, we performed Förster resonance energy transfer with fluorescence lifetime microscopy (FRET-FLIM) measurements. The fluorescence lifetime of SRF3-mCIT was reduced when co-expressed with CLC2 fused to RFP, compared to single SRF3-mCIT, indicating that these proteins are in close proximity (less than 10 nm, Fig. 2C). Given that SRF3 partially co-localizes with and occurs in close proximity to the clathrin machinery at the plasma membrane, we hypothesized that clathrin controls SRF3 levels at the membrane. To test this hypothesis, we overexpressed an inducible form of *AUXILIN-LIKE 2* (*AX2*), which inhibits clathrin coating by disrupting CHC associations (*20*) and monitored SRF3 levels following *AX2* induction. SRF3 levels increased significantly upon *AX2* induction (fig. S1E), revealing that clathrin-dependent endocytosis modulates SRF3 levels at the plasma membrane. We then conducted additional experiments to investigate whether clathrin-mediated endocytosis is involved in the removal of SRF3 from the plasma membrane in response to low ferrous iron or flg22 treatment, using both genetic and pharmacological approaches. In each approach that disrupted clathrin-mediated endocytosis—such as in the *chc1*–*1* and *chc2*–*3* mutants, or through co-treatment with the clathrin heavy chain (CHC) inhibitor ES9–17 (*21*)—SRF3 reduction was abolished under conditions of low ferrous iron or flg22 treatment, compared to the wild type or mock conditions, respectively (Fig. 2D and fig. S1, F and G). Since endocytosed protein cargos are deposited in intracellular compartments after endocytosis and thus modifying the allocated protein quantity between the plasma membrane and endomembrane located in the cytosol, we evaluated the ratio of the SRF3-mCIT signal between the plasma membrane and the cytosol. Consistent with our previous results, we observed that in *chc1*–*1* and *chc2*–*3* mutants, or upon treatment with ES9–17, the ratio of SRF3 plasma membrane to cytosol intensity was not as strongly reduced as in wild type or mock conditions, respectively (fig. S1, H to J). These findings indicate that SRF3 is removed from the plasma membrane via clathrin-dependent endocytosis in response to flg22-triggered low ferrous iron.

**Fig. 2.**
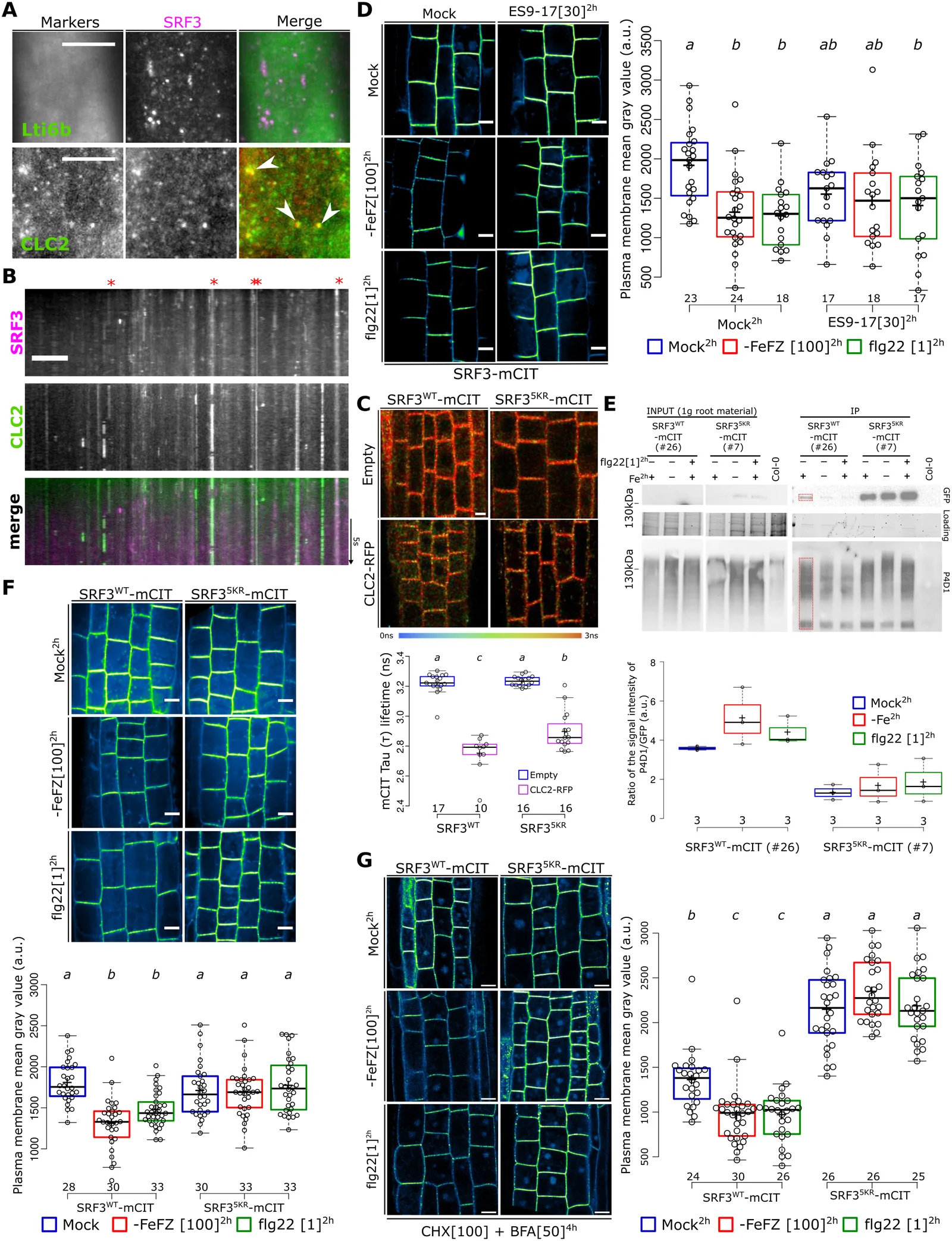
SRF3 ubiquitination initiates clathrin-dependent endocytosis under flg22 and low ferrous iron. (**A** and **B**) Micrographs of 5-day-old seedlings co-expressing SRF3-2xmCHERRY with GFP-Lti6b or CLC2-GFP and merged channels acquired by TIRF microscopy (A) the corresponding kymograph of CLC2/SRF3 (B). Asterisk indicates colocalization. Representative of ≥15 roots per genotype from three independent experiments. (**C**) Representative fluorescence lifetime (τ) images of 5-day-old seedlings expressing SRF3^WT^-mCIT or SRF3^5KR^-mCIT, alone or with CLC2-RFP, with corresponding quantification [Kruskal-Wallis with the Steel–Dwass–Critchlow–Fligner procedure (*P* < 0.05)]. Images are pseudo-colored to highlight mCIT lifetime. (**D**) Confocal images of 5-day-old seedlings expressing SRF3-mCIT, under mock or treated with ES9–17 (30 μM) with +Fe, -Fe+100 μM ferrozine (-FeFZ[100]), and flg22 (1 μM, flg22[1]) for 2 hours and with the corresponding quantification, right [one-way ANOVA, Tukey test, (*P* < 0.05)]. (**E**) Input (left) and immunoprecipitation (IP, right) using anti-GFP or anti-P4D1 from roots of 12-day-old seedlings expressing *pUBQ10::SRF3^WT^-mCITRINE* (*SRF3^WT^*) or *pUBQ10::SRF3^5KR^-mCITRINE* (*SRF3^5KR^*) under control, -Fe or flg22 (1 μM, flg22[1]) for 2 hours. Bottom, quantification of the GFP/P4D1 signal intensity ratio. Red boxes indicate the regions of interest (ROIs) used for quantification. (**F**) Confocal images of 5-day-old seedlings expressing SRF3^WT^-mCIT or SRF3^5KR^-mCIT under mock, -Fe+100 μM ferrozine (-FeFZ[100]) or flg22 (1 μM, flg22[1]) for 2 hours and with the corresponding quantification below [one-way ANOVA, Tukey test, (*P* < 0.05)]. (**G**) Confocal images of 5-day-old seedlings expressing SRF3^WT^-mCIT or SRF3^5KR^-mCIT, after 2 hours of co-treatment with CHX 100 μM and BFA 25 μM with either +Fe or -Fe+100 μM ferrozine (-FeFZ[100]), or flg22 (1 μM, flg22[1]) for 2 hours and with the corresponding quantification, right [one-way ANOVA, Tukey test, (*P* < 0.05)]. Unless otherwise indicated, numbers below box plots represent individual measurements from one root image, pooled across three independent experiments. In (E), numbers indicate independent blots. Scale bars, 5 μm (A) 10 μm [(B) to (G)]. Letters indicate significant differences.

Endocytosis can also occur independent of clathrin (*22*). In plants, this type of endocytosis relies on sterol (*23*). To investigate this, we disrupted sterol homeostasis using a genetic approach (*cpi* mutant) and a pharmacological approach (Lovastatin, LOVA), both of which resulted in increased SRF3 levels at the plasma membrane under standard conditions (fig. S2, A and B). Importantly, pharmacological reduction of cellular sterol did not affect clathrin dynamics (fig. S2C). This demonstrates that sterol-dependent endocytosis can also control SRF3 levels under standard conditions, independently of clathrin. To confirm this, we tested whether clathrin-dependent and -independent endocytosis act synergistically to regulate SRF3 levels. Concomitant inhibition of both endocytosis machineries resulted in additive effects on SRF3 plasma membrane levels, supporting two different endocytosis routes for SRF3 in standard conditions (fig. S2D). We then tested the involvement of clathrin-independent endocytosis in regulating SRF3 levels in response to low ferrous iron or flg22. Interestingly, inhibition of clathrin-independent endocytosis using LOVA did not prevent the decrease in SRF3 levels upon flg22 or low ferrous iron treatments, suggesting that flg22-triggered SRF3 endocytosis primarily operates through clathrin (fig. S2B). In conclusion, our results suggest that under standard growth conditions, SRF3 is endocytosed by both clathrin-dependent and -independent pathways. However, when ferrous iron decreases upon bacterial elicitation, the clathrin-mediated endocytosis becomes the major driver of SRF3 removal from the plasma membrane.

### flg22-triggered SRF3 iron-dependent endocytosis is mediated by ubiquitination

As we had found that SRF3 decrease at the cell surface is mediated via clathrin-dependent endocytosis upon flg22-triggered low ferrous iron, we set out to identify the molecular mechanisms that trigger SRF3 endocytosis. Ubiquitination is one of the most widely used post-translational modifications that can trigger cell surface protein endocytosis minutes after signal perception (*24*)*.* Recently, a large-scale analysis of membrane-associated proteins revealed that SRF6, 7, and 8 are ubiquitinated, and an independent study showed the direct ubiquitination of SRF9 (*25*, *26*). This led us to speculate that SRF3 might be ubiquitinated. Using ubiquitination prediction websites and by aligning its protein sequence with other ubiquitinated SRF family members, we identified five lysine residues (K391, 392, 444, 458 and 471) located in the juxtamembrane domain of SRF3 as probable candidates for ubiquitination (fig. S3A). To test the ubiquitination of these residues and their functional consequences, we generated SRF3 mutant plants (SRF3^5KR^) in which these lysines were substituted with arginines (R), which cannot be ubiquitinated. When conducting an immunoprecipitation of SRF3-mCIT followed by a Western blot with the P4D1 ubiquitin antibody and comparing several independent transgenic lines of wild-type SRF3 (SRF3^WT^) with SRF3^5KR^, we found greater protein accumulation and less relative ubiquitination in SRF3^5KR^ lines (Fig. 2E and fig. S3, C and D), despite similar ranges of gene expression in root cells of SRF3^5KR^ and SRF3^WT^ lines (fig. S3B). However, when we quantified the mean gray values of the fluorescent signal in both lines by confocal microscopy in root tip cells, we did not detect the increased protein abundance (fig. S3E) that we had seen using Western blot in the whole root tissue in SRF3^5KR^ compared to SRF3^WT^ (fig. S3C). We hypothesized that this contrasting result was due to over-accumulation of SRF3^5KR^ in other root zones than the root tip. To test this, we selected the SRF3^WT^ line (#26) which exhibited lower total protein accumulation (fig. S3C) but showed comparable protein levels in the root tip to the SRF3^5KR^ line (#11, fig. S3E). Using these matched lines, we observed that SRF3^5KR^ over-accumulated proteins specifically in the differentiation zone compared to SRF3^WT^ (fig. S3, F and G). This could explain the discrepancy between the total protein abundance of the SRF3 variants and their abundance detected in the root tip. This systematic analysis of SRF3 expression in these lines also revealed that SRF3 protein levels in the root tip remained largely unchanged despite differences in SRF3 transcript abundance (fig. S3, B, C, and E). Previous analyses showed that *SRF3* promoter activity is tightly linked to root developmental status, with little or no expression in the meristematic zone, moderate expression in the transition and elongation zones, and maximal activity in the differentiated zone (*16*). In contrast, SRF3 protein displays the opposite distribution pattern, accumulating predominantly in the root tip [fig. S3F, (*16*)]. These observations suggest that SRF3 protein accumulation in the root tip is regulated largely independently of its transcriptional activity in this zone (*16*). We therefore wondered whether *SRF3* RNA or protein can be cell-to-cell mobile to explain such discrepancy. To do so, we expressed SRF3 fused to mCITRINE under cell-type specific promoters, such as *pWER* (epidermis), *pGL2* (atrichoblast), *pPEP* (cortex), *pATHB8* (columella, stele/vasculature), *pSCR* (endodermis), *pPIN2* (epidermis and cortex), *pCAB3* [shoot, (*27*, *28*)]. Accordingly, we observed that SRF3-mCITRINE was present in almost all root tip tissues, even when expressed specifically in the shoot while being preferentially expressed in the targeted tissue (fig. S3H). This apparent mobility may partly explain why similar SRF3 protein levels are observed in the root tip despite differences in SRF3 transcript abundance.

Next, we evaluated whether SRF3 ubiquitination affects its abundance in membrane associated fractions following low iron and flg22 treatments using Western blot. Unlike SRF3^WT^, SRF3^5KR^ protein levels remained unchanged upon low levels of iron and flg22 (Fig. 2E). Given that SRF3 ubiquitination might affect SRF3 protein abundance in specific root zones, we then assessed SRF3^WT^ and SRF3^5KR^ protein levels in our zone of interest using confocal microscopy. This confirmed that unlike SRF3^WT^, SRF3^5KR^ was not removed from the plasma membrane under low ferrous iron and flg22 conditions compared to control conditions (Fig. 2F). We then tested whether SRF3 removal from the plasma membrane can be mediated by an increased level of SRF3 ubiquitination. Consistent with this hypothesis, we detected an increase of SRF3^WT^ ubiquitination status compared to that of SRF3^5KR^ after exposure to flg22 or low iron treatment (Fig. 2E). Taken together, our results suggest that the five lysines that we identified in SRF3 are ubiquitinated to facilitate SRF3 plasma membrane removal upon flg22 and low levels of ferrous iron. To further corroborate that the ubiquitination of these five lysines is responsible for SRF3 endocytosis, we calculated the SRF3 plasma membrane/cytosol intensity ratio upon flg22 and low levels of ferrous iron. We observed no changes for the SRF3^5KR^ and the control line Lti6b compared to the SRF3^WT^ confirming that these lysine residues are required for the observed SRF3 endocytosis upon flg22 and low levels of ferrous iron (fig. S3I). To further confirm that the observed change in localization between the plasma membrane versus intracellular compartments is due to SRF3 ubiquitination, we generated lines in which SRF3 is artificially ubiquitinated, *pUBQ10::SRF3-mCIT^Ub^*, and the respective negative control that abrogates the recognition of the ubiquitin moiety, *pUBQ10::SRF3-mCIT^Ub-I44A^*. We selected lines that displayed very similar overall protein expression levels in the root tip to ensure that differences observed are independent from this variable (fig. S3, J and K). Consistent with our hypothesis that ubiquitination affects SRF3 plasma membrane to cytosol intensity ratio, we found that SRF3-mCIT^Ub^ was removed from the plasma membrane and targeted intracellularly, while SRF3-mCIT^Ub-I44A^ was indistinguishable from SRF3-mCIT (fig. S3, J to L). These findings demonstrate that artificial ubiquitination can recapitulate native SRF3 ubiquitination, leading to its internalization from the plasma membrane through endocytosis.

Since ubiquitination of protein cargo can engage the clathrin-endocytosis machinery (*29*, *30*), we assessed SRF3^5KR^’s interaction with clathrin compared to SRF3^WT^. FRET-FLIM analysis indicated that the fluorescence lifetime of SRF3^5KR^-mCIT was reduced when co-expressed with CLC2 fused to RFP, indicating proximity in standard conditions (Fig. 2C). However, the decrease in lifetime was significantly less profound compared to SRF3^WT^-mCIT. This suggests that preventing SRF3 ubiquitination may either reduce its interaction with clathrin, thereby impairing SRF3 endocytosis, or that mutation of the five lysines triggers SRF3 conformational changes that may affect its fluorescence lifetime (Fig. 2C).

Because protein ubiquitination status can influence protein recycling from the trans-Golgi network/early endosomal compartments (TGN/EE) to the plasma membrane (*31*), we next tested whether the observed SRF3 plasma membrane regulation upon flg22 treatment and low ferrous iron conditions was linked to its recycling. For this, we first isolated endocytosis from other trafficking processes such as secretion and recycling by co-treating plants with the protein synthesis inhibitor cycloheximide (CHX) and the protein recycling inhibitor, brefeldin A (BFA), respectively. Upon treatment with these inhibitors, SRF3 levels decreased in low ferrous iron conditions and flg22 treatments in the SRF3^WT^ line but not in SRF3^5KR^ and Lti6b control lines, demonstrating the specific importance of SRF3’s ubiquitination in mediating its endocytosis (Fig. 2G and fig. S4A). To further exclude the possible involvement of SRF3 ubiquitination in protein recycling, we conducted three different experiments (i) Brefeldin A (BFA) washout to evaluate the rate of protein recycling from TGN/EE compartments based on the size of BFA bodies, (ii) fluorescence recovery after photobleaching (FRAP) indicating the rate of protein recycling and lateral diffusion at the plasma membrane based on the recovery speed after photobleaching at the plasma membrane, and (iii) co-localization analyses with endosomal markers indicating the endosomal localization. These three independent experiments did not reveal drastic significant differences in BFA body size after washout, recovery speed after photobleaching at the plasma membrane, or colocalization patterns between SRF3^WT^ and SRF3^5KR^ (fig. S4, B to F). Taken together, our data support the conclusion that SRF3 ubiquitination at the five identified lysines does not have a major role in endosomal trafficking but mostly regulates its endocytosis in response to flg22-triggered low ferrous iron levels. Collectively, our results demonstrate that SRF3 plasma membrane levels are regulated through ubiquitination-mediated clathrin-dependent endocytosis when roots are exposed to flg22-induced low ferrous iron.

### SRF3 segregates into mobile and immobile fractions at the plasma membrane in a ubiquitin-dependent manner

The dynamics of ubiquitinated receptor kinases at the plasma membrane have not yet been precisely described at the nanometer scale in eukaryotic systems (*32*). In addition, a recent report mentioned that poly-ubiquitination of cargos can form clusters via phase separation (*33*). Therefore, we set out to study SRF3 ubiquitination in mediating protein nano-organization at the plasma membrane. To do so, we generated two reporter lines: *pUBQ10::SRF3^WT^-mEOS2* and *pUBQ10::SRF3^5KR^-mEOS2*, the latter of which cannot undergo ubiquitination or be removed from the plasma membrane through clathrin-dependent endocytosis upon signal perception. To validate the functionality of the mEOS2 fusion to SRF3, we conducted a root growth assay under low ferrous iron conditions. We observed that two independent lines of *pUBQ10::SRF3^WT^-mEOS2* replicated the behavior to our previously characterized protein fusion with mCITRINE showing that the protein is functional [fig. S5A, (*19*)]. We then analyzed SRF3 plasma membrane nano-dynamics by using photo-activated localization microscopy (PALM) data and coupled it with single particle tracking (Spt-PALM) analysis using these lines (*34*, *35*). Ideally, these experiments would have been performed using complementation lines expressing SRF3 fusion proteins under the control of the native SRF3 promoter. However, fluorescence levels in these lines were insufficient for single-molecule localization and tracking analyses and thus precluded their use for super-resolution microscopy. We therefore used *pUBQ10::SRF3-mEOS2* lines for these experiments. Notably, *pSRF3::SRF3-GFP* and *pUBQ10::SRF3-mCITRINE* displayed nearly identical plasma membrane recovery kinetics in FRAP experiments, indicating that increased SRF3 expression does not substantially alter its membrane dynamics [fig. S4C, (*19*)]. In addition, despite large differences in *SRF3* transcript abundance among independent *pUBQ10::SRF3* lines, SRF3 protein levels in the elongation zone remained nearly identical (fig. S3, C and F). To further assess whether differences in expression affect SRF3 plasma membrane organization, we selected the two independent *pUBQ10::SRF3-mCITRINE* lines that displayed the largest differences in whole-root SRF3 expression (fig. S3C) and quantified diffraction-limited spots by TIRF microscopy. No significant differences in spot density were detected between these lines (fig. S5B). Together, these observations indicate that differences in SRF3 expression levels do not measurably affect its plasma membrane dynamics or organization in the elongation zone, supporting the use of *pUBQ10*-driven SRF3-mEOS2 lines for super-resolution analyses in this tissue. Using Spt-PALM analysis, we then found two SRF3^WT^-mEOS2 populations under basal conditions following a normal distribution above and below a log of a mean square displacement log(D) of −1.67, representing 28% and 72% of the population. These populations are hereafter referred to as the mobile (highly diffusible) and immobile fractions (confined diffusion), respectively (fig. S5C).

We then wanted to investigate the impact of signal perception on these fractions. To do so, we decided to capture early events at 20 minutes after low ferrous iron treatment and flg22 treatments that then could lead to SRF3 plasma membrane removal. We picked this time for several reasons. Ubiquitination and protein cargo internalization by endocytosis occur minutes after signal perception (*36*). In addition, SRF3 plasma membrane decrease becomes significantly apparent after 50 minutes (*16*) suggesting that SRF3 removal from the plasma membrane by ubiquitination-mediated endocytosis occurs prior to this time point. In both conditions, we observed an increase in the immobile fraction of SRF3^WT^ from about ∼70% to ∼90% and a decrease in the mobile fraction from about ∼30% to ∼10% (Fig. 3, A and B and fig. S5D). These results suggest that SRF3 is immobilized when exposed to flg22 or low ferrous iron. As the same treatments also trigger SRF3 clathrin-dependent endocytosis, we hypothesized that SRF3 immobilization in these conditions is mediated by clathrin. To test this, we applied the CHC inhibitor ES9–17, which inhibits clathrin machinery and impacts SRF3 endocytosis upon flg22 and low ferrous iron [Fig. 2D, (*21*)]. We observed no decrease in the mobile fraction when we co-applied ES9–17 with flg22 or with low ferrous iron (Fig. 3C and fig. S5, E and F). Together, our results indicate that SRF3 immobilization in response to flg22-triggered low ferrous iron is dependent on clathrin-mediated endocytosis. As SRF3 is removed from the plasma membrane in a ferrous iron-dependent manner (Fig. 1F) and SRF3 endocytosis impacts its mobility (Fig. 3C), we then set out to evaluate SRF3 mobility in relation to Fe^2+^ levels. To do so, we treated plants expressing *pUBQ10::SRF3^WT^-mEOS2* with flg22 while concomitantly applying FeSO_4_ and evaluated SRF3^WT^-mEOS2 mobility using Spt-PALM. We found that the SRF3 mobile fraction was not decreased when co-treating with flg22 and 75 μM of FeSO_4_ resembling mock conditions compared to flg22 treatment alone (Fig. 3D and fig. S5G). This shows that SRF3 mobility is dependent on ferrous iron levels.

**Fig. 3.**
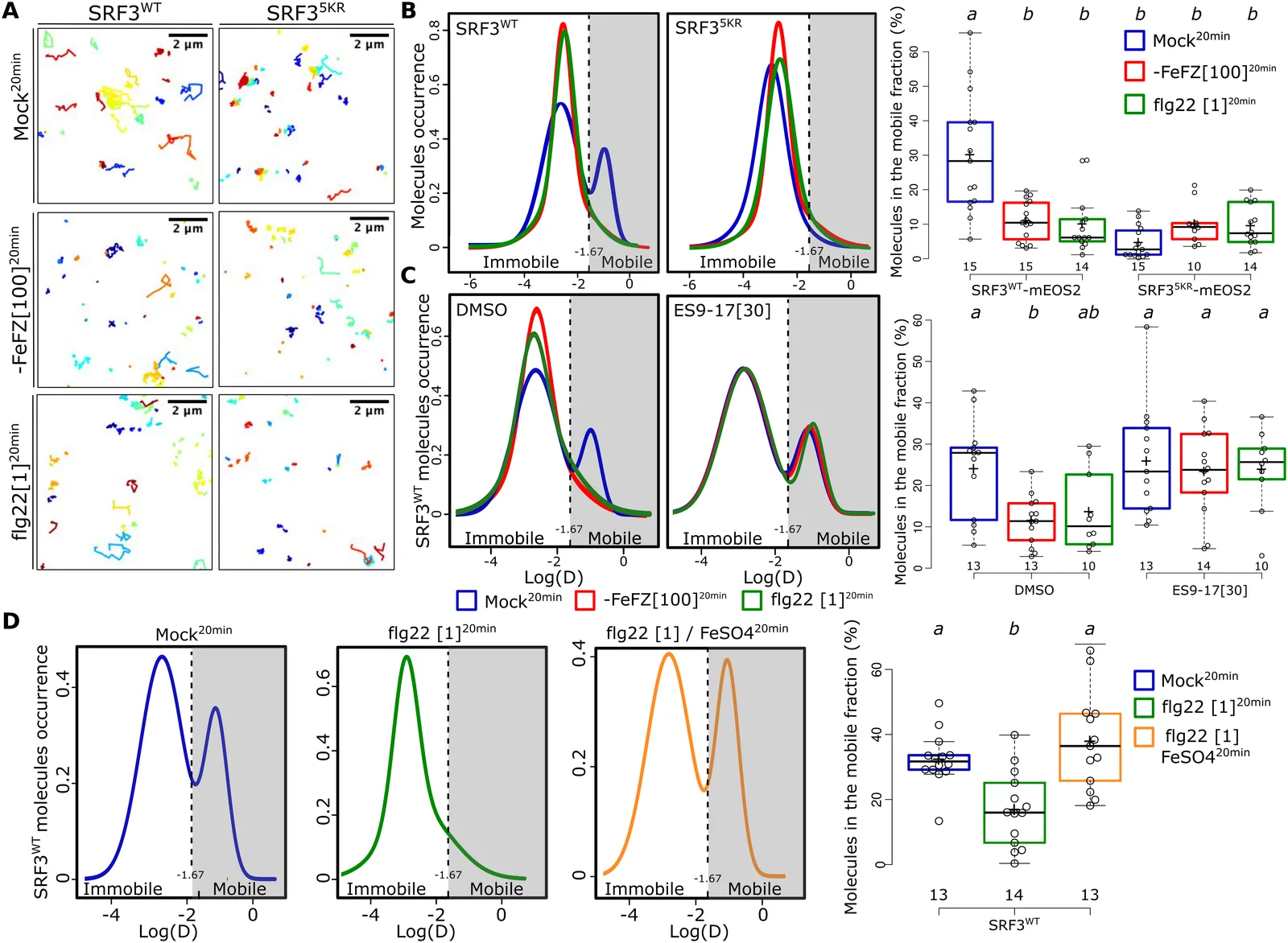
SRF3 ubiquitination mediated endocytosis controls SRF3 nano-dynamics at the plasma membrane. (**A**) Representative trajectories of SRF3^WT^-mEOS2 and SRF3^5KR^-mEOS2 obtained by Spt-PALM analyses in mock treatment, -Fe+100 μM ferrozine (-FeFZ[100]), or flg22 (1 μM, flg22[1]) for 20 minutes. Similar behavior was observed for independent transgenic lines. Scale bars, 2 μm. (**B**) Left, distribution of SRF3^WT^-mEOS2 and SRF3^5KR^-mEOS2 molecules according to their apparent diffusion coefficient D obtained by analyzing Spt-PALM trajectories under mock, -Fe+100 μM ferrozine (-FeFZ[100]), or flg22 (1 μM, flg22[1]) for 20 minutes. Right: quantification of the percentage of molecules in the mobile fraction. [two-way ANOVA, Tukey test, (*P* < 0.05)]. (**C**) Left, distribution of SRF3^WT^-mEOS2 molecules according to their apparent diffusion coefficient D obtained by analyzing Spt-PALM trajectories under mock, -Fe+100 μM ferrozine (-FeFZ[100]), or flg22 (1 μM, flg22[1]) in DMSO (left) or ES9–17 (30 μM, ES9–17[30], right) for 20 minutes. Right, quantification of the percentage of molecules in the mobile fraction. [two-way ANOVA, Tukey test, (*P* < 0.05)]. (**D**) Left, distribution of SRF3^WT^-mEOS2 molecules according to their apparent diffusion coefficient D obtained by analyzing Spt-PALM trajectories under mock, flg22 (1 μM, flg22[1]) or flg22 at 1 μM (flg22[1]) supplemented with either Na-Fe-EDTA (75 μM, Na-Fe-EDTA[75]) or FeSO_4_ (75 μM, FeSO_4_[75]) for 20 minutes and with the corresponding quantification, right [one-way ANOVA, Tukey test, (*P* < 0.05)]. Numbers below the box plots indicate individual measurements from one root image, pooled across three independent experiments. Letters indicate statistical differences.

We then tested the involvement of SRF3 ubiquitination on its membrane diffusion as it controls its endocytosis when exposed to flg22 or low ferrous iron treatments. To ensure comparability of our observations, we used identical microscope settings for SRF3^WT^-mEOS2 and SRF3^5KR^-mEOS2 and subsequently counted almost identical numbers of molecule (SRF3^WT^ = 9060 versus SRF3^5KR^ = 11699 in mock conditions) indicating that the observed differences are not due to different expression levels of the protein (fig. S5, D and H). SRF3^5KR^ was mostly confined to the immobile fraction, representing ∼95% of the total population under basal conditions and in low ferrous iron and flg22 conditions (Fig. 3, A and B and fig. S5H). This result indicates that the ubiquitination of these five lysine residues is critical to sustain SRF3 mobile fraction in a ferrous iron-dependent manner. Therefore, we reasoned that SRF3 ubiquitination plays a dual role depending on the conditions: i) it confers mobility under unstressed conditions, and ii) it promotes endocytosis of SRF3 within immobile fraction upon signal perception. To reconcile these seemingly contradictory observations, one could speculate that signaling alters the type of ubiquitination on these lysine residues (for example, from mono- to polyubiquitination) and/or that ubiquitinated SRF3 interacts with a partner that induces its immobilization.

To conclude, under basal conditions, SRF3 is partitioned into immobile and mobile fractions, with a role of the five ubiquitinated lysines in sustaining the mobile fraction. Upon flg22-triggered low ferrous iron availability, clathrin-dependent endocytosis removes the mobile fraction of SRF3, leading to its localization in the immobile fraction.

### SRF3 is endocytosed from immobile nanoclusters

We showed that flg22 triggers a reduction in ferrous iron levels and decreases the mobile fraction of SRF3 in a clathrin-dependent manner, resulting in the immobile fraction becoming the predominant SRF3 population (Fig. 3, A to C). Therefore, we set out to study the SRF3 immobile fraction upon signal perception in more detail. To do so, we performed a tessellation-based clustering approach using the PALM-generated data to study SRF3 nano-organization at the plasma membrane (*37*). We observed that about ∼10% of immobile SRF3^WT^-mEOS2 was organized in stable nanoclusters that displayed an average diameter of ∼200 nm (fig. S6, A and B). When compared to SRF3^5KR^-mEOS2, a significant increase in cluster density was observed indicating a role of ubiquitination on SRF3 nanocluster density (Fig. 4, A and B and fig. S6, C and D). As ubiquitination mediates SRF3 clathrin-dependent endocytosis (Fig. 2), this suggests that SRF3^5KR^ cluster density increase could be due to defects in clathrin-mediated endocytosis. Consistent with this idea, we observed an increase of the cluster density when we prevented SRF3^WT^-mEOS2 clathrin-dependent endocytosis using ES9–17 (Fig. 4C and fig. S6E). Note that for each genotype and condition approximately the same number of molecules were counted showing that the observed increase in cluster density is not due to an increased accumulation of the protein at the plasma membrane (fig. S6, C to E). The observed increase in nanocluster density is not accompanied by a detectable shift in how SRF3 molecules are distributed between immobile and mobile states (Fig. 3C). This is likely because only about ∼10% of SRF3 molecules cluster in the immobile fraction, yet these immobile molecules account for roughly 70% of all SRF3 signals shown (fig. S6A). Consequently, the reported increase in cluster density under SRF3^5KR^ or ES9–17 represents 2% of the total SRF3 molecules, which corresponds to a very small, effectively undetectable change in the mobile-immobile fractions. Taken together, blocking SRF3’s ubiquitination-dependent endocytosis causes SRF3 to accumulate in nanoclusters, supporting the view that ubiquitinated SRF3 is routed out of some of these clusters via clathrin-mediated endocytosis.

**Fig. 4.**
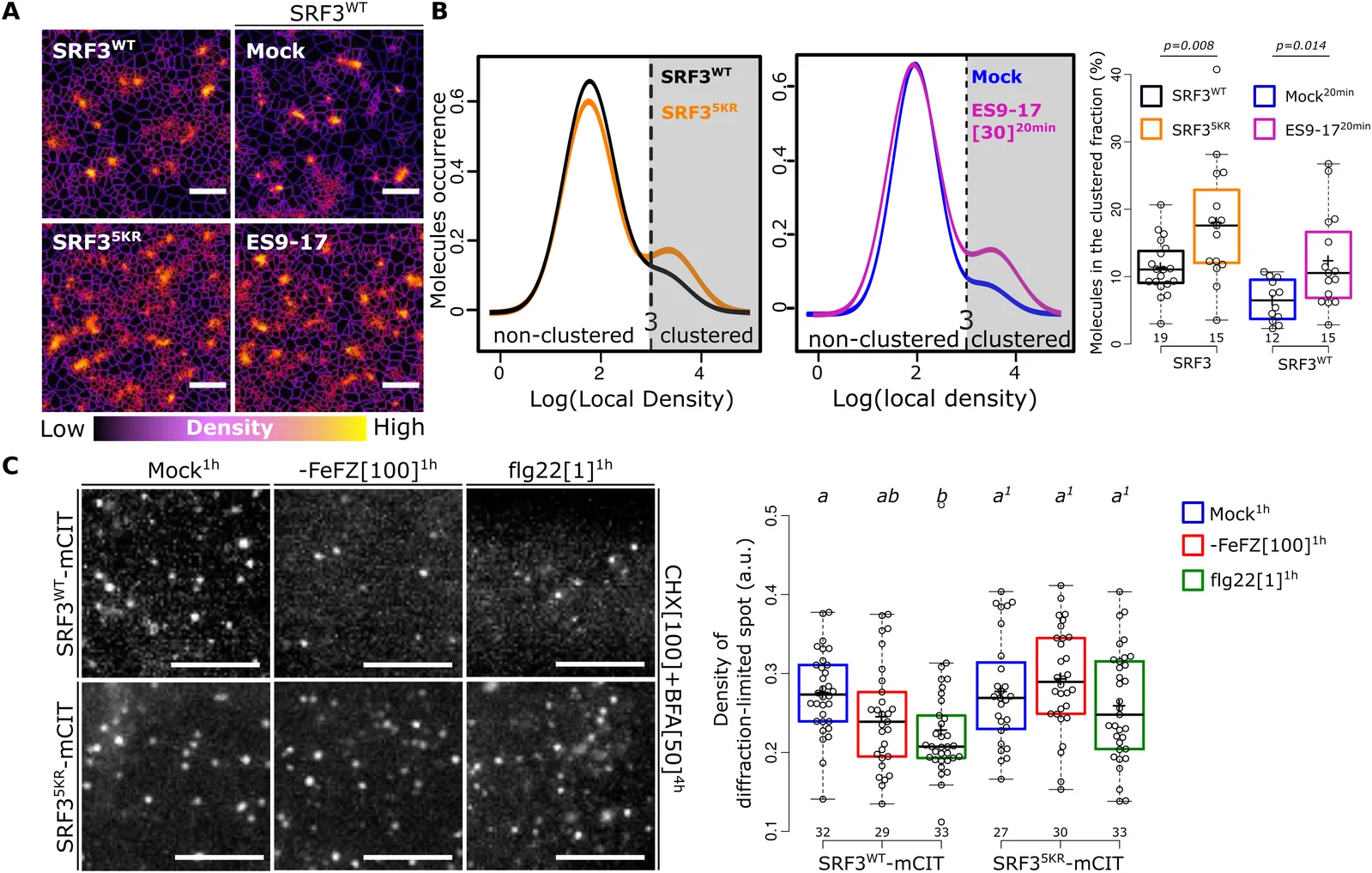
Ubiquitinated SRF3 at the five lysine residues is endocytosed from immobile nanoclusters. (**A**) Representative images of molecules density obtained by tessellation-based automated segmentation of SRF3^WT^-mEOS2 and SRF3^5KR^-mEOS2 molecules and SRF3^WT^-mEOS2 in DMSO or ES9–17 (30 μM, ES9–17[30]) for 20 minutes. Scale bars, 2 μm. (**B**) Left, distribution of SRF3^WT^-mEOS2 and SRF3^5KR^-mEOS2 molecules and SRF3^WT^-mEOS2 in DMSO or ES9–17 (30 μM, ES9–17[30]) for 20 minutes according to their local density under mock. Upper right panel: quantification of the percentage of clustered molecules. [Two-way t-test, (*P* < 0.05)]. (**C**) Micrographs acquired by TIRF microscopy of 5-day-old seedlings expressing SRF3^WT^-mCIT or SRF3^5KR^-mCIT, under cotreatment with CHX 100 μM and BFA 25 μM with either +Fe or -Fe+100 μM ferrozine (-FeFZ[100]), or flg22 (1 μM, flg22[1]) for 1 hour and with the corresponding quantification, right [two-way ANOVA, Tukey test, (*P* < 0.05)]. Scale bars, 5 μm. Numbers below the box plots indicate individual measurements from one root image, pooled across three independent experiments. Letters indicate statistical differences.

As flg22-triggered low ferrous iron conditions engage SRF3 endocytosis, we assessed cluster density upon flg22 and low ferrous iron treatments compared to control conditions. Surprisingly, 20 minutes of treatments did not change the cluster density of SRF3^WT^-mEOS2 compared to mock conditions, possibly suggesting a fast dynamics of SRF3 between endocytosis, secretion and/or recycling in these nanoclusters (fig. S6A). To confirm this assumption, we isolated endocytic events at the plasma membrane using the protein synthesis inhibitor, CHX, and the protein recycling inhibitor, BFA. We then used TIRFm to capture SRF3 endocytic events within the plane of the plasma membrane assessing the number of diffraction-limited spots that remained immobile over time. These measurements include nanoclusters and other immobile molecules at the plasma membrane that cannot be differentiated due to the diffraction limit of ∼250 nm. Since we previously observed that SRF3^WT^ immobile diffraction-limited spots colocalized with clathrin-dependent endocytosis machinery markers (Fig. 2, A and B), we first tested whether when preventing the de novo synthesis or recycling, SRF3-mCIT still co-exists in diffraction-limited spots with clathrin machinery in iron-sufficient conditions. That was indeed the case but not for the negative control REMORIN1.3 (REM1;3, fig. S6F), indicating that the density of diffraction-limited spots represents a readout to evaluate SRF3 endocytosis. When we concomitantly subjected the plants to CHX and BFA and low ferrous iron or flg22, we noticed a decrease in the SRF3^WT^-mCIT diffraction-limited spots, but these differences were not visible for SRF3^5KR^ (Fig. 4C). We found that SRF3 endocytosis triggers the decrease of the diffraction-limited spots in a ubiquitin-dependent manner on its own. When we then performed the same experiment without CHX and BFA, the difference observed for SRF3^WT^-mCIT under low ferrous iron or flg22 was not observable (fig. S6G), consistent with our results from super-resolution cluster analysis (fig. S6A). This indicates that de novo protein synthesis and recycling might be relevant for nanocluster density control in addition to endocytosis. To further corroborate this finding, we used the lines in which SRF3 is artificially ubiquitinated, *pUBQ10::SRF3-mCIT^Ub^*, and the respective negative control that abrogates the recognition of the ubiquitin moiety, *pUBQ10::SRF3-mCIT^Ub-I44A^*. In line with our observations, SRF3-mCIT^Ub^ displayed fewer diffraction-limited spots compared to the control lines SRF3-mCIT^Ub-I44A^ and SRF3-mCIT^WT^ (fig. S6H). Note that for an unknown reason, SRF3-mCIT^Ub-I44A^ density only partially complemented the diffraction-limited spot phenotype compared to SRF3-mCIT^Ub^ and SRF3^WT^ (fig. S6H). Collectively, our data provide evidence that upon signal perception ubiquitinated SRF3 is removed from the mobile fraction and enriched in immobile nanoclusters where its endocytosis occurs through clathrin. This mechanism might underlie the observed overall decrease of SRF3 at the plasma membrane.

### Ubiquitination of five lysine residues in SRF3 is required for root responses to low iron but not to flg22

We identified key residues that mediate SRF3 ubiquitination and upon signal perception promote its removal from the plasma membrane via endocytosis. This provided us with a valuable molecular tool to explore the biological relevance of SRF3 degradation at the plasma membrane in controlling iron homeostasis in response to flg22. To do so, we generated complementation lines in the *srf3*–*3* mutant background: *pSRF3::SRF3^WT^-mCIT* (*SRF3^WT-comp^*), which can be ubiquitinated, and *pSRF3::SRF3^5KR^-mCIT* in *srf3*–*3* (*SRF3^5KR-comp^*), which cannot be ubiquitinated upon flg22 treatment. To allow for a comparison of the effect of ubiquitination independently of the transgene expression levels, we selected lines displaying similar protein levels under control conditions in the root tip (fig. S7A). To assess the functional relevance of SRF3 ubiquitination at the five identified lysine residues, we investigated the ability of *SRF3* complementation lines to rescue *srf3*–*3* phenotypes using developmental, physiological, and molecular readouts. We had observed that adult *srf3*–*3* plants exhibited reduced shoot size, thicker stems, wider branching angles, and darker green rosettes compared with WT plants (fig. S7B). These developmental phenotypes were fully rescued by *SRF3^WT-comp^* but not by *SRF3^5KR-comp^* (fig. S7B).

Given the role of *SRF3* in iron homeostasis, we next quantified iron content in 12-day-old seedlings grown under iron-sufficient or iron-limiting conditions for 7 days. Consistent with previous reports, *srf3*–*3* seedlings accumulated more iron than WT plants under iron-sufficient conditions (fig. S7C). This phenotype was fully complemented by both *SRF3^WT-comp^* and *SRF3^5KR-comp^* lines (fig. S7C). Under iron-limiting conditions, however, *srf3*–*3* seedlings retained higher iron levels than WT plants, and this phenotype was rescued by *SRF3^WT-comp^* but not by *SRF3^5KR-comp^* (fig. S7C). Together, these results demonstrate that the *SRF3^WT-comp^* line is fully functional and suggest that ubiquitination of SRF3 at these five lysine residues contributes to specific SRF3-dependent biological processes.

To further investigate this, we performed RNA-seq analysis on roots of *SRF3^5KR-comp^*, *srf3*–*3*, and WT seedlings grown under basal conditions. Comparison with WT revealed a substantial overlap in differentially expressed genes (DEGs) between *srf3*–*3* and *SRF3^5KR-comp^*, with 40% of upregulated and 50% of downregulated DEGs shared between the two genotypes (table S2). Among the commonly upregulated genes, we identified several genes associated with iron transport and homeostasis, including *IRT1* and the *VACUOLAR IRON TRANSPORTER HOMOLOG* genes *VTL2* and *VTL5*. In contrast, commonly downregulated genes were enriched for functions related to cell division, including microtubule bundle formation and spindle organization (table S2). In addition, a subset of DEGs was specific to *SRF3^5KR-comp^*. Upregulated DEGs were enriched for transport-related processes, including sterol, amino acid, and sulfur compound transport, whereas downregulated genes were associated with defense responses, particularly callose deposition in the cell wall (table S2).

Overall, these transcriptomic data are consistent with the established functions of *SRF3* in iron homeostasis and callose-associated defense responses, while also revealing a distinct transcriptional signature associated with the 5KR variant. Together with the differential complementation phenotypes, these findings suggest that ubiquitination of the five lysine residues contributes to the pathway specificity and function of SRF3 signaling.

To test this hypothesis further, we assessed root growth and transcriptional responses to low iron levels and flg22, as SRF3 has been shown to coordinate these processes (*16*). During the first 12 hours after transfer to low-iron medium, *srf3*–*3* and *SRF3^5KR-comp^* roots showed a smaller reduction in growth than WT and *SRF3^WT-comp^* (Fig. 5, A to F and fig. S7D). This indicates that ubiquitination of SRF3 at the five identified lysine residues is required for proper regulation of root growth in response to low iron. In contrast, early root growth responses to flg22 were rescued in *SRF3^5KR-comp^* plants just like in WT and *SRF3^WT-comp^* compared to *srf3*–*3* plants, indicating that SRF3 ubiquitination on these lysines does not mediate root growth arrest in response to flg22 (Fig. 5, A to F and fig. S7D). To further corroborate this, we assessed responses after three days of exposure to these conditions and found the same pattern of responses (Fig. 5, G and H). Taken together, our data indicate that SRF3 ubiquitination at these five lysines is required for regulating root growth responses to low iron levels but not to flg22.

**Fig. 5.**
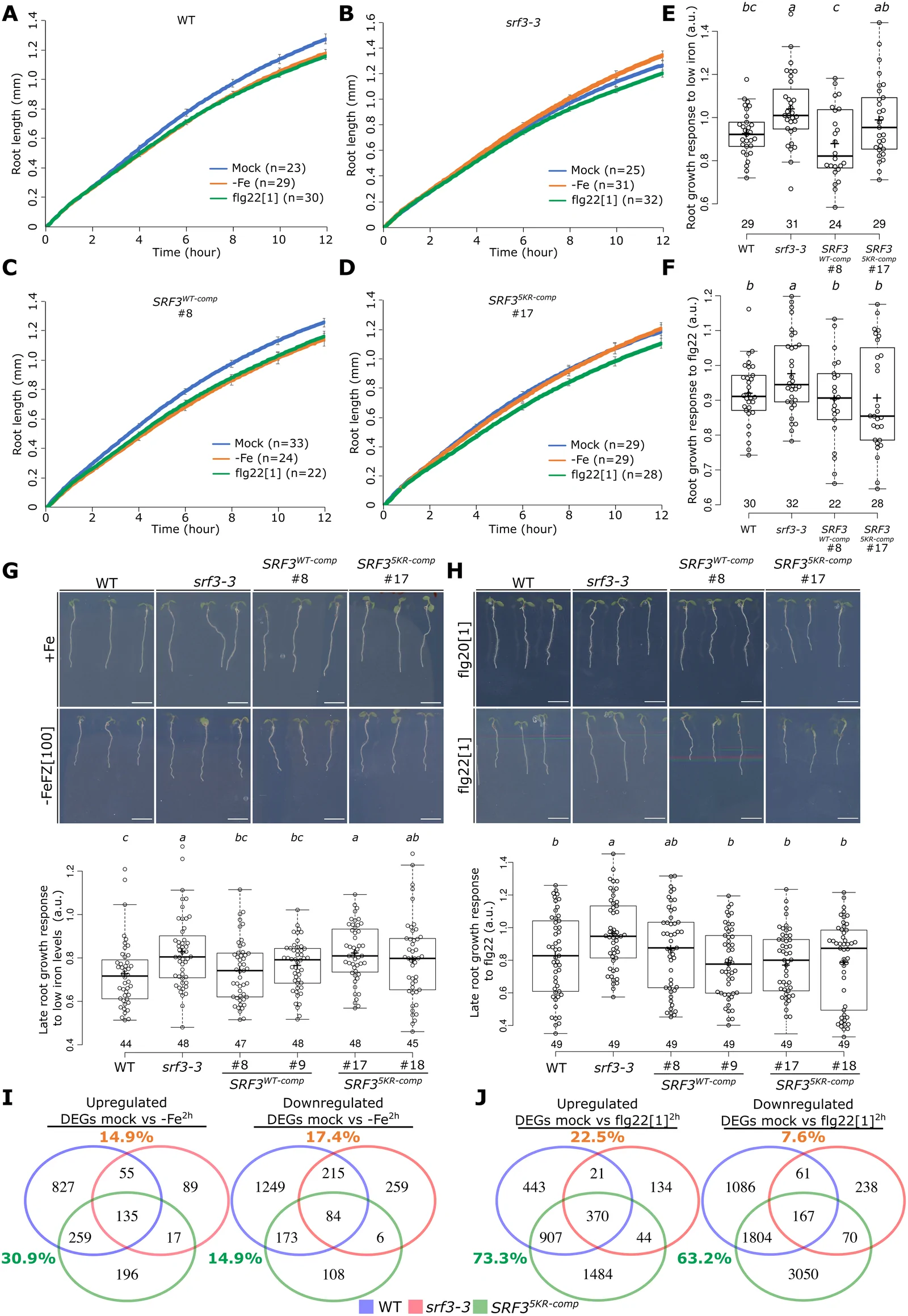
SRF3 ubiquitination at the five lysine residues is required to mediate low iron but not flg22 root responses. (**A** to **F**) Graph showing time-lapse analysis for 12 hours of the root length measurements of WT (A), *srf3*–*3* (B), *SRF3^WT-comp^* (C), and *SRF3^5KR-comp^* (D) under mock, low iron (–Fe) and flg22 (1 μM, flg22[1]). [error bars: standard error of the mean (SEM)] and with the corresponding quantification of the early root growth response to low iron (E) or to flg22 (F). The early root growth response was calculated as the area under the curve (AUC) of each plant under flg22 or low iron, normalized to the mean AUC of control plants. [two-way ANOVA, Fisher test (*P* < 0.1) (E to F)]. Numbers (#) indicate the name of the independent insertion lines. (**G**), Representative images of 8-day-old seedlings of WT, *srf3*–*3*, *SRF3^WT-comp^* and *SRF3^5KR-comp^* grown under standard conditions for 5 days and then transferred for 3 days to iron-sufficient media (+Fe) or -Fe+100 μM ferrozine (-FeFZ[100]) and quantification of the late root growth response. [two-way ANOVA, Fisher test (*P* < 0.05)]. Scale bars, 1 cm. (**H**) Representative images of 8-day-old seedlings of WT, *srf3*–*3*, *SRF3^WT-comp^* and *SRF3^5KR-comp^* grown under standard conditions for 5 days and then transferred for 3 days to media supplemented with flg20 (1 μM, flg20[1]) or flg22 (1 μM, flg22[1]) and the quantification of the late root growth response. Numbers (#) indicate independent insertion lines. [two-way ANOVA, Tukey test (*P* < 0.07)]. Scale bars, 1 cm. (**I** to **J**) Venn diagram of upregulated and downregulated differentially expressed genes (DEGs) under low iron levels (–Fe) for 2 hours (I) or under flg22 (1 μM, flg22[1], J) for 2 hours in WT, *srf3*–*3*, and *SRF3^5KR-comp^*. Numbers below the box plots indicate individual measurements from one root image, pooled across three independent experiments. Letters indicate statistical differences.

The requirement of SRF3 ubiquitination for eliciting root growth responses to low iron, but not to flg22, suggests that SRF3 regulates these responses through distinct signaling pathways. To test this hypothesis, we compared the root growth rate of WT, *srf3*–*3*, and *SRF3^WT-comp^* lines under low ferrous iron, flg22, and low ferrous iron in combination with flg22 treatment. Low iron conditions supplemented with flg22 elicited a stronger response than each single condition or mock treatment, further supporting the existence of two distinct root growth signaling pathways (fig. S7, E and F). In the *srf3*–*3* mutant, this enhanced decrease was not detected, and the complete responsiveness was restored in the *SRF3^WT-comp^* complemented line to the WT levels (fig. S7, E and F). From these experiments, we concluded that SRF3 controls two independent root signaling pathways: one specific to low iron, which is elicited when SRF3 is further ubiquitinated at the five lysines, and the other specific to flg22 signals for which SRF3 ubiquitination at the five lysines is not required.

To further test this model, we performed RNAseq analysis on WT, *srf3*–*3*, and *SRF3^5KR-comp^* lines. Consistent with previous findings (*16*), *SRF3* is critical for regulating transcriptional responses to low ferrous iron and flg22 after 2 hours of treatments. Under low-iron conditions, only 14.9% of upregulated and 17.4% of downregulated DEGs were shared between WT and *srf3*–*3* (Fig. 5I and table S2). Similarly, following flg22 treatment, the overlap between WT and *srf3*–*3* was limited to 22.5% of upregulated and 7.6% of downregulated DEGs (Fig. 5J and table S2). Repeating such an experiment with the *SRF3^5KR-comp^* line revealed only minimal recovery of transcriptome changes observed in the *srf3*–*3* mutant under low iron conditions. We reported 30.9% in *SRF3^5KR-comp^* versus 14.9% in *srf3*–*3* of overlapping DEGs with WT for upregulated genes and 14.9% in *SRF3^5KR-comp^* versus 17.4% in *srf3*–*3* for downregulated genes (Fig. 5I and table S2). However, *SRF3^5KR-comp^* was sufficient to complement a significant portion of the transcriptional responses to flg22 with 73.3% in *SRF3^5KR-comp^* versus 22.5% in *srf3*–*3* of upregulated overlapping DEGs with WT and 63.2% in *SRF3^5KR-comp^* versus 7.6% in *srf3*–*3* of downregulated overlapping DEGs with WT (Fig. 5J and table S2). These results further support the model that SRF3 operates through distinct signaling pathways depending on its ubiquitination status. Note that we have previously found that *SRF3* does not act on immune signaling pathways through the MAPK cascade but on PAMP transcriptional regulation in root tissues (*16*). Therefore, to corroborate the idea of two independent pathways affecting the transcriptional program, we pretreated WT plants for 2 hours with an iron-sufficient or low iron medium and then applied flg22 for 2 hours while monitoring flg22-inducible transcripts of *FLG22-INDUCED RECEPTOR-LIKE KINASE 1* (*FRK1*) and *MYB DOMAIN PROTEIN 51* (*MYB51*). flg22-mediated *FRK1* and *MYB51* induction was elevated upon low iron pre-treatment while no changes were observed for the *UBIQUITIN10* (*UBQ10*) control transcript (fig. S7G). These additive effects further support that both flg22 and low iron signaling act through independent pathways to regulate transcriptional immune responses consistent with our root growth experiments.

Altogether, these results show that SRF3 plays an independent and distinct role in root responses to low ferrous iron or flg22, and that the ubiquitination status at the five lysines specifies the signaling pathway. This further demonstrates that ubiquitination of the five lysines is specifically critical for regulating iron homeostasis responses in roots.

### Ubiquitination of five lysine residues in SRF3 fine-tunes intracellular Fe^2+^ levels upon flg22 perception to modulate immune responses

SRF3 that cannot be ubiquitinated at the five lysines is sufficient to activate flg22 elicited responses, while ubiquitination of these residues is required for the regulation of root responses to low iron levels (Fig. 3). We reasoned that while the SRF3 mediated responses to flg22 and low iron are distinct, they nevertheless converge as flg22 triggers changes in iron homeostasis (Fig. 1). We therefore wanted to investigate this convergence and thus first focused on analyzing iron homeostasis genes upon flg22 treatment in plants impaired in SRF3 ubiquitination. To do so, we conducted a clustering analysis of the transcriptional responses to flg22 and low iron treatments compared to mock in WT, *srf3*–*3* and *SRF3^5KR-comp^* lines (table S3). As ubiquitination is required for responses to low iron and we were interested to identify consequences of the lack of *SRF3*-mediated low iron response in the flg22 response. We therefore focused on genes that strongly responded in *SRF3^5KR-comp^* to flg22 treatment but not in the other conditions or genotypes. Within the 9 clusters, genes in the clusters 2, 3, 6 and 7 responded in a hypersensitive manner to flg22 treatment in *SRF3^5KR-comp^* compared to WT and *srf3*–*3*, while no major differences were apparent under low iron treatment. This suggested that either increased SRF3 levels or SRF3 ubiquitination dampens flg22 responses (table S3). Interestingly, cluster 7 included many known iron homeostasis genes involved in iron transport, storage, signaling, and uptake (Fig. 6, A and B). Note that MYB72 and AHA2 are not in the treatment-vs-mock DEG lists but are members of cluster 7 since they passed the ANOVA filter (FDR < 0.05 across all 9 treatment×genotype groups, Fig. 6, A and B and tables S2 and S3). To test whether this was due to an increased SRF3 protein abundance or specific to its ubiquitination, we checked the expression of these genes in roots overexpressing *SRF3* under flg22. This did not lead to an up-regulation of these genes but rather to their down-regulation (table S4). This indicated that the observed dampening of iron homeostasis genes upon flg22 treatment is due to SRF3 ubiquitination and not its overaccumulation. To confirm that SRF3 ubiquitination is important for the regulation of iron homeostasis under bacterial recognition, we assessed ferrous iron levels in WT, *srf3*–*3*, *SRF3^WT-comp^*, and *SRF3^5KR-comp^* using RhoNox-1 staining after short (45 min) and long exposure (2 hours) to flg22. According to previous results, *srf3*–*3* accumulates ectopic ferrous iron in standard conditions; this defect was complemented in *SRF3^WT-comp^* and to some extent in *SRF3^5KR-comp^* compared to WT when analyzing RhoNox-1 fluorescence levels (Fig. 6C). Interestingly, after 45 minutes of flg22 treatment, the *SRF3^5KR-comp^* line showed a significantly stronger decrease in RhoNox-1 signal intensity than WT (Fig. 6C). This defect was complemented to near WT levels in *SRF3^WT-comp^*, albeit at lower levels, whereas RhoNox-1 signal intensity was strongly reduced in *srf3*–*3* upon flg22 treatment (Fig. 6C). As we previously demonstrated that after 2 hours under flg22 the ferrous iron levels reached initial levels (Fig. 1A), we hypothesized that if SRF3 ubiquitination acts as a positive regulator, the line *SRF3^5KR-comp^* might show defects in establishing proper intracellular levels of ferrous iron at this time point. As expected, WT and *SRF3^WT-comp^* were able to re-establish standard iron levels after two hours of exposure to flg22, whereas *srf3*–*3* or *SRF35^KR-comp^* did not, thus supporting our hypothesis (Fig. 6D). Several distinct mechanisms might underlie these defects, including a slower recovery rate, a larger initial decrease, or an altered homeostatic setpoint. Distinguishing between these possibilities will require further investigation.

**Fig. 6.**
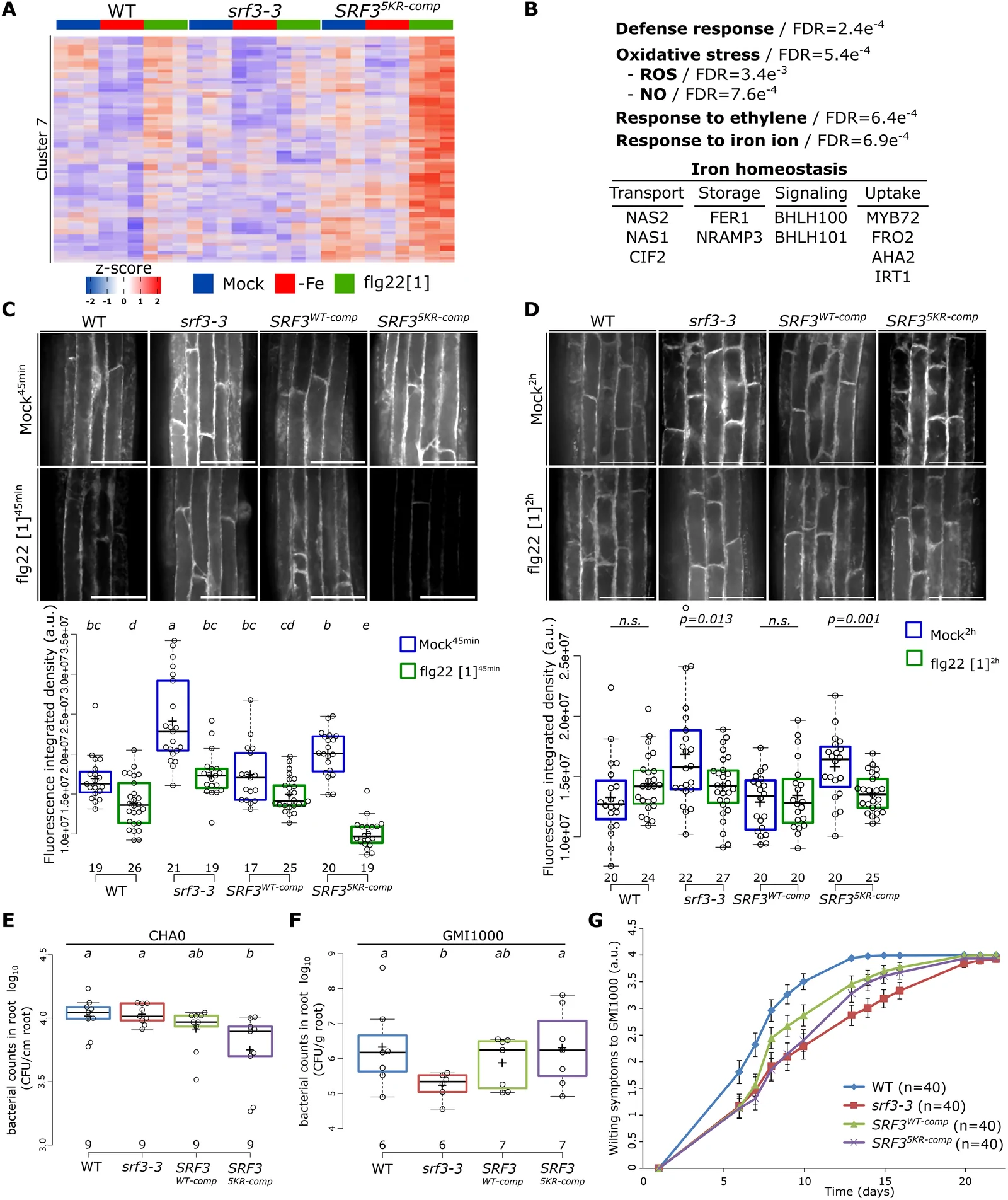
SRF3 balances iron homeostasis and immunity to control plant-microbe interactions. (**A** and **B**) Left, heatmap of cluster 7 obtained after k-means clustering (k = 9; see Materials and Methods and Supplementary Figure R3) on row-z-scored TMM-normalized logCPM expression of the 7,398 ANOVA-filtered genes in WT, *srf3*–*3* and *SRF3^5KR-comp^* under mock, or low iron (-Fe) or flg22 (1 μM, flg22[1]) and (B) with the corresponding gene ontology enrichment analysis depicting the enrichment of defense response, oxidative stress, response to ethylene and iron ion-related differentially expressed genes. (C) Confocal images of 5-day-old seedlings stained with RhoNox-1 in mock (upper panel) or flg22 (1 μM, flg22[1]), lower panel, for 45 minutes for the indicated genotypes and with the corresponding quantification, right [two-way ANOVA, Tukey test, (*P* < 0.05)]. Scale bars, 50 μm. (D) Confocal images of 5-day-old seedlings stained with RhoNox-1 in mock (upper panel) or flg22 (1 μM, flg22[1]), lower panel, for 2 hours for the indicated genotypes and with the corresponding quantification, right [two-way t-test (*P* < 0.05)]. Scale bars, 50 μm. (E) Colonization of 7-day-old *Arabidopsis* roots at 1 day after inoculation by *Pseudomonas protegens*, CHA-0 under mock conditions in the indicated genotypes. Three biological replicates. Data are mean ± s.e.m. [one-way ANOVA, Tukey’s test, (*P* < 0.05)]. (F) Colonization of 7-day-old *Arabidopsis* roots at 5 days after inoculation by *Ralstonia solanacearum* GMI1000 in the indicated genotypes. Two biological replicates. Data are mean ± s.e.m. [one-way ANOVA followed by Fisher’s test (*P* < 0.05)]. (G) Wilting symptoms of the indicated genotypes after inoculation by *R. solanacearum*, GMI1000, under mock conditions. Two biological replicates presented similar trends. Data are mean ± s.e.m. Numbers below the box plots indicate individual measurements from one root image, pooled across three independent experiments. n.s., non-significant. Letters indicate statistical differences.

Interestingly, we also noticed that among the clusters that responded in a hypersensitive manner, clusters 6 and 7 included genes related to defense, oxidative stress, and ethylene responses, showing that SRF3 ubiquitination acts as a dampener on a broad range of immune-related processes (Fig. 6, A and B and table S3). To confirm this, we evaluated the callose deposition using aniline blue staining in the epidermal root cells of the transition/elongation zone. We chose this readout as *SRF3* has been previously characterized to regulate callose deposition at the plasma membrane under low iron levels in this zone and that flg22 is known to trigger callose deposition 30 minutes in leaves or 18 hours in differentiated cells of root tissues after perception of flg22 (*16*, *38*, *39*). We first investigated whether the SRF3^5KR^ variant, similarly to SRF3^WT^, localizes to plasmodesmata. To assess this, we analyzed the characteristic punctate localization pattern of SRF3 using fluorescence intensity profile analyses, a method previously established to reveal protein enrichment at plasmodesmata (*16*). We observed that the intensity plot profile of SRF3^WT^ was similar to that of SRF3^5KR^ presenting higher variation (X2) than the negative control Lti6b (fig. S7H). This indicated that the lack of the 5 identified lysines does not impair SRF3 localization at the plasmodesmata. We then evaluated the callose deposition response to flg22 based on the ratio of the aniline blue signal intensity under flg22 to the mean of the aniline blue signal intensity under mock conditions. Surprisingly, 2-hour treatment with flg22 induces a slight decrease in callose deposition in the WT as the response is below the value of 1. However, in *srf3*–*3* this decrease was not visible and was partially complemented in *SRF3^WT-comp^*, whereas in *SRF3^5KR-comp^* an even lower level of callose deposition was observed compared to the WT (fig. S7I). Importantly, this enhanced reduction in *SRF3^5KR-comp^* is consistent with our RNA-seq analysis, which revealed that disruption of SRF3 ubiquitination at these five lysine residues is associated with the downregulation of genes involved in callose-dependent processes. In line with our hypothesis, this suggests that SRF3 ubiquitination is a repressor of immune transcriptional responses. Note that the discrepancy of callose dynamics under flg22 in leaf and root tissues remains to be elucidated and is discussed below. To conclude, our results show that SRF3 ubiquitination has a dual role: it positively controls ferrous iron homeostasis and negatively regulates immune genes upon treatment with flg22.

### *SRF3* and its ubiquitination shape plant-microbe interactions

We demonstrated that SRF3 lysine ubiquitination plays a role in fine tuning immunity and iron homeostasis. It is known that the coordination of these pathways is pivotal for nutritional immunity responses, which determine the infection outcome upon host-microbe interaction (*4*). Therefore, we set out to investigate the role of SRF3 in host-microbe interactions. We reasoned that it would be most meaningful to perform these assays under iron-sufficient levels as, (i) iron levels and ratio of ferric and ferrous iron form can directly affect the proliferation of the bacteria *per se* (*40*), (ii) *SRF3* is critical for the regulation of canonical iron genes, such as *IRT1* (*16*) which will impact the initial iron levels in the different genotypes if submitted to low iron conditions and (iii), iron deficiency or excess can impact the ROS burst that triggers the decrease of Fe^2+^ that we characterized in this study. For this, we used WT, *srf3*–*3* mutant, *SRF3^WT-comp^* and *SRF3^5KR-comp^* and first treated plants with the root specific commensal bacteria *Pseudomonas protegens* CHA-0 strain in iron-sufficient conditions. This strain is known to trigger a ROS burst upon perception of the canonical flagellin by the host (*41*). Therefore CHA-0 is an appropriate strain to query SRF3 roles in influencing host-microbe interactions. One day after inoculation, *srf3*–*3* mutant plants exhibited bacterial counts in root tissues comparable to those of the WT and *SRF3^WT-comp^* lines (Fig. 6E). In contrast, the *SRF3^5KR-comp^* line exhibited decreased bacterial levels on the root surface indicating that SRF3 is involved in controlling commensal bacteria levels, which is dependent on its lysine ubiquitination (Fig. 6E).

We then performed similar experiments using the bacterial root pathogen: *Ralstonia solanacearum* strain GMI1000. *R. solanacearum* does not trigger a canonical flg22 signal response, but its presence engages a host ROS burst (*42*) and therefore will affect iron homeostasis (Fig. 1). Unlike the previous experiment, where plants were grown in liquid media and bacterial loads were measured one day after inoculation, in this experiment the plants were grown on agar plates, and bacterial loads were assessed five days after inoculation because the infection progressed more slowly under these conditions. In this condition, we observed that roots of *srf3*–*3* mutant contained lower bacterial loads compared to the WT, which was partially or fully complemented in the lines *SRF3^WT-comp^* and *SRF3^5KR-comp^*, respectively (Fig. 6F). To determine the stability of these phenotypes, we inoculated older plants in soil with *R. solanacearum* and recorded the wilting symptoms for 25 days. Here, we recorded wilting of vegetative tissues resulting from *R. solanacearum* clogging the root vascular system. After five days of inoculation, *srf3*–*3*, *SRF3^WT-comp^* and *SRF3^5KR-comp^*, remained less sensitive to *R. solanacearum* compared to WT. However, about 12 days post inoculation, we observed that only *srf3*–*3* displayed less sensitivity to the pathogenic bacteria, while *SRF3^WT-comp^* and *SRF3^5KR-comp^* showed partial complementation compared to WT symptom levels (Fig. 6G). These results support that *SRF3* is involved in controlling pathogenic bacterial proliferation independently of lysine ubiquitination.

Together, these results further underline that *SRF3* and SRF3 ubiquitination shape plant-microbe interactions.

## DISCUSSION

Building on earlier work identifying SRF3 as a coordinator of plant immunity and iron homeostasis, we uncovered a mechanistic framework that links early immune signaling to rapid control of intracellular ferrous iron availability. We show that pattern triggered ROS production imposes an acute and transient state of ferrous iron limitation, thereby coupling pathogen perception to nutritional immunity. Within this window, SRF3 emerges as a molecular integrator that simultaneously sustains immune signaling and safeguards intracellular ferrous iron levels during the oxidative burst elicited by bacterial challenge. This functional duality is encoded in the nano organization of SRF3 at the plasma membrane, where its ubiquitination status governs clathrin-dependent endocytosis and thereby partitions distinct signaling outputs. Through this mechanism, SRF3 modulates both the magnitude of immune responses and the proliferation of commensal and pathogenic bacteria (fig. S8). Together, our findings reveal how a single receptor kinase uses ubiquitin dependent spatial reorganization to balance iron restriction with essential cellular functions, thereby shaping host microbe interactions during the earliest stages of immune activation.

Exposure of roots to flg22 leads to its recognition through direct binding to the leucine-rich repeat (LRR) receptor kinase FLS2 and its co-receptor BRASSINOSTEROID INSENSITIVE 1-ASSOCIATED KINASE 1 (BAK1), initiating a phosphorylation cascade that activates RBOHs which mediate the ROS burst (*17*, *43*). We discovered a direct link between this pattern-triggered immunity pathway and iron homeostasis through the generation of ROS (Fig. 1). We found that H_2_O_2_ that is generated upon ROS burst causes a transient intracellular decrease of ferrous iron (Fig. 1). This phenomenon might be explained by H_2_O_2_-induced ATP-dependent intracellular iron efflux as previously observed in barley during pathogen attack (*44*). Alternatively, extracellularly produced H_2_O_2_ can enter cells through aquaporins, where it may reduce intracellular ferrous iron levels via the Fenton reaction (*45*). We demonstrated that *SRF3* might have an indirect or minor role in the ROS production (fig. S1, A and B), likely reflecting its function in regulating cellular iron levels, which are known to shape ROS dynamics (*46*). In turn, SRF3 directly participates in the regulation of immunity through its impact on transcriptional programs and growth processes (Fig. 5 and tables S2 and S3). Here we demonstrate that SRF3 in a non-ubiquitinated state at the five lysine residues is sufficient to mediate immune responses, whereas ubiquitinated SRF3 acts as a repressor of immune responses (Fig. 5 and tables S2 and S3). The control of SRF3 on these responses is most likely regulated by its interaction with BAK1 as found in multiple species, barley, rice, and potato in leaves (*47*–*49*). We also found that the ubiquitination status involving the five identified lysine residues in SRF3 is required to regulate transcriptional and growth responses to low iron, as both responses were impaired in the SRF3 mutant line in which ubiquitination at these sites is prevented, compared to WT (Figs. 5, 6, A and B, and tables S2 and S3). Consistent with the involvement of SRF3 ubiquitination in iron homeostasis, we revealed its role in regulating the transient decrease of ferrous iron (Fig. 6, C and D). This shows that in addition to its role in repressing immune genes and growth, SRF3 ubiquitination fine tunes these processes by its role in regulating iron homeostasis under flg22. This sheds light on a direct molecular regulation of immune responses by iron homeostasis through SRF3 ubiquitination.

As SRF3 ubiquitination is central to the regulation of iron homeostasis–dependent immunity, we therefore focused on further characterizing its molecular function at the cellular level. We established that SRF3 ubiquitination on the lysines 391, 392, 444, 458 and 471 leads to its specific clathrin-dependent endocytosis in a ferrous iron-dependent manner upon flg22 that decreases its levels at the plasma membrane (Fig. 2 and figs. S1, E to J and S3). The SRF3 clathrin-dependent endocytosis seems to be a shared mechanism between dicots and monocots as SRF3 interacts with the known clathrin-dependent regulator SAC9 in barley (*49*). This led us to proceed to an in-depth characterization of SRF3 ubiquitin-dependent endocytosis using super resolution microscopy. Through this nanoscopic analysis we revealed a tripartite role of SRF3 depending on its diffusion and the ubiquitination status of the five lysines: 1) SRF3 is endocytosed when ubiquitinated from immobile nanoclusters where it represses immunity and participates in the regulation of low ferrous iron levels, 2) when SRF3 is immobile and non-ubiquitinated it engages flg22 transcriptional responses and 3) when SRF3 is ubiquitinated and mobile it participates in sensing intracellular low ferrous iron levels.

Super resolution microscopy revealed that SRF3 ubiquitin-dependent endocytosis takes place in immobile nanoclusters at the plasma membrane upon flg22-triggered low ferrous iron levels (Fig. 4 and fig. S6). According to previous findings, clathrin-dependent endocytic events occur in membrane immobile fractions and were recently shown to form biomolecular condensates allowing spatial sequestration of clathrin machinery in plants (*50*).This supports that SRF3 clathrin-dependent endocytosis is mediated in dedicated regions of the plasma membrane such as nanoclusters. Whether these SRF3 containing nanoclusters represent *bona fide* biomolecular condensates remains to be determined.

In addition, our data indicate that SRF3 can become immobilized, either within nanoclusters or independently of them, without requiring ubiquitination, as shown by the tessellation-based analysis using the SRF3^5KR^ version (Fig. 4 and fig. S6A). The non-ubiquitinated forms of SRF3 are sufficient to activate immune signaling when plants are exposed to flg22 (Fig. 5) suggesting that this form is dedicated to the activation of immune signaling. Interestingly, the flagellin receptor FLS2 signals from similar immobile regions at the plasma membrane (*51*). We described that upon flg22-triggered low ferrous iron, SRF3 is further ubiquitinated and enriched in nanoclusters to be endocytosed (Figs. 2E and 4, A and B). This suggests that signal perception may trigger the ubiquitination or polyubiquitination at the five lysine residues of immobile SRF3 pools, promoting their internalization either through direct endocytosis of existing clusters or via cluster assembly prior to endocytosis. The idea is consistent with the dynamics observed for animal receptors such as EGFR which upon ligand perception with EGF becomes further enriched in nanoclusters and ubiquitinated depending on EGF concentration (*52*, *53*).

Using single particle tracking analysis, we revealed that the above characterized immobile fraction accounts for about 70% of total SRF3 at the plasma membrane while 30% are localized in the highly diffusible region of the membrane (Fig. 3, A and B). In accordance with the proposed role of SRF3 in taking part in iron sensing (*16*), we shed light on the role of ubiquitinated SRF3 mobile fraction in this process. SRF3 mobility varies depending on the intracellular ferrous iron content (Fig. 3D). However, the direct molecular mechanisms by which SRF3 senses low ferrous iron levels remain to be elucidated. In the context of our study, it is tempting to speculate that the activation of a mono- or polyubiquitin E3 ligase may be involved, with the proteins BRUTUS and BRUTUS-LIKE 1 and 2 as plausible candidates given their established roles in iron sensing (*54*). In addition, as SRF3 is a LRR receptor kinase it can bind extracellular low iron-generated signals that could participate in iron sensing. While the direct ligand remains to be discovered, it might be linked to ROS production triggering changes in the Fe^2+/^Fe^3+^ ratio.

Previously, it was demonstrated that SRF3 is critical to regulate callose deposition under low iron levels via the regulation of CALLOSE SYNTHASE 3 (CalS3). In addition, SRF3 and CalS3 were shown to co-localize in diffraction-limited domains at the plasma membrane (*16*). Together with our current observations, these findings suggest that SRF3 may reside within relatively immobile plasma membrane regions that are not necessarily organized into detectable nanoclusters (Fig. 4 and fig. S8).

We also previously showed that SRF3 accumulates at plasmodesmata and that low ferrous iron conditions reduce its abundance at these sites, thereby promoting callose deposition (*16*). In the present study, however, we did not investigate the relationship between SRF3 nanodynamics at the plasma membrane, its localization at plasmodesmata, and its signaling activity. Addressing this question remains technically challenging. Indeed, TIRF microscopy and subsequent Spt-PALM analyses are restricted to the lateral plasma membrane of root epidermal cells, while plasmodesmata are primarily located at the apical and basal interfaces between adjacent cells. Despite these limitations, our results further support a role for SRF3 in regulating callose-associated processes. Transcriptomic analyses revealed that disruption of SRF3 ubiquitination at the five identified lysine residues altered the expression of genes associated with callose deposition and cell wall defense responses (table S2). In addition, flg22 treatment for 2 h induced a modest reduction in callose levels in WT plants and a more pronounced reduction in *SRF3^5KR-comp^* lines (fig. S7I). One possible explanation is that impaired ubiquitination affects SRF3 removal from plasmodesmata, thereby maintaining its repressive activity on callose deposition following flg22 perception. However, direct evidence for this mechanism will require further investigation. At first glance, these observations appear inconsistent with previous reports showing increased callose accumulation following flg22 treatment. However, important differences in both spatial and temporal resolution likely account for these contrasting results. Previous studies quantified callose patches throughout the cell wall of root elongation zone after prolonged flg22 exposure [approximately 18 hours, (*39*)], whereas our analysis focused on plasma membrane-associated callose in epidermal cells of the elongation zone after only 2 h of treatment. These distinct experimental conditions likely capture different phases of the response, with our study reflecting early signaling events and previous studies measuring later responses.

Interestingly, low iron conditions and flg22 appear to exert opposite effects on callose deposition despite both engaging SRF3-dependent signaling pathways (*16*). This observation suggests that distinct regulatory modules may operate downstream of SRF3 depending on the environmental conditions (Fig. 5, fig. S7, E to G, and table S2). Through this context-dependent regulation, SRF3 may contribute to maintaining callose levels compatible with the propagation of early signaling events, including RBOH-dependent signals, while simultaneously coordinating defense responses (*55*). Thus, as an integrator of iron homeostasis and immune signaling, SRF3 likely plays a central role in the dynamic regulation of callose deposition, although the underlying molecular mechanisms remain to be elucidated.

Balancing iron levels is critical for cell survival upon bacterial presence where cells need to restrict iron levels but maintain sufficient levels to sustain vital function for engaging immune responses and sustaining a functional metabolism. In this study, we identify SRF3 as a key factor maintaining this fine balance and as critical for shaping host-microbe interaction. In the presence of the root commensal bacteria, *Pseudomonas protegens*, CHA0, we revealed the role of SRF3 ubiquitination as a negative regulator of bacterial growth while no defects were observed in *srf3*–*3* mutants (Fig. 6E). This suggests that either the elevated immune activation and/or the reduced levels of ferrous iron are responsible for repressing the growth of the commensal *Pseudomonas protegens* CHA-0 strain when SRF3 is not ubiquitinated at the five lysines (Fig. 6E). However, the more likely explanation is that plants contain lower levels of ferrous iron, as this bacterium is particularly adept at overcoming host immune responses and does not repress the iron uptake machinery while residing on the root cell surface (*14*, *56*). Although the details could not be fully resolved in this study, it highlights the role of SRF3 ubiquitination in regulating bacterial proliferation. Additionally, we examined the proliferation of a root pathogenic bacterium, *R. solanacearum* GMI1000. Our findings revealed that the absence of *SRF3* suppresses the progression of the infection throughout the plant, regardless of its ubiquitination status (Fig. 6, F and G). This suggests that the *R. solanacearum* effectors might take advantage of SRF3’s role in coordinating immunity and iron homeostasis to fuel bacterial virulence. The results found in this study are in full agreement with recent independent reports that confirm the role of SRF3 in establishing host-microbe interactions through balancing iron homeostasis and immunity. For example, the powdery mildew virulence effector AVR_A13_ targets the intracellular region of SRF3 and AVR_A13_ desensitizes fungal susceptibility to high iron levels (*49*). In addition, the effector ustilaginoidin synthetase (UgsL) regulates the virulence of the rice false smut fungus *Ustilaginoidea virens* by direct interaction with SRF3 and by preventing SRF3 phosphorylation by BAK1 (*47*). Together, the data underline the critical role of SRF3 in establishing host-microbe interactions and here we reveal that SRF3 and its regulatory role in nutritional immunity is conferred by its ubiquitination and nano-organization.

## MATERIALS AND METHODS

### Plant materials and growth conditions

For surface sterilization, *Arabidopsis thaliana* seeds that had been produced under uniform growth conditions and were placed for 1 hour in opened 1.5-mL Eppendorf tubes in a sealed box containing chlorine gas generated from 130 mL of 10% sodium hypochlorite and 3.5 mL of 37% hydrochloric acid. For stratification, seeds were imbibed in water and stratified in the dark at 4°C for 2–3 days. Seeds were then put on the surface of Fe-sufficient media described in (*16*) and named Gruber media, it contains, 750 μM of MgSO_4_-7H_2_O, 625 μM of KH_2_PO_4_, 1000 μM of NH_4_NO_3_, 9400 μM of KNO_3_, 1500 μM of CaCl_2_-2H_2_O, 0.055 μM of CoCl_2_-6H_2_O, 0.053 μM of CuCl_2_-2H_2_O, 50 μM of H_3_BO_3_, 2.5 μM of KI, 50 μM of MnCl_2_-4H_2_O, 0.52 μM of Na_2_MoO_4_-2H_2_O, 15 μM of ZnCl_2_, 75 μM of Na-Fe-EDTA (sufficient media) or 0 μM of Na-Fe-EDTA (low iron media), 1000 μM of MES adjusted to pH 5.5 with KOH. The *srf3*–*3* (*SALK_202843*), *srf3*–*4* (SAIL_1176_B01), *chc1*–*1* (Salk_112213), *chc2*–*3* (Salk_059016), *fls2* (SAIL691_C04), *rbohD* (N68747), *rbohD/F* (N68522), *cpi1*–*1* out-crossed in Col-0 background, *XVE> > AX2*, mutant lines are in Col-0 background and were described and characterized (*16*, *20*, *57*, *58*). The reporter lines, *pCLC2::CLC2-GFP*, *pRPS5a::CLC2-RFP*, *pAP2u::AP2u-GFP*, *pREM1.3::REM1.3-GFP*, *pSRF3::SRF3-mCITRINE/srf3*–*3*, *pUBQ10::SRF3-mCITRINE*, *pUBQ10::SRF3-2xmCHERRY*, *2x35s::GFP-Lti6b*, *pUBQ10::mCHERRY-VTI12*, *pUBQ10::mCHERRY-RabF2a*, *pUBQ10::mCHERRY-RabA5d pVHA-A3::VHA-A3-RFP* are in Col-0 background and were described and characterized (*16*, *59*–*62*). Plants were grown in long day conditions (16/8 hours) in walk in growth chambers at 21°C, 50 uM light intensity, 60% humidity. During the nighttime, temperature was decreased to 15°C.

### Plant transformation and selection

Each construct (see below: “Construction of plant transformation vectors (destination vectors) and plant transformation”), was transformed into the C58 GV3101 *Agrobacterium tumefaciens* strain and selected on YEB media (5 g/L beef extract; 1 g/L yeast extract; 5 g/L peptone; 5 g/L sucrose; 15 g/L bactoagar; pH 7.2) supplemented with antibiotics (Spectinomycin, Gentamycin). After two days of growth at 28C, bacteria were collected using a single-use cell scraper, re-suspended in about 200 mL of transformation buffer (10 mM MgCl2; 5% sucrose; 0.25% silwet) and plants were transformed by the floral dipping method. Plants from the Columbia–0 (Col-0) accession were used for transformation. Primary transformants (T1) were selected in vitro on the appropriate antibiotic/herbicide (glufosinate for mCITRINE and mEOS, Hygromycin for mCHERRY). Approximately 20 independent T1s were selected for each line. In the T2 generation at least 3 independent transgenic lines were selected using the following criteria when possible: (i) good expression level in the root for detection by confocal microscopy, (ii) uniform expression pattern, (iii) single insertion line (1 sensitive to 3 resistant segregation ratio) and, (iv) line with no obvious abnormal developmental phenotypes. Lines were rescreened in T3 using similar criteria as in T2 with the exception that we selected homozygous lines (100% resistant). At this step, we selected one transgenic line for each marker that was used for further analyses and crosses.

### Phenotyping of early root growth responses

Seeds were sowed in +Fe media described in Gruber *et al.*, 2013 (*63*) and stratified for 2–3 days at 4°C. Five days after planting, about 15 seedlings were transferred to a culture chamber (Lab-Tek, Chambers #1.0 Borosilicate Coverglass System, catalog number: 155361) filled with – Fe or + Fe medium described in Gruber *et al.*, 2013 (*63*) or + Fe medium containing flg22. Note that the transfer took about 45–60 seconds. Images were acquired every 5 minutes for 12 hours representing 145 images per root in brightfield conditions using a Keyence microscope model BZ-X810 with a BZ NIKON Objective Lens (2X) CFI Plan-Apo Lambda described as the Root Walker platform (*64*).

### Phenotyping of late root growth responses

Seeds were sowed in +Fe media described in Gruber *et al.*, 2013 (*63*) and stratified for 2–3 days at 4°C. Five days after planting, 6 plants per genotype were transferred to four 12 by 12 cm plates in a pattern in which the positions of the genotypes were alternating in a block design (top left, top right, bottom left and bottom right). After transfer, the plates were scanned every 24 hours for 3 days using the BRAT software.

### Microscopy setup

All imaging experiments except when indicated below, were performed with the following spinning disk confocal microscope set up: inverted Zeiss microscope equipped with a spinning disk module (CSU-X1, Yokogawa, https://www.yokogawa.com) and the prime 95B Scientific CMOS camera (https://www.photometrics.com) using a 40 or 63x Plan- Apochromat objective (numerical aperture 1.4, oil immersion). For colocalization, the LSM880 Airyscan was used. For Spt-PALM experiment, the Zeiss Elyra was used. GFP and mCITRINE, were excited with a 488 nm laser (150 mW) and fluorescence emission was filtered by a 525/50 nm BrightLine single-band bandpass filter. mCHERRY was excited with a 561 nm laser (80 mW) and fluorescence emission was filtered by a 609/54 nm BrightLine single-band bandpass filter (Semrock, http://www.semrock.com/). For FRET-FLIM analysis was performed with Leica stellaris 8 Falcon. For quantitative imaging, pictures of root cells were taken with detector settings optimized for low background and no pixel saturation. Care was taken to use similar confocal settings when comparing fluorescence intensity or for quantification.

### FRAP

Fluorescence in a rectangle region of interest (ROI) (50 μm2, 15 μm long) was bleached in the plasma membrane optical section by four successive scans at full laser power (150 W) using the FRAP module available on LSM880. FRAP was recorded continuously during 180 s with a delay of 5 s between frames. Fluorescence recovery was subsequently analyzed in the bleached ROIs and in controlled ROIs (rectangle with the same dimension in unbleached area). Fluorescence intensity of an ROI (same size as the bleached region) was systematically measured outside the root and subtracted to plasma membrane as background correction. Fluorescence intensity data were normalized as previously described (*65*). Briefly, fluorescence intensity data were normalized using the equation: In = [(It-Imin)/(Imax-Imin)]x100. Where In is the normalized intensity, It is the intensity at any time t, Imin is the minimum post-photobleaching intensity and Imax is the mean pre-photobleaching intensity.

### FRET-FLIM

FLIM experiments were carried out using a Leica Stellaris 8 FALCON confocal FLIM microscope using LAS-X software. The microscope was equipped with a humidified incubator with 5% CO2 at 37°C. Cells were excited with the 514 nm line of the white-light laser (Stellaris), and photon arrival times were recorded with one HyD detector, covering the mCITRINE emission spectrum (530/30 nm).

### TIRF microscopy

Total Internal Reflection Fluorescence (TIRF) Microscopy was conducted using the inverted ONI Nanoimager from Oxford microscope with a 100x Plan-Apochromat objective (numerical aperture 1.50, oil immersion). The optimum critical angle was determined as giving the best signal-to-noise ratio. Images were acquired with about 15% excitation (1 W laser power) and taking images every 100 ms for 300-time steps.

### Spt-PALM acquisition

Imaging was performed on a Zeiss Elyra PS1 system with a 100x Apo (numerical aperture 1.46 Oil objective), in TIRF mode. The optimum critical angle was determined as giving the best signal-to-noise ratio. Pixel size was 0.107 μm. mEOS was photoconverted using 405 nm UV laser power and resulting photoconverted fluorophores were excited using 561 nm laser. UV laser power was adjusted to obtain a significant number of tracks without an excessive density to facilitate further analyses (0.01 to 0.08%). 10,000 images time series were recorded at 25 frames per second (40 ms exposure time) on a 256 x 256-pixel region of interest. Single molecule detection and tracks reconstruction were made using MTT algorithm and further computational analyses of tracks were made using CBS spt-PALM analyzer. For more details (*34*). Care was taken to use similar settings when comparing molecules dynamics between different genotypes.

### Western blot

The protein extraction and Western blot procedures followed the previously published method with some modifications (*66*). About 100 seedlings of 5 days old light grown seedling on mesh of the respective transgenic lines were treated in +Fe, -Fe, or flg22[1] for 2 hours depending on the experiment. The roots were cut and immediately frozen in liquid nitrogen and then were ground with liquid nitrogen and lysed directly with 100 μL1xNuPAGE LDS Sample Buffer (Invitrogen, Cat. NP0008) supplemented with 1x NuPAGE Sample Reducing Agent (Invitrogen, Cat. NP0009) for 30 min on ice. The protein samples were denatured by heating for 10 min at 90°C and centrifuging at 11,000g for 10 min. The supernatant protein samples were separated by NUPAGE 10% Bis-Tris Plus Gel (Invitrogen, Cat.NW00105BOX) and transferred onto Nitrocellulose membrane using an iBlot 2 Dry Blotting system (Invitrogen, Cat. IB23001). The GFP signal was detected by Western blot with the corresponding antibody (α-GFP-HRP) (Cell Signaling, Cat. No. #4370) 1:2000 diluted in 1% BSA, Merck/Calbiochim, Cat. No.12657.

### Immunoprecipitation

Immunoprecipitation experiments to quantify SRF3 levels and ubiquitination required 1–3 g of root material from 12-day old seedlings grown on vertically oriented agar plates. Tissues were ground at 4°C and resuspended in 2 mL of ice- cold sucrose buffer (20 mM Tris at pH 8, 0.33 M sucrose, 1 mM EDTA at pH 8, protease and phosphatase inhibitors, 20 mM NEM DUB inhibitor). Samples were centrifuged at 5,000 x g for 10 min at 4°C until the supernatants were clear and tubes were changed in between each spin. The resulting extracts corresponded to the total protein fraction. Total protein was quantified, and equal amounts were loaded on an SDS-PAGE gel. For cellular fractionation, total protein fractions were centrifuged at 20,000 x g for 45 min at 4°C to pellet microsomes. The resulting supernatant fractions corresponded to the fraction enriched in soluble proteins. The pellet was resuspended in 1 mL of immunoprecipitation buffer (50 mM Tris at pH 8, 150 mM NaCl, 1% Triton X-100) using a 2-mL potter (Wheaton), and was left on a rotating wheel for 30 min at 4°C. Samples were then pelleted at 20,000 x g for 10 min at 4°C. The supernatant corresponded to the fraction enriched in microsomal-associated proteins. The proteins in each fraction were quantified, and immunoprecipitations were performed on 1 mg of proteins using a Milteneyi GFP kit and anti-P4D1 (1/2500) were used to detect general ubiquitination. For mass spectrometry analysis, about 1 g of root material from 7-day-old seedlings grown on vertically oriented agar plates were ground at 4°C and resuspended in 2 mL (50 mM Tris at pH 7.5, 150 mM NaCl, 5 mM EDTA at pH 7.5, Triton-X-100 at 1%, protease, and phosphatase inhibitors, 20 mM NEM DUB inhibitor). Samples were centrifuged at 5000 x g for 10 min at 4°C until the supernatants were clear. The resulting extracts corresponded to the total protein fraction. Similar procedures as indicated above were used for immunoprecipitation and detection of anti-GFP (1/2500) but using GFP-trap bead (chromotek).

### Mass spectrometry

Immunopurified proteins were reduced with dithiothreitol, alkylated with iodoacetamide and digested with trypsin overnight. Salts and reagents were removed by reversed phase (C-18) cartridge clean-up. The salt-free peptides were dissolved in 0.1% formic acid and subjected to ESI-MS/MS analysis on a Thermo LTQ-Orbitrap XL instrument. A capillary column (inner diameter 75 mm, packing length 10 cm of C-18 silica) with an integrated spray tip was used with a 300 nl min 1 0.1% formic acid/acetonitrile gradient. Peptide precursor masses were determined with high accuracy by Fourier transform MS in the Orbitrap followed by data-dependent MS/MS of the top five precursor ions in each chromatographic time window. Data were analysed using the Mascot algorithm (Matrix Science, London, UK on a local Mascot server (version 2.1.0)

### Iron treatments, flg20 and flg22 treatments

Seeds were sowed in +Fe medium described in Gruber *et al.*, 2013 (*63*) and stratified for 2–3 days at 4°C. 5-day-old seedlings were treated for 20 minutes or 2 hours with sufficient iron levels or low iron medium or for 20 minutes or 2 hours adding 100uM of FerroZine in liquid medium described in Gruber *et al.*, 2013 (*63*) using 12-well plates.

For flg22 treatment, Seeds were sowed in +Fe medium described in Gruber *et al.*, 2013 (*63*) and stratified for 2–3 days at 4°C. Five-day-old seedlings were treated for 20 minutes or 2 hours in iron-sufficient media described in Gruber *et al.*, 2013 (*63*) or low iron medium described in Gruber *et al.*, 2013 (*64*) supplemented with either, Na-Fe-EDTA, FeSO_4_ or FeCl_3_ at 75 μM in presence of flg22 or flg20 at 1 μM using 12-well plates. For iron cyanide salts, seeds were sowed in +Fe medium described in Gruber *et al.*, 2013 (*63*) and stratified for 2–3 days at 4°C (*19*). Five-day-old seedlings were treated for 30 minutes in ultra-pure water supplemented with different concentration of iron cyanide salts using 12-well plates. For treatment on *N. Benthamiana* leaves, two days after infiltration, leaves were cut in rectangles of about 0.5 cm^2^ and incubated in sufficient iron liquid media or low iron liquid media supplemented with 300 μM of ferrozine (for metabolomics). Leaves were then treated for the indicated time in a vacuum to optimize and standardize treatment infiltration.

### Chemical treatments with BFA, Wm, ES9–17, Dark, CHX and β-Estradiol

For BFA and ES9–17 treatments, transgenic lines were incubated in 12 well plates containing 25 μM of Brefeldin A or ES9–17 at 30 μM (BFA, Sigma, www.sigmaaldrich.com, BFA stock solution at 50 mM in DMSO, ES9–17 stock at 300 mM in EtOH) in the corresponding liquid Gruber medium (+Fe, -Fe, -Fe with Ferrozine at 100 μM, flg20 or flg22 at 1 μM) for 2 hours. For BFA washout, after 2 hours under BFA in standard condition the plants were transferred in standard condition without BFA for 1 hour. For cycloheximide at 100 μM (Sigma, www.sigmaaldrich.com, CHX) coupled with BFA at 50 μM the same experimental procedure was followed but the seedlings were pretreated for 2 hours prior to be submitted to mock, -Fe with Ferrozine at 100 μM, or flg22 at 1 μM treatments and kept in CHX/BFA. For Wortmannin treatment, transgenic lines were incubated in 12 well plates containing 30 μM Wortmannin (Sigma, www.sigmaaldrich.com, Wm stock solution at 30 mM in DMSO) in the corresponding liquid Gruber media (+Fe, -Fe, -FeFZ[100], flg20[1] or flg22[1]). For dark treatment, transgenic lines were placed in the corresponding liquid Gruber media (+Fe, -Fe, -Fe with Ferrozine at 100 μM, flg20 or flg22 at 1 μM) in 12 well plates and wrapped up in aluminum foil. For β-Estradiol treatment transgenic lines were placed in the corresponding agar Gruber media supplemented with β-Estradiol at 2.5 μM for 3 days. In all cases, roots were imaged within a 10-minute time frame window around the indicated time.

### Dye treatment with RhoNox-1, and DHE staining

Five-day-old seedlings were treated under the conditions indicated in the figure (mock or flg22) and then transferred to ultra-pure distilled water (Fisher Scientific Invitrogen UltraPure Distilled Water 500 mL Plastic Container–10977015) to get rid of any trace of iron from water supplemented with 2.5 μM of RhoNox-1 for 15 min (stock solution 5 mM; https:// goryochemical.com/) or with Dihydroethidium (DHE).

### ROS burst measurement

Eight leaf discs (4 mm in diameter) per individual genotype were collected in 96-well plates containing sterile water. After collection, leaf discs were recovered overnight. The next day, the water was replaced by a solution containing 17 μg/mL luminol (Sigma Aldrich), 20 μg/mL horseradish peroxidase (HRP, Sigma Aldrich) and the PAMP in the appropriate concentration. Luminescence was measured for the indicated period using a charge-coupled device camera (Photek Ltd., East Sussex UK).

### SRF3 Transcript Expression by RT-qPCR

Total mRNA was extracted from wild type (WT) and transgenic lines in Col-0 background such as: *pUBQ10::SRF3-mCitrine* (*SRF3^WT^*) and *pUBQ10::SRF3^5KR^-mCitrine* (*SRF3^5KR^*), using the Sigma-Aldrich Spectrum Plant Total RNA Kit. cDNA was produced using Applied Biosystems (by Thermo Fisher Scientific). High-Capacity cDNA Reverse Transcription Kit. The expression of SRF3 and the ubiquitous TCTP transcripts were tested by PCR or qPCR using the Bio-Rad CFX384 Real-Time System and Luna qPCR mix (New England Biolabs, M3003L) according to the manufacturer’s instructions using the following primers starting with “RT”: RT_SRF3_F, RT_SRF3_R, RT_TCTP_F and RT-TCTP_R.

### Iron concentration measurement

5-day old seedlings were grown under standard conditions (+Fe) then 10 plants were transferred in Gruber liquid media for 7 days with 10 μM of Na-Fe-EDTA, -Fe in 6 well plates. The total iron content was measured using a spectrophotometric method that was described previously (*67*). The seedlings were harvested and rinsed with deionized water five times and dried in an oven at 65°C for 2 days. After measuring the DW, the tissues were digested with 65% (v/v) HNO3 at 95°C for 6 hours, followed by adding 30% (v/v) H_2_O_2_ at 56°C for 2 hours. The iron content was analyzed in the assay solution (1 mM bathophenanthroline disulfonate (BPDS), 0.6 M sodium acetate and 0.48 M hydroxylamine hydrochloride). The FeCl_3_ solution was used to prepare the standard curve. The resulting Fe2 + -BPDS3 complex was measured with absorbance (535 nm) using a microplate reader. The iron content in each sample was calculated by the absorbance values against the standard curve and normalized by the DW.

### RNA-seq

Total RNA was extracted from roots of plants 5 days after germination using an RNA protein purification kit (Macherey-Nagel). Next-generation sequencing libraries were generated using the TruSeq Stranded mRNA library prep kits (Illumina, San Diego, CA, USA) and sequenced on a HiSeq 2500 instrument as single-end 50 base reads. Reads were mapped to the *Arabidopsis* genome (TAIR10) with TopHat2 (*68*, *69*). Read-count normalization and differential gene expression analysis were performed with the R package edgeR (v3.36.0) (*70*). TMM normalization was applied and per-gene logCPM values were computed using the cpm function with log = TRUE and prior.count = 1.

### Differentially expressed genes (DEGs)

For each genotype, treatment-vs-mock DEGs were identified using the edgeR pipeline used in Cao *et al.*, (*14*) code available at https://github.com/cm010713/immunity-iron-project, min_R_codes_for_manuscript.R) (*14*). Briefly, raw counts for the relevant six samples (three mock or three treated replicates) were loaded into a DGEList object; lowly expressed genes were filtered using rowSums(cpm(y) > 1) > = 2 (≥ 2 samples with CPM > 1); dispersions were estimated with *estimateGLMCommonDisp* and *estimateGLMTagwiseDisp*; a negative-binomial GLM was fit with glmFit; and the treatment-vs-mock contrast was tested with glmLRT. Genes with false-discovery rate (FDR) ≤ 0.05 (Benjamini–Hochberg) were considered differentially expressed; up- and down-regulated DEGs were assigned by the sign of the model log2 fold change. No additional |log2FC| cut-off was applied to the per-genotype DEG lists used in Fig. 5, I and J and table S2. Venn diagrams were drawn with the VIB online tool at http://bioinformatics.psb.ugent.be/webtools/Venn/; direction-specific overlap analyses were drawn with the VennDiagram R package.

### ANOVA-filtered gene set for clustering

The gene set used for clustering analysis (Fig. 6A and table S3) was obtained by ANOVA across all nine genotype × treatment groups in WT, *srf3*–*3*, and *SRF3*^5KR-comp^, retaining genes with Benjamini–Hochberg FDR < 0.05 and a maximum pairwise |log2 fold change| > 1 between any two condition groups. This yielded *n* = 7398 ANOVA-filtered genes.

### Clustering

ANOVA-filtered genes were row z-scored across all 27 RNA-seq samples and partitioned by k-means (kmeans, nstart = 50). Cluster-number diagnostics were computed using the Bayesian Information Criterion (BIC) formulation as used in Cao *et al.*, (*14*); R code at https://github.com/cm010713/immunity-iron-project) (*14*), defined as BIC = D + log(n)·m·k where D is the total within-cluster sum of squares, n the number of clustered genes (*n* = 7398), m the number of sample columns (m = 27), and k the number of k-means clusters. On the SRF3 dataset, k = 9 improved substantially over k = 5 (BIC_k = 5_ = 102,055; BIC_k = 9_ = 88,879) although the diagnostic did not uniquely select k = 9 because the curve continued to decrease at higher tested k values. The k = 9 solution was retained because it is the clustering solution used in the submitted table S3 and Fig. 6, and because it provided interpretable expression modules for the SRF3 dataset. Cluster-level heatmaps were drawn with the ComplexHeatmap package in R. GO enrichment analysis was conducted using the online tools at http://geneontology.org/.

### Aniline blue staining

Five-day old seedlings were incubated for 2 hours in mock or flg22 then transferred for 2 hours to 150 mM K2HPO4 and 0.01% aniline blue in 12-well plates wrapped in aluminum foil for light protection. Then imaging of the root epidermis in the transition/elongation zone was performed.

### CHA0 bacterial strain growth conditions and inoculation

The mCherry-labelled *P. protegens* (CHA0::attTn7-mCherry; Gmr) was used for the bacteria inoculation assay (*71*). The CHA0-mCherry strain was cultured in liquid LB medium (Miller’s LB Broth, Research products international, L24040) supplemented with 25 μg mL^−1^ gentamycin at 28°C overnight. Bacterial cells were collected by centrifugation (1 min, 5000 rpm) and resuspended in sterile MilliQ water five times to prevent potential element contamination from the LB medium. Analysis of the effect of CHA0 colonization on *Arabidopsis* roots was performed according to the previous studies with some modification (*71*–*73*). In brief, seedlings (aged 7 days) of Col-0 were transferred to 5 mL (Gruber medium without MES and sucrose) of +Fe (50 μM FeEDTA pH 5.5) Gruber liquid medium in a six-well plate and treated for 24 hours with prewashed CHA0-mCherry at a final OD600 of 0.02. After the treatment, the roots were gently washed with sterilized deionized water to remove the non-attached bacteria and the root length was measured. The roots were harvested in 1 mL extraction buffer (10 mM MgCl2, 0.01% Silwet L-77) and homogenized using Tissue Lyser with stainless steel beads. The samples were diluted serially from 10^1^ to 10^6^, and then spread onto LB agar plates supplemented with 30 μg mL^−1^ gentamycin. The colony-forming units (CFUs) were counted after 30 hours incubation at 28°C. The calculated CFUs were normalized to the root length.

### *R. solanacearum* growth conditions and pathogenicity assays

*R. solanacearum* strain GMI1000 bearing the constitutive luminescent reporter *PpsbA::LuxCDABE* (*74*) was used for all experiments. *R. solanacearum* was grown at 28°C in liquid rich B medium (0.1% yeast extract, 1% bacto peptone, and 0.1% casamino acids) overnight. For inoculation, the bacterial solution was adjusted with water to a final OD600 of 0.01 corresponding to 10^7^ colony-forming units (CFU) ml^−1^.

### In vitro inoculation and bacterial colonization assays

Seeds were surface sterilized with a solution containing 10% bleach in ethanol and sown in half MS media without sugar and 1% agar (Bacto Agar, Becton, Dickinson and Co.). Sown plates were stratified at 4°C in the dark for 2 days. Plates were then transferred to chambers and grown vertically for 7 days under constant conditions of 21–22°C, 60% humidity, and a 16 hours light/8 hours dark photoperiod. Seven-day old seedlings were transferred to inoculation plates containing 3 mL of the the bacterial solution or mock controls. Plates were transferred to a controlled growth chamber at 27°C, 60% humidity, and a 12 hours light/12 hours dark photoperiod for 5 days.

To quantify the bacterial growth inside the root, plant tissue was collected and placed in Eppendorf tubes filled with 1 mL of distilled water. Seedlings were surface sterilized with 1 mL 70% EtOH and rinsed three times with water. Plant material was dried using filter paper, placed inside the Eppendorf tubes and weighted. Distilled water was added to the Eppendorf and the material mechanically grinded. These suspensions were diluted using serial dilutions and plated to Rich B media plates supplemented with gentamycin. Colonies were counted 2 days after growing at 28°C and referred to the amount of material used (CFU/g material)

### Symptom recording assays

Pathogenicity tests were performed using the soil-drench method (*75*). Briefly, *Arabidopsis* plants were grown for 4 weeks in Jiffy pellets (Jiffy group, Jiffy Products International AS, Norway) at 22°C, 60% humidity and 8 hours light/16 hours dark photoperiod. Jiffys were cut at one-third from the bottom and were submerged for 30 min into a solution of overnight-grown *R. solanacearum* adjusted with distilled water to OD_600_ = 0.1 corresponding to 10^8^ colony-forming units (CFU) mL^−1^ (35 mL of bacterial solution per plant). Inoculated plants were transferred to trays containing a thin layer of soil drenched with the same *R. solanacearum* solution and kept in a chamber at 27°C, 60% humidity, and 12 hours light/12 hours dark. Plant wilting symptoms were recorded every day and expressed according to a disease index scale (0, no wilting; 1, 25% wilted leaves; 2, 50%; 3, 75%; and 4, death).

### Quantification and statistics

See Supplementary Materials.

## Supplementary Materials

### The PDF file includes:

Supplementary Text Figs. S1 to S8 Legends for tables S1 to S4 Tables S5 to S9 References
