# Supplementary material for: SRF3 couples cellular iron homeostasis and defense responses through ubiquitin-dependent nano-organization
Source: Sci Adv. 2026 Oct 14;12(42):eaeh4671. doi: 10.1126/sciadv.aeh4671 (PMC13666039; doi:10.1126/sciadv.aeh4671)
Supplement: Supplementary file 1 [file sciadv.aeh4671_sm.pdf]

Science Advances
AAAS

Supplementary Materials for

SRF3 couples cellular iron homeostasis and defense responses through ubiquitin-dependent nano-organization

Matthieu Pierre Platre *et al.*

Corresponding author: Matthieu Pierre Platre, matthieu.platre@inrae.fr; Wolfgang Busch, wbusch@salk.edu

Sci. Adv. **12**, eaeh4671 (2026)
DOI: 10.1126/sciadv.aeh4671

The PDF file includes:

Supplementary Text
Figs. S1 to S8
Legends for tables S1 to S4
Tables S5 to S9
References

Other Supplementary Material for this manuscript includes the following:

Tables S1 to S4

Supplementary Text

QUANTIFICATION METHOD

Late root growth response. Plates containing seedlings were scanned from days 5 to 9 after transfer to different media in order to acquire images for further quantification of the root growth rate per condition. Plates were scanned each day and were stacked together using a macro in Fiji (Macro_Match_Align). We then calculated the root length for every day per genotype in each condition to evaluate the root growth rate in Fiji using the segmented line. We first calculated the mean of the root growth rate for each day 5 to 6, 6 to 7 and 7 to 8. These values were used to calculate the mean of root growth rate for 3 days. Then, we divided the mean of root growth rate for 3 days to a given media for each plant by the mean of root growth rate for 3 days after transfer to the control media for the entire related genotype. This ratio was used as the late root growth response to low iron levels or flg22. Every experiment was repeated at least twice.

Early root growth response. Root length for each seedling was recorded for 12 hours taking a picture every 5 minutes and quantified using a Matlab® script ("Root Walker", *65*). From these measurements, we plotted the root length from T0 to T12 after transfer. We then obtained a curve representing the root length after transfer from which we calculated the area under the curve using the following formula "(Root length T1+Root length T2)/2*(Time T2-Time T1)". Then, we divided the value of the area under the curve after transfer for each plant in each condition by the area under the curve after transfer to the control media for the entire related genotype. This ratio was used as the early root growth response to low iron levels or flg22. Every experiment was repeated three times.

Fluorescence Integrated density. Images were acquired at the identical zone, elongation zone below the first identified emerged root hair. Then the root area was segmented and detected using the plugin "Wavelet a trou" (http://www.ens-lyon.fr/RDP/SiCE/METHODS). In this area, the integrated density was calculated. This process has been automated in a Macro (Macro_Integrated_Density). Every experiment was repeated three times.

Mean gray value at the plasma membrane. Confocal images were first denoised using an auto local threshold applying the Otsu method with a radius of 25 and a median filter with a radius of 2 in Fiji. To remove every single bright pixel on the generated-binary image the despeckle function was applied. To obtain a plasma membrane skeleton, we detected and removed every intracellular dot using the "Analyze Particles" plugin with the following parameter, size between 0.0001 and 35 000 µm2 and a circularity between 0.18 and 1. Then, we selected and cropped a zone which only showed a proper plasma membrane skeleton. We created a selection from the generated-plasma membrane skeleton and transposed it to the original image to calculate the plasma membrane intensity. This process has been automated in a Macro (Macro_PM/cyt_Intensity). An average of 45 cells were used for quantification per root. Every experiment was repeated three times.

Ratio of plasma membrane and cytosol intensity. Confocal images were first denoised using an auto local threshold applying the Otsu method with a radius of 25 and a median filter with a radius of 2 in Fiji. To remove every single bright pixel on the generated-binary image the

despeckle function was applied. To obtain plasma membrane skeleton, we detected and removed every intracellular dot using the “Analyze Particles” plugin with the following parameter, size between 0.0001 and 35 000 μm^2 and a circularity between 0.18 and 1. Then, we selected and cropped a zone which only showed a proper plasma membrane skeleton. We created a selection from the generated-plasma membrane skeleton and transposed it to the original image. First, we calculated the mean gray value of this zone, corresponding to the plasma membrane mean gray value. Then, on the original image, the same zone was removed from, and the mean gray value was calculated to obtain the cytosol mean gray value. The plasma membrane mean gray value was then divided by the cytosol mean gray value to normalize and to obtain the ratio of the plasma membrane and cytosol intensities. This process has been automated in a Macro (Macro_PM/Cyt_Intensity). An average of 45 cells were used for quantification per root. Every experiment was repeated three times.

Ratio of the root tip and differentiated zones intensity. The root tip containing the meristematic and elongation zones excluding the root cap was selected and the mean gray value was recorded using Fiji. Then, a zone corresponding to the same size area was selected in the differentiation zone and the mean gray value was recorded. The root mean gray value was divided by the differentiation zone mean gray value to obtain the ratio of the root tip and differentiated-zone intensity. Every experiment was repeated three times.

Ratio of the signal intensity of GFP, Tubulin and P4D1 on western blot. For calculating the signal intensity, the images were opened on Fiji and the gray images were inverted to obtain set the lowest value in black 0 and he highest in white 255 in 8 bit. Then the size of the ROI was determined based on the biggest band observed in the raw for a given antibody. Then the signal intensity of GFP was divided by the Tubulin or P4D1 by the GFP to obtain a normalized ratio depending on the protein quantity.

Plot profile intensity at the plasma membrane. The plot profile of the apical-basal plasma membrane was determined using the segmented line tool in the Fiji toolbox using a width of 4 pixels.

CLC2 cellular dynamics. Images were acquired at the same identical root developmental zone, meristem/transition zones since this is where CLC2 can be clearly visualized at the plasma membrane. To calculate the mean gray value, the root area was segmented and detected using the plugin “Wavelet a trou” (http://www.ens-lyon.fr/RDP/SiCE/METHODS). In this area, the mean gray value was calculated. For the spot size and the %Area, spots were detected and calculations generated using SiCESpotDetector.ijm plugin (http://www.enslyon.fr/RDP/SiCE/METHODS_files/SiCE%20SpotDetectorV3.ijm). To calculate the percentage of CLC2-GFP at plasma membrane (PM). Plasma membrane-positive CLC2-GFP were counted manually. The percentage of cells with CLC2 at the plasma membrane was obtained by counting the number of plasma membrane-positive CLC2 divided by the number of cells in the root meristematic zone multiplied by one hundred. Every experiment was repeated three times.

Colocalization analysis. For quantitative colocalization, we used an object-based analysis method (OBA). OBA is used to determine the centroid of each spot (intracellular compartment)

and to compare their respective localization. Co-localization between the two structures is validated if the distance between the two centroids is below the optical resolution. The OBA was performed as follows; first the intracellular compartments were automatically detected in each channel applying a ‘‘DoG’’ filter with a sigma of 3, and then a ‘‘Triangle’’ thresholding was applied. Next, with the binary images obtained we used JACoP plugin on Fiji to acquire quantitative data of the co-localization with the following parameters: minimum size of 3 mm2 and maximum size of 20 mm2. To allow high throughput data processing, this analysis pipeline was automated on a Fiji macro. The percentage of colocalization always corresponds to the proportion of spots in the yellow channel that colocalize with spots in the red channel. To avoid artifacts due to a low number of spots detected in one of the channels, a ratio was applied. This ratio corresponds to the number of spots detected in ‘‘Image A’’ divided by the number of spots detected in ‘‘Image B’’. If the ratio was either above 2 or below 0.5 the corresponding results were discarded. This process has been automated in a Macro (Macro_Coloc). Every experiment was repeated three times.

Density of diffraction limited spots. A homogeneous area at the plasma membrane on TIRF images acquired in a time-lapse image of about 10 seconds taking an image every 100 ns, to maximize the recording of the number of events occurring at the plasma membrane, were stacked together using the z-projection standard deviation. Then the image was converted to 8 bit and sharpened twice before being thresholded using Auto Threshold with the method Shanbhag. The corresponding image was then submitted to despeckle to remove small objects (<2 pixels). The polygon tool is used to select manually the ROI which corresponds to cell and the area surrounding the ROI is cleared using the Clear Outside function. The size of the Area is measured. Then the spots are detected using the “Analyze Particle” Plugin with a size of 2-35 pixels and a circularity of 0.8-1.0. Note that we selected a circularity which is pretty high to avoid counting moving molecules at the plasma membrane and focus our analysis on stable diffraction limited spots. From there, a mask image is generated and displayed every dot detected. The number of dots detected is then divided by the Area and multiplied by 100 to obtain the number of diffraction-limited spots. Every experiment was repeated three times.

Spt-PALM data quantifications and representations. For each movie corresponding to one condition/genotype, the natural logarithm of the apparent diffusion coefficient (log(D)) per molecule was extracted from the MTT data using the CBS sptPALM analyzer (*35*). For Spt-PALM analyses, only tracks with at least five successive points were selected and the rest of the molecules was discarded from the analysis. We pooled values from each condition to get the log(D) distribution using the R software. The set of molecules which present a log(D) value below -1.67 refers to the immobile group, whereas values above this threshold denote the mobile group. To obtain the percentage of molecules in the immobile group, we divided the number of tracks below -1.67 by the total track number multiplied by one hundred. Then, each condition was analyzed using an R script to automatize the analysis. To statistically test the existence of these two behaviors (mobile and immobile), we analyzed the log(D) distribution per condition using the mclust R package. For each condition, the percentages of data in each of the 2 groups defined by the partition P with the absolute log(D) value (-1.67) are first reported. This value was determined following a mixture model of scale mixtures of skew-normal denoted as FMSMSN. We then fitted two distinct two-component Gaussian mixture models, M0 and M1, using the mclust::Mclust R function. M0 is a model where $\sigma 1 \neq \sigma 2$ and M1 is a model where $\sigma 1 = \sigma 2$. For

each model, every molecule is assigned to one of the 2 Gaussian components Gi (i _[1, 2]) according to a MAP (Maximum A Posteriori)-based classification. M1 model was used since it provided more representative results despite a lower Bayesian information criterion values. The percentage of values assigned to each of the two Gaussian components is reported as well as its 95% confidence interval obtained with a non-parametric bootstrap method, using the mclust::MclustBootstrap R function with default arguments. The classification rate was calculated as the percentage of MAP-classified values concordant with the partition P. Based on the estimation of the model M1, we plotted the density of the Gaussian mixture according to the kernel density estimation (called "occurrence" for more clarity) and used it for representation. The R script can be found under "Script_mCLUST_R_SALK_DIFFUSION". At least 14 acquisitions from three independent experiments were used. In each graph relative to the percentage of immobile, "n" represents the number of acquisitions used for quantification. Note that one acquisition may represent more than one cell but that different acquisitions are always from different cells. For more details refer to (*35*).

LivePALM, Voronoi tessellation analyses and representation. For each movie corresponding to one condition/genotype, the object localization was extracted from MTT data by CBS sptPALM analyzer (*35*). SR-tesseler software was used to reconstruct live PALM images and determine protein local density (*38*). Multiple detections of single object were corrected as previously described by Levet et al. 2015 (*38*). Local density was extracted from Voronoï segmentation of the image as shown on Fig. 4d. We pooled values from each condition in order to get the log(local density) distribution using R software. A first partition P was done; the set of molecules which present a log(local density) value below 3 refers to the immobile group, whereas values above this threshold denote the mobile group. To obtain the percentage of molecules in the immobile group, we divided the number of molecules below 3 by the total molecules number multiplied by one hundred. Then, each condition was analyzed using an R script to automatize the analysis. To statistically test the existence of these two behaviors (mobile and immobile), we analyzed the local density distribution per condition using the mclust R package. For each condition, the percentages of data in each of the 2 groups defined by the partition P with the absolute log(local density) value (3) are first reported. We then fitted two distinct two-component Gaussian mixture models, M0 and M1, using the mclust::Mclust R function. M0 is a model where $\sigma 1 \neq \sigma 2$ and M1 is a model where $\sigma 1 = \sigma 2$. For each model, every molecule is assigned to one of the 2 Gaussian components Gi (i [1, 2]) according to a MAP (Maximum A Posteriori)-based classification. The M1 model was used since it provided more representative results despite a lower Bayesian information criterion values. The percentage of values assigned to each of the two Gaussian components is reported as well as its 95% confidence interval obtained with a non-parametric bootstrap method, using the mclust::MclustBootstrap R function with default arguments. The classification rate was calculated as the percentage of MAP-classified values concordant with the partition P. Based on the estimation of the model M1, we plotted the density of the Gaussian mixture according to the kernel density estimation (called "occurrence" for more clarity) and used it for representation. The R script can be found under "Script_mCLUST_R_SALK_LocalDensity". At least 24 cells from three independent experiments were used. In each graph relative to the fraction of clustered SRF3 in percentage, "n" represents the number of roots used for quantification.

Cluster density and size analysis. For each movie corresponding to one condition/genotype, the local density of each molecule was calculated with SR-Tesseler (*38*). For cluster detection, a threshold of 20 times the average localization density was applied to identify regions with high local density. "Objects" with at least 3 molecules were identified as "clusters" of molecules and from which the size of each cluster was extracted. n indicates the number of detected clusters. The R script for analysis and representation can be found under " Script_mCLUST_Cluster_diameter".

Quantification of aniline blue fluorescence intensity. Using Fiji, the integrated density signal of 10 plasma membranes in the apical-basal side of the epidermis in the elongation zone was calculated with the segmented line option with 3 pixels wide. These values were then pooled to calculate the average integrated density per root. In order to normalize the quantification, we evaluated the callose deposition response to flg22 based on the ratio of the aniline blue integrated density signal under flg22 divided by the mean of the aniline blue integrated density signal under mock conditions. These values were used to perform statistical analysis. This experiment was repeated twice.

STATISTICS & GRAPHS

Each experiment was repeated independently at least twice, as in every case the same trend was recorded for independent experiments and the data were pooled for further statistical analysis. Each sample was subjected to four different normality tests (Jarque-Bera, Lilliefors, Anderson-Darling and Shapiro-Wilk), samples were considered as a Gaussian distribution when at least one test was significant ($p<0.05$) using Xlstat.

- As a normal distribution was observed a one- or two-way ANOVA coupled with post hoc Tukey test was performed ($p<0.05$) using Xlstat.
- As a normal distribution was observed a one- or two-way ANOVA coupled with post hoc Fisher test was performed ($p<0.05$, $p<0.07$ and $p<0.1$) using Xlstat:
- As a normal distribution was observed an independent one- or two-way student's test was performed ($p=0.1$ or 0.05) using Xlstat.
- As a normal distribution was not observed a two-way Kruskal-Wallis coupled with post hoc Steel-Dwass-Critchlow-Fligner procedure was performed ($p<0.05$) using Xlstat.

The type of test performed is indicated in the figure legend.
In each graph, according to the box plot representation it depicts, the minimum as the lowest data point in the data set excluding any outliers, the maximum as the highest data point in the data set excluding any outliers, the median as the mid value in the data set, the first quartile as the median of the lower half of the data set, and the third quartile as the median of the upper half of the data set. The cross represents the mean of the data set. Circles represent each individual measurements in the data set.

CLONING

SRF3 mutagenesis.

SRF3 mutant impaired for ubiquitination was obtained by phosphorylated primers or site-directed mutagenesis using SRF3-cds_p221 as a template. 5' phosphorylated primers such as SRF3_cDNA_K391392R_p_F and SRF3_cDNA_K391392R_p_R were used to mutate lysines (K) 391 and 392 into arginine (R). Then, lysines (K) 444, 458, 471 were mutated into arginine (R) by site-directed mutagenesis using the following primers. SRF3_K444R_F, SRF3_K444R_R, SRF3_K458R_F, SRF3_K458R_R, SRF3_K471R_F and SRF3_K471R_R.

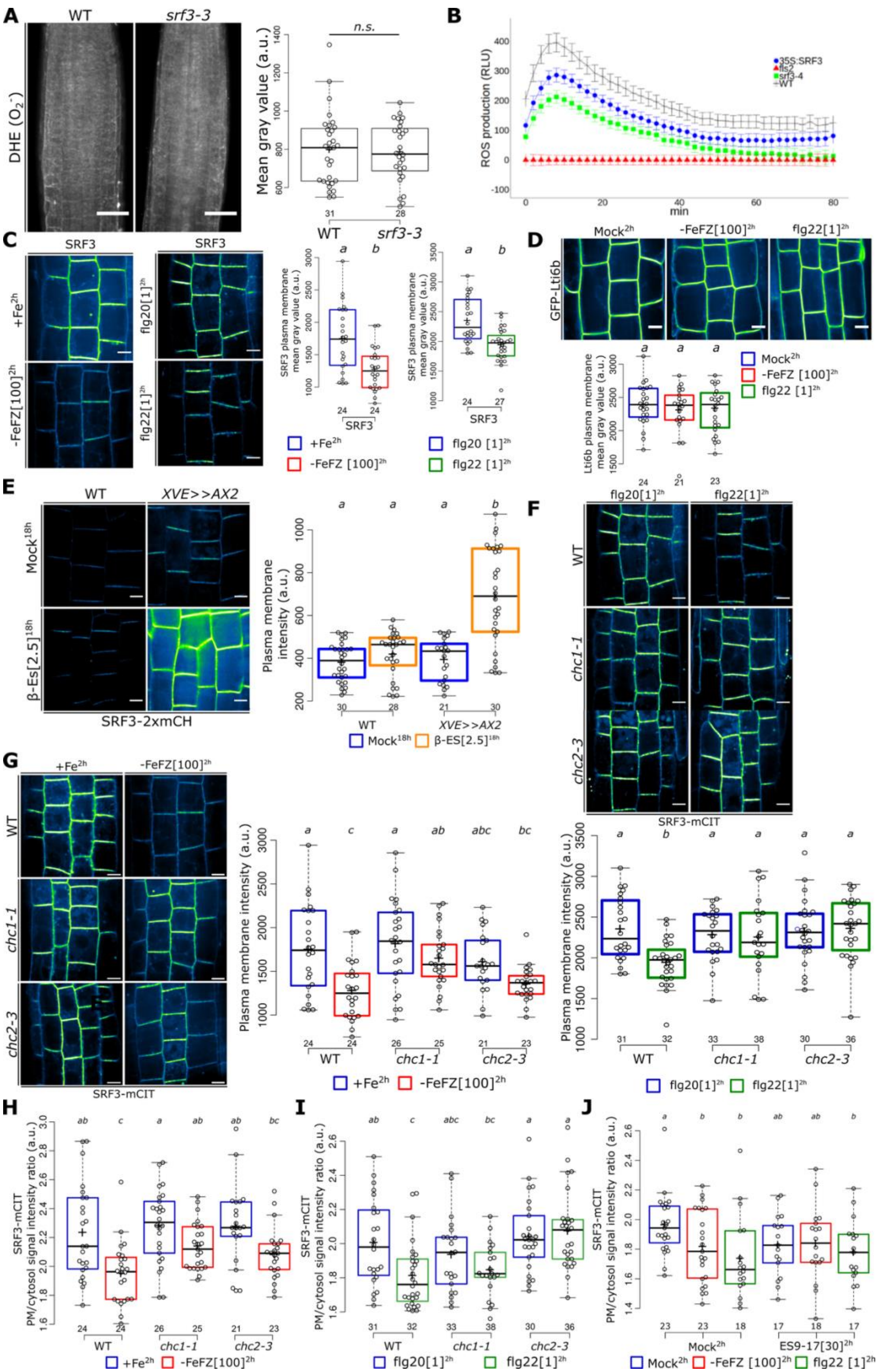
A
WT
srf3-3
DHE (O2-)
Mean gray value (a.u.)
n.s.
31
28
WT
srf3-3
B
ROS production (RLU)
min
35S:SRF3
fls2
srf3-4
WT
C
SRF3
+Fe2h
-FeFZ[100]2h
flg20[1]2h
flg22[1]2h
SRF3 plasma membrane mean gray value (a.u.)
a
b
24
24
27
SRF3
+Fe2h
-FeFZ [100]2h
flg20 [1]2h
flg22 [1]2h
D
Mock2h
-FeFZ[100]2h
flg22[1]2h
GFP-Lti6b
Lti6b plasma membrane mean gray value (a.u.)
Mock2h
-FeFZ [100]2h
flg22 [1]2h
24
21
23
E
WT
XVE>>AX2
Mock18h
β-Es[2.5]18h
SRF3-2xmCH
Plasma membrane intensity (a.u.)
30
28
21
30
Mock18h
β-ES[2.5]18h
F
flg20[1]2h
flg22[1]2h
WT
chc1-1
chc2-3
SRF3-mCIT
Plasma membrane intensity (a.u.)
31
32
33
38
30
36
flg20[1]2h
flg22[1]2h
G
+Fe2h
-FeFZ[100]2h
WT
chc1-1
chc2-3
SRF3-mCIT
Plasma membrane intensity (a.u.)
a
c
a
ab
abc
bc
24
24
26
25
21
23
+Fe2h
-FeFZ[100]2h
H
SRF3-mCIT
PM/cytosol signal intensity ratio (a.u.)
ab
c
a
ab
ab
bc
24
24
26
25
21
23
WT
chc1-1
chc2-3
+Fe2h
-FeFZ[100]2h
I
ab
c
abc
bc
a
a
31
32
33
38
30
36
flg20[1]2h
flg22[1]2h
J
a
b
b
ab
ab
b
23
23
18
17
18
17
Mock2h
ES9-17[30]2h
Mock2h
-FeFZ [100]2h
flg22 [1]2h

Figure S1: SRF3 is removed from the plasma membrane by clathrin-dependent endocytosis but does not regulate the ROS burst under flg22.

(A) Confocal images of 5-day-old seedlings of WT or *srf3-3* stained with DHE and the related quantification, right [two-way Student's test ($p < 0.05$)]. Scale bars 50 µm.

(B) Leaf discs from fully expanded leaves of 5-week-old WT, *srf3-4*, *SRF3-OX* and *fls2* plants were treated with flg22 (1 µM) to elicit ROS. Values represent the mean ±SE from at least 12 replicates per treatment and are expressed in photon counts [relative light units (RLU)].

(C) Confocal images of 5-day-old seedlings expressing SRF3-mCIT, under mock or -Fe supplemented with 100µM of ferrozine (-FeFZ[100]), and flg20 or flg22 at 1µM (flg20[1] and flg22[1]) for 2 hours and the related quantification, right [two-way ANOVA, Tukey HSD test, letters indicate statistical differences ($p < 0.05$)]. Scale bars 10µm.

(D) Confocal images of 5-day-old seedlings expressing *p35s::GFP-Lti6b*, under mock or -Fe supplemented with 100µM of ferrozine (-FeFZ[100]), and flg22 at 1µM (flg22[1]) for 2 hours and the related quantification, right [two-way ANOVA, Tukey HSD test, letters indicate statistical differences ($p < 0.05$)]. Scale bars, 10µm.

(E) Left panel: confocal images of root epidermal cells of 5-day-old seedlings expressing *pUBQ10::SRF3-2xmCHERRY* or co-expressing *pUBQ10::SRF3-2xmCHERRY* (SRF3-2xmCH) and *pUBQ10-XVE::AUXILIN-LIKE1 (XVE>>AX2)* under mock treatment or under β-Estradiol at 2.5µm (β-ES[2.5]) for 18 hours. Right panel: quantification of SRF3 plasma membrane mean gray value (a.u.). [two-way ANOVA followed by a post hoc Tukey HSD test; letters indicate statistical differences ($p<0.05$)]. Scale bars, 10µm.

(F) Upper panel: confocal images of root epidermal cells of 5-day-old seedlings expressing *pUBQ10::SRF3-mCITRINE* (SRF3-mCIT) in WT, *chc1-1* or *chc2-3* mutants treated with +Fe or -Fe supplemented with Ferrozine (-FeFZ[100]) at 100 µM for 2 hours. Lower panel: quantification of SRF3 plasma membrane mean gray value (a.u.). [two-way ANOVA followed by a post hoc Tukey HSD test; letters indicate statistical differences ($p<0.05$)]. Scale bar, 10µm.

(G) Left panel: confocal images of root epidermal cells of 5-day-old seedlings expressing *pUBQ10::SRF3-mCITRINE* (SRF3-mCIT) in WT, *chc1-1* or *chc2-3* mutants treated with flg20 or flg22 at 1 µM for 2 hours. Right panel: quantification of SRF3 plasma membrane mean gray value (a.u.). [two-way ANOVA followed by a post hoc Tukey HSD test; letters indicate statistical differences ($p<0.05$)]. Scale bar, 10µm.

(H) Quantification of the plasma membrane to cytosol signal intensity ratio of *pUBQ10::SRF3-mCITRINE* (SRF3-mCIT) in WT, *chc1-1* or *chc2-3* mutants treated with +Fe or -Fe supplemented with 100 µM of Ferrozine (-FeFZ[100]) at 100 µM for 2 hours. [two-way ANOVA followed by a post hoc Tukey HSD test; letters indicate statistical differences ($p<0.05$)].

(I) Quantification of the plasma membrane to cytosol signal intensity ratio of *pUBQ10::SRF3-mCITRINE* (SRF3-mCIT) in WT, *chc1-1* or *chc2-3* treated with flg20 or flg22 at 1 µM for 2 hours. [two-way ANOVA followed by a post hoc Tukey HSD test; letters indicate statistical differences ($p<0.05$)]. In all graphs, the black circles represent the count of individual measurements taken from a single image of one root over three independent experiments.

(J) Quantification of the plasma membrane to cytosol signal intensity ratio of *pUBQ10::SRF3-mCITRINE* (SRF3-mCIT), under mock or treated with ES9-17 at 30µM with +Fe, -Fe supplemented with 100 µM of ferrozine (-FeFZ[100]), and flg22 at 1µM (flg22[1]) for 2 hours. [two-way ANOVA followed by a post hoc Tukey HSD test; letters indicate statistical differences ($p<0.05$)]. In all graphs the numbers shown below the box plots represent the count of individual

measurements taken from a single image of one root, pooled across three independent experiments. Note that the images and the corresponding quantifications under flg20[1], flg22[1], + Fe -FeFZ[100] treatments are identical in panels (C), (F) and (G) as experiments were performed concurrently.

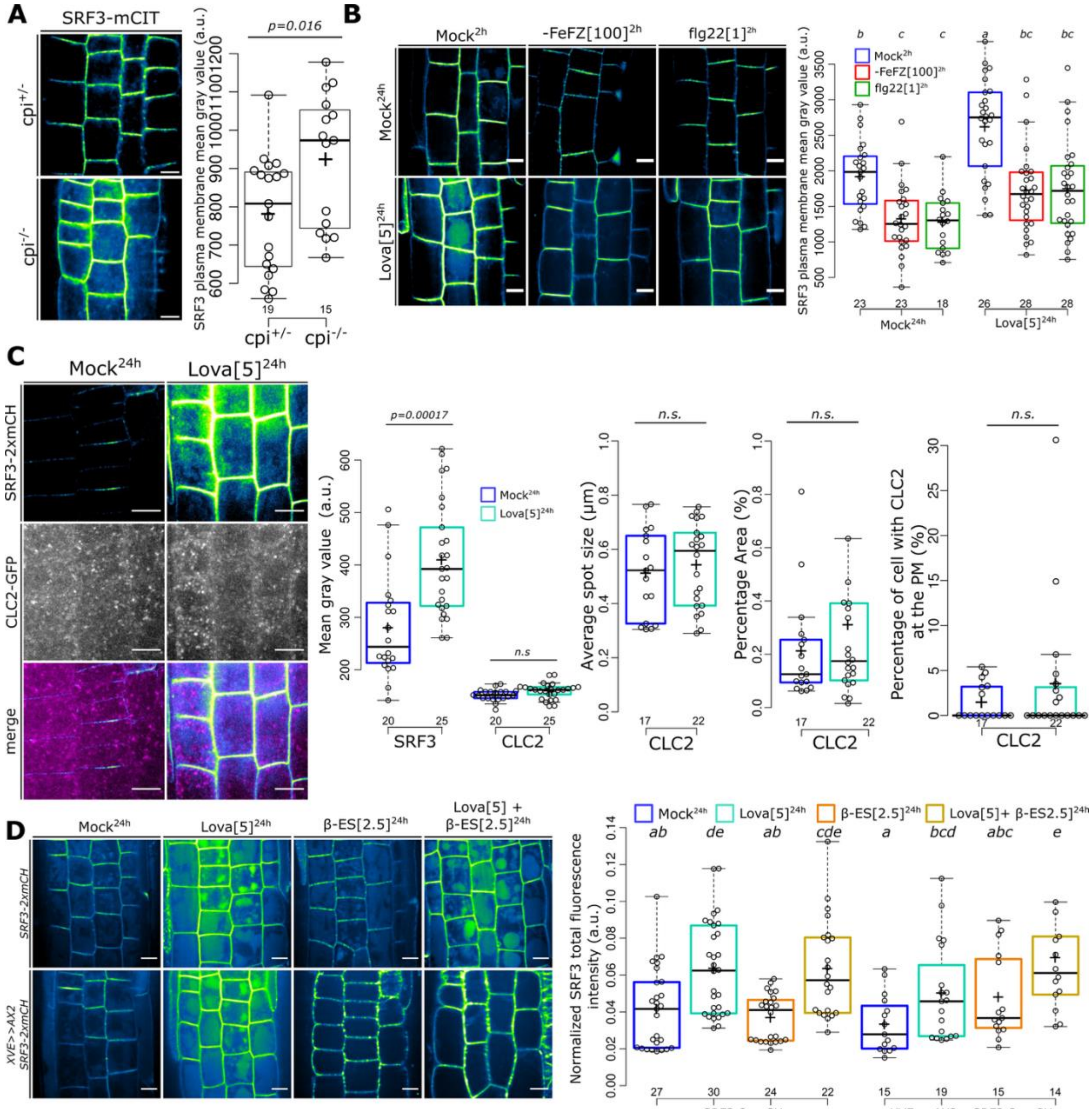

Figure S2: Clathrin-independent endocytosis controls SRF3 levels at the plasma membrane in standard condition but not upon flg22 or low ferrous iron levels.

(A) Confocal images of root epidermal cells of 5-day-old seedlings expressing *pUBQ10::SRF3-mCITRINE* (SRF3-mCIT) in cpi$^{-/+}$ or cpi$^{-/-}$ mutants in standard conditions and the related quantification of SRF3 plasma membrane mean gray value (a.u.). [two-way student's test ($p<0.05$)]. Scale bars, 10 µm.

(B) Confocal images of root epidermal cells of 5-day-old seedlings expressing *pUBQ10::SRF3-mCITRINE* (SRF3-mCIT) pre-treated with or without lovastatin (LOVA) at 5 µM for 24 hours with +Fe or -Fe supplemented with Ferrozine (-FeFZ[100]) at 100 µM or flg22 at 1 µM for 2 hours and the related quantification of SRF3 plasma membrane mean gray value (a.u.). [two-way ANOVA followed by a post hoc Tukey HSD test; letters indicate statistical differences

($p<0.05$)]. Scale bars, 10 µm. Note that the images and the corresponding quantifications under mock treatment are identical to Figure 2D, as both experiments were performed concurrently.
(C) Confocal images of root epidermal cells of 5-day-old seedlings co-expressing *pUBQ10::SRF3-mCHERRY-4xmyc* (SRF3-2xmCHERRY) with *pCLC2::CLC2-GFP* treated with or without Lovastatin (LOVA) for 24 hours and the related quantification of SRF3 and CLC2 total mean gray value (a.u.), average spot size, percentage area (%), and percentage of cells with CCL2 at the PM (%). [two-way student's test ($p<0.05$)]. Scale bars, 10 µm.
(D) Left panel: confocal images of root epidermal cells of 5-day-old seedlings expressing *pUBQ10::SRF3-2xmCHERRY* or co-expressing *pUBQ10::SRF3-2xmCHERRY* (SRF3-2xmCH) and *pUBQ10-XVE::AUXILIN-LIKE1 (XVE>>AX2)* under mock treatment or under Lovastatin (LOVA) at 5µM and/or β-Estradiol at 2.5 µm (β-ES[2.5]) for 24 hours and the related quantification of SRF3 normalized fluorescence intensity (a.u.). [two-way ANOVA followed by a post hoc Tukey HSD test; letters indicate statistical differences ($p<0.05$)]. Scale bars, 10 µm.
In all graphs the numbers shown below the box plots represent the count of individual measurements taken from a single image of one root, pooled across three independent experiments.

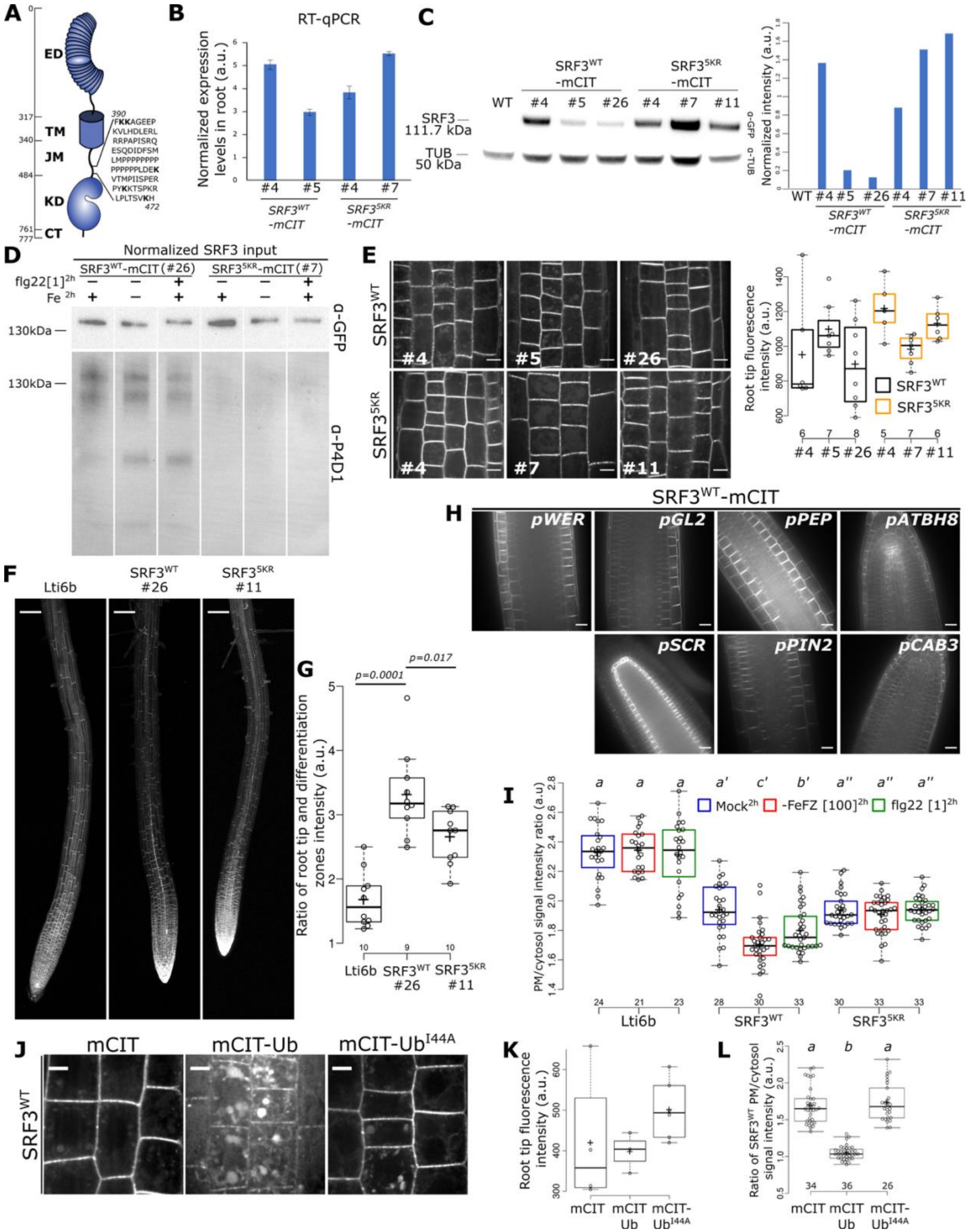

Figure S3: Characterization of SRF3^{WT}, SRF3^{5KR} and artificially ubiquitinated lines.
(A) Schematic representation of SRF3 ubiquitination sites.

(B) RT-qPCR using SRF3 primers in two different insertion lines of *pUBQ10::SRF3^WT^-mCITRINE* ($SRF3^{WT}$), *pUBQ10::SRF3^5KR^-mCITRINE* ($SRF3^{5KR}$) of root material from 5-day-old seedlings. Data include 3 technical repeats from 1 biological replicates. Experiment was repeated twice. SRF3 expression levels was normalized to the housekeeping gene TCTP. Error bars represent the standard deviation of the mean.
(C) Western blot on root material of three different insertion lines of *pUBQ10::SRF3^WT^-mCITRINE* ($SRF3^{WT}$), *pUBQ10::SRF3^5KR^-mCITRINE* ($SRF3^{5KR}$) of 7-day-old seedlings using anti-GFP and anti-TUB and the related quantification normalized to tubulin.
(D) Co-immunoprecipitation (Co-IP) using anti-GFP on root material from 12-day-old seedlings expressing $SRF3^{WT}$-mCIT or $SRF3^{5KR}$-mCIT in standard or treated conditions with -Fe and flg22 at 1µM (flg22[1]) for 2 hours. The input has been normalized to obtain the same protein quantity and ubiquitination is detected using anti-P4D1.
(E) Left panel: confocal images of root epidermal cells of 7-day-old seedlings expressing *pUBQ10::SRF3^WT^-mCITRINE* ($SRF3^{WT}$), *pUBQ10::SRF3^5KR^-mCITRINE* ($SRF3^{5KR}$) in three different insertion lines. Right panel: quantification of the fluorescence in the root tip. Scale bars, 5 µm. Numbers indicate the name of the independent transgenic insertion lines. Independent two-way Student's test ($p < 0.05$)].
(F-G) Confocal images of roots of 7-day-old seedlings expressing *p35s::GFP-Lti6b* (*Lti6b*) or *pUBQ10::SRF3^WT^-mCITRINE* ($SRF3^{WT}$) or *pUBQ10::SRF3^5KR^-mCITRINE* ($SRF3^{5KR}$, F). Quantification of the ratio of the root tip and differentiation zone intensity (a.u., right, G). Scale bars, 50 µm.
(H) Confocal images of root tips of 7-day-old seedlings expressing *SRF3^WT^-mCITRINE* driven by the indicated promoter. Scale bars, 10 µm.
(I) Quantification of the plasma membrane to cytosol signal intensity ratio of 35s::GFP-Lti6b (Lti6b), *pUBQ10::SRF3^WT^-mCITRINE* ($SRF3^{WT}$), *pUBQ10::SRF3^5KR^-mCITRINE* ($SRF3^{5KR}$) treated with +Fe or -Fe supplemented with Ferrozine (-FeFZ[100]) at 100 µM) flg22 at 1µM (flg22[1]) for 2 hours. [two-way ANOVA followed by a post hoc Tukey HSD test; letters indicate statistical differences ($p<0.05$)].
(J-L) Confocal images of 5-day-old seedlings expressing $SRF3^{WT}$-mCIT, $SRF3\text{-}mCIT^{Ub}$ and $SRF3\text{-}mCIT^{Ub\text{-}I44A}$ (I) and the related quantification of the fluorescence in the root tip (J) and of the plasma membrane to cytosol signal intensity ratio, (K) [one-way ANOVA, Tukey HSD test, letters indicate statistical differences ($p < 0.05$)]. Red arrows depict vacuoles. Scale bars, 10 µm. In all graphs the numbers shown below the box plots represent the count of individual measurements taken from a single image of one root, pooled across three independent experiments.

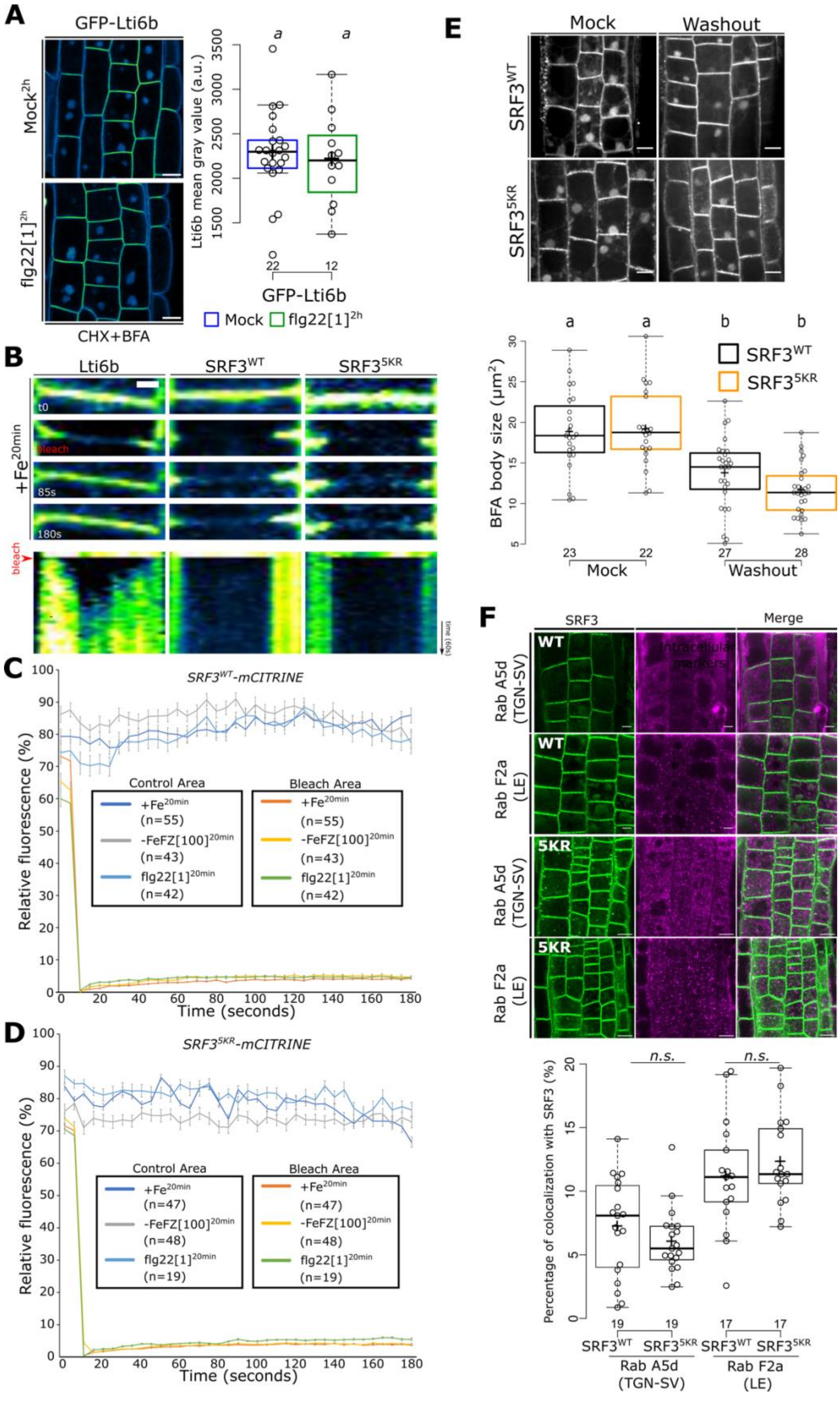

A
GFP-Lti6b
Mock2h
flg22[1]2h
CHX+BFA
Lti6b mean gray value (a.u.)
a
a
22
12
GFP-Lti6b
Mock
flg22[1]2h
B
Lti6b
SRF3WT
SRF35KR
+Fe20min
t0
bleach
85s
180s
C
SRF3WT-mCITRINE
Relative fluorescence (%)
Time (seconds)
Control Area
+Fe20min (n=55)
-FeFZ[100]20min (n=43)
flg22[1]20min (n=42)
Bleach Area
+Fe20min (n=55)
-FeFZ[100]20min (n=43)
flg22[1]20min (n=42)
D
SRF35KR-mCITRINE
Relative fluorescence (%)
Time (seconds)
Control Area
+Fe20min (n=47)
-FeFZ[100]20min (n=48)
flg22[1]20min (n=19)
Bleach Area
+Fe20min (n=47)
-FeFZ[100]20min (n=48)
flg22[1]20min (n=19)
E
Mock
Washout
SRF3WT
SRF35KR
a
a
b
b
SRF3WT
SRF35KR
BFA body size (µm2)
23
22
27
28
Mock
Washout
F
SRF3
Intracellular markers
Merge
WT
Rab A5d (TGN-SV)
WT
Rab F2a (LE)
5KR
Rab A5d (TGN-SV)
5KR
Rab F2a (LE)
n.s.
n.s.
Percentage of colocalization with SRF3 (%)
19
19
17
17
SRF3WT
SRF35KR
SRF3WT
SRF35KR
Rab A5d (TGN-SV)
Rab F2a (LE)

Figure S4: The ubiquitination of the five lysine residues does not play a major role in SRF3 recycling and intracellular trafficking.
(A) Confocal images of 5-day-old seedlings expressing *p35s::GFP-Lti6b*, under cotreatment with CHX 100µM and BFA 25µM alone or supplemented with flg22 at 1 µM (flg22[1]) for 2 hours and the related quantification, right. [two-way ANOVA, Tukey HSD test, letters indicate statistical differences ($p < 0.05$)]. Scale bars, 10 µm.
(B-D) Confocal images of 5-day-old seedlings expressing *pUBQ10::SRF3^{WT}-mCITRINE* (SRF3^{WT}), *pUBQ10::SRF3^{5KR}-mCITRINE* (SRF3^{5KR}) and *p35s::GFP-Lti6b* during FRAP experiment in wild type, and the related kymograph (time scale 60 seconds, B). The related traces of SRF3^{WT} (C) or SRF3^{5KR} (D) fluorescence intensity at the plasma membrane during FRAP analyses in +Fe or -Fe supplemented with Ferrozine (-FeFZ[100]) at 100 µM or flg22 at 1 µM for 20 minutes.
(E) Top panel: confocal images of 5-day-old seedlings expressing *pUBQ10::SRF3^{WT}-mCITRINE* (SRF3^{WT}), *pUBQ10::SRF3^{5KR}-mCITRINE* (SRF3^{5KR}) treated with BFA at 25 µM for 2 hours or washout during one hour and, bottom panel, the related quantification of the BFA body size [two-way ANOVA, Tukey HSD test, letters indicate statistical differences ($p < 0.05$)]. Scale bars, 10 µm.
(F) Top panel: confocal images of 5-day-old seedlings expressing *pUBQ10::SRF3^{WT}-mCITRINE* (SRF3^{WT}), *pUBQ10::SRF3^{5KR}-mCITRINE* (SRF3^{5KR}) with the indicated endosomal markers and, lower panel, the related quantification of the intracellular colocalization (%). [Independent two-way Student's test ($p<0.05$)]. Scale bars, 10 µm.
In all graphs the numbers shown below the box plots represent the count of individual measurements taken from a single image of one root, pooled across three independent experiments.

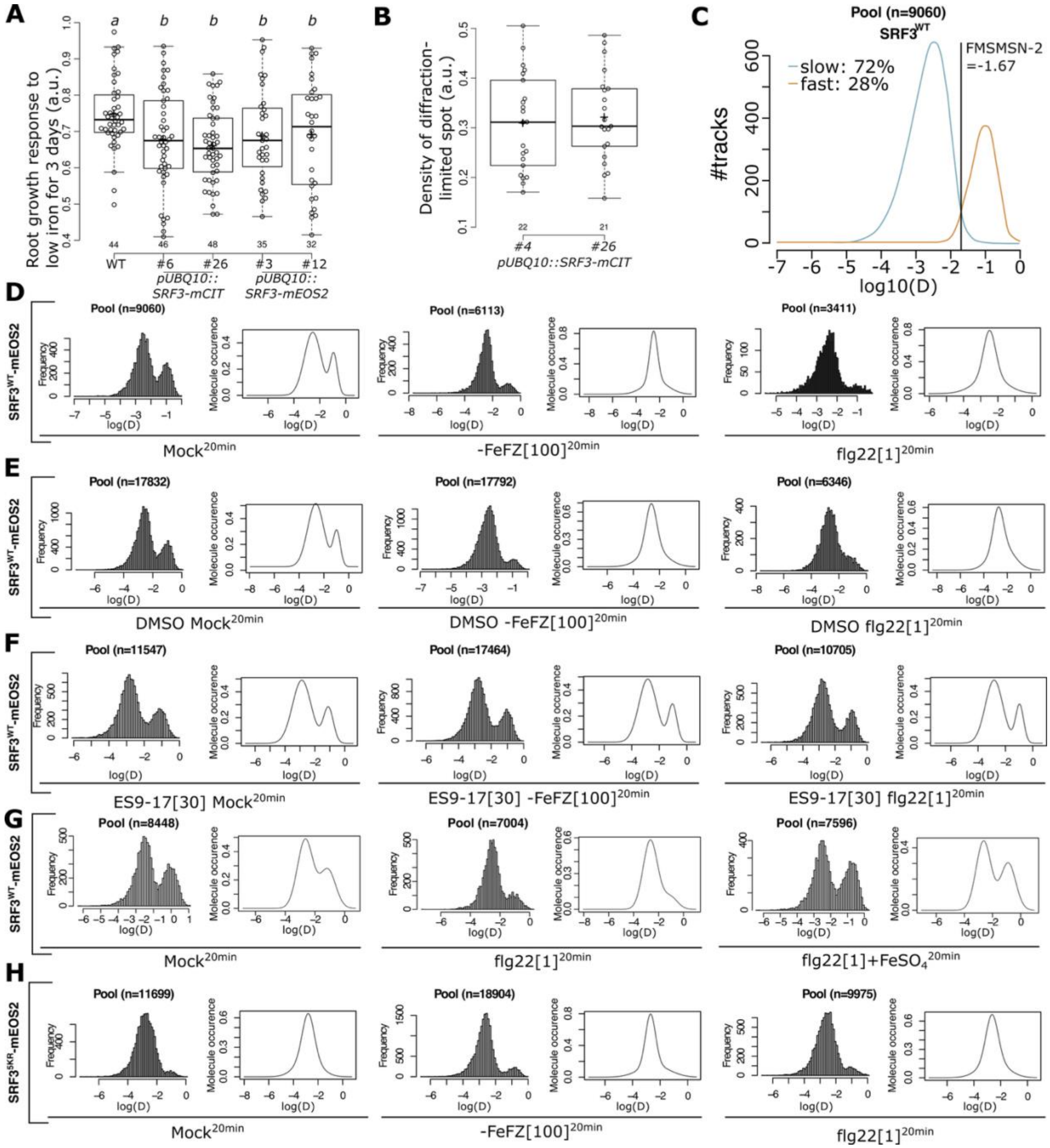

Figure S5: SRF3 segregates into two different populations at the plasma membrane in mobile and immobile fractions in a ubiquitin and ferrous iron dependent manner.

(A) Quantification of the root growth of 8-day-old seedlings of WT, *pUBQ10::SRF3-mCITRINE* and *pUBQ10::SRF3-mEOS2* grown under iron sufficient media (+Fe) conditions for 5 days and then transferred for 3 days to iron-sufficient media (+Fe) or to low iron medium supplemented with Ferrozine at 100 µM (–FeFZ[100]). Lower panel: the late root growth response. Scale bars, 1 cm [two-way ANOVA, Fisher (LSD) test, letters indicate statistical differences ($p < 0.05$)]. Numbers indicate the name of the independent transgenic insertion lines. In the graph the

numbers shown below the box plots represent the count of individual measurements taken from a single image of one root, pooled across three independent experiments.

(B) Quantification of the density of diffraction-limited spots in two independent lines of 5-day-old seedling expressing *pUBQ10::SRF3-mCITRINE.*

(C) Distribution of *pUBQ10::SRF3^{WT}-mEOS2* (SRF3^{WT}-mEOS2) molecules according to their apparent diffusion coefficient D obtained by analyzing Spt-PALM trajectories under mock conditions and separated in two fractions according to a mixture model of scale mixtures of skew-normal.

(D-H) Left panels: representation of the frequency of SRF3^{WT}-mEOS2 molecules according to their apparent diffusion coefficient (log(D) obtained by analyzing Spt-PALM trajectories treated with +Fe or -Fe supplemented with Ferrozine (-FeFZ[100]) at 100 µM or flg22 at 1 µM in the wild-type background (D) or in similar condition with addition of DMSO (D) or ES9-17 at 30 µM (E) or flg22 supplemented with 75 µM of $FeSO_4$ at 1 µM (F) or in SRF3^{5KR}-mEos2 background for 20 minutes (G). Right panels: correspond to identical frequency analysis but processed by the R mClust package. “n” corresponds to the number of trajectories analyzed.

In all graphs the numbers shown below the box plots represent the count of individual measurements taken from a single image of one root, pooled across three independent experiments.

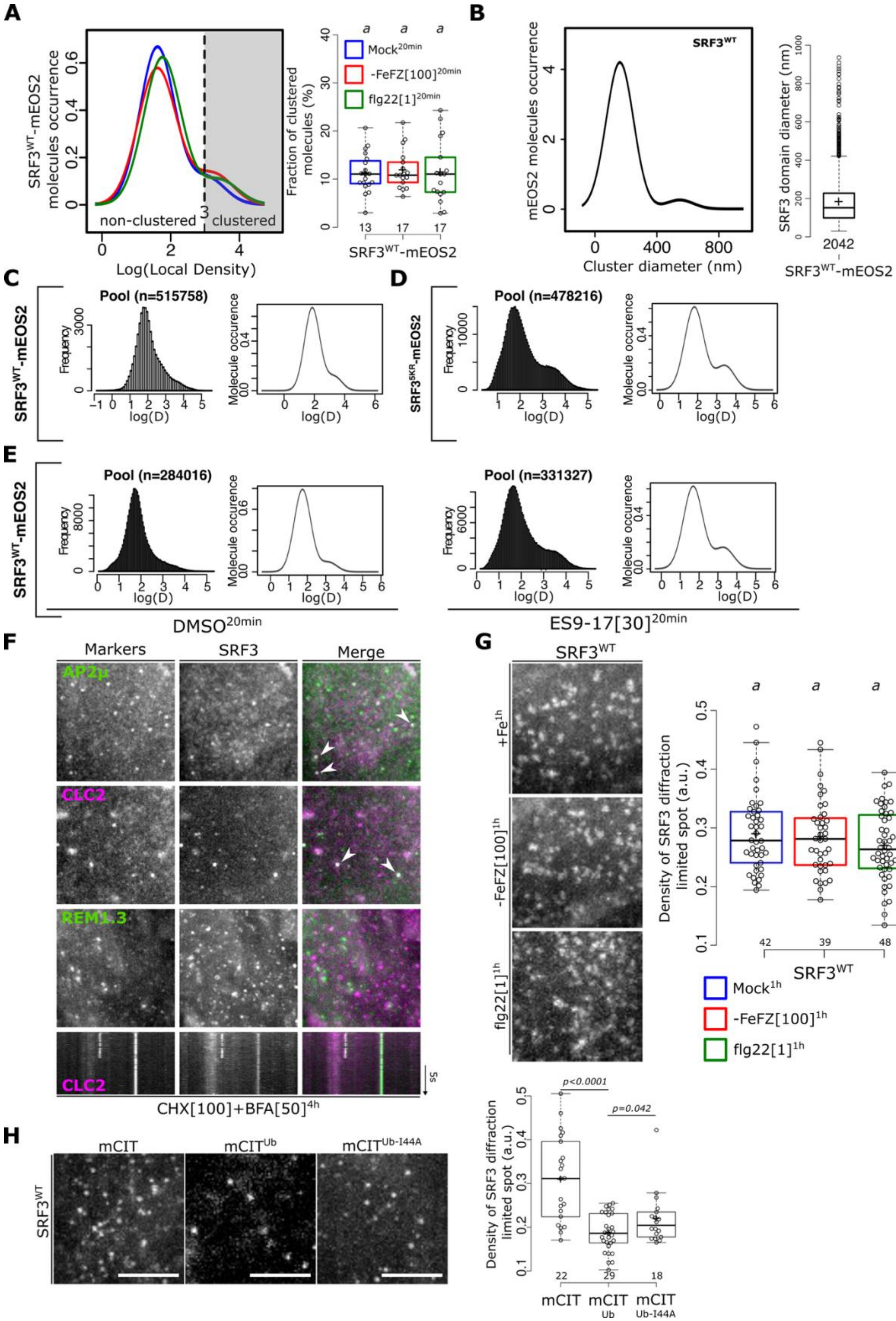

A
SRF3WT-mEOS2 molecules occurrence
non-clustered
clustered
Log(Local Density)
Fraction of clustered molecules (%)
Mock20min
-FeFZ[100]20min
flg22[1]20min
a a a
13 17 17
SRF3WT-mEOS2
B
SRF3WT
mEOS2 molecules occurrence
Cluster diameter (nm)
SRF3 domain diameter (nm)
2042
SRF3WT-mEOS2
C
SRF3WT-mEOS2
Pool (n=515758)
Frequency
log(D)
Molecule occurence
D
SRF35KR-mEOS2
Pool (n=478216)
E
SRF3WT-mEOS2
Pool (n=284016)
Pool (n=331327)
DMSO20min
ES9-17[30]20min
F
Markers
SRF3
Merge
AP2µ
CLC2
REM1.3
CLC2
5s
CHX[100]+BFA[50]4h
G
SRF3WT
+Fe1h
-FeFZ[100]1h
flg22[1]1h
Density of SRF3 diffraction limited spot (a.u.)
a a a
42 39 48
SRF3WT
Mock1h
-FeFZ[100]1h
flg22[1]1h
H
mCIT
mCITUb
mCITUb-I44A
SRF3WT
Density of SRF3 diffraction limited spot (a.u.)
p<0.0001
p=0.042
22 29 18
mCIT
mCIT Ub
mCIT Ub-I44A

Figure S6: SRF3 ubiquitin-dependent endocytosis occurs in immobile nanoclusters.
(A) Live PALM analysis of *pUBQ10::SRF3^{WT}-mEOS2* (SRF3^{WT}-mEOS2) molecules density by tessellation-based automatic segmentation of super resolution images to determine the cluster density under mock, -Fe supplemented with Ferrozine (-FeFZ[100]) at 100 µM or flg22 at 1 µM for 20 minutes and the related quantification of the cluster density [two-way ANOVA, Tukey (HSD) test, letters indicate statistical differences ($p < 0.05$)].
(B) Left panel: distribution of *pUBQ10::SRF3^{WT}-mEOS2* (SRF3^{WT}-mEOS2) molecules according to cluster diameter obtained by analyzing PALM trajectories and clustering analysis according to SR-Tesseler under mock condition. Right panel quantification of the cluster diameter (nm).
(C-E) Left panels: representation of the frequency of SRF3^{WT}-mEOS2 (C) and SRF3^{5KR}-mEOS2 (D) or SRF3^{5WT}-mEOS2 under mock or treated conditions with ES9-17 at 30 µM (E) molecules according to their local density (log(local density)) obtained by analyzing tessellation-based automatic segmentation of super resolution images. Right panels: correspond to identical frequency analysis but processed by the R mClust package. "n" corresponds to the number of molecules analyzed.
(F) Micrographs of 5-day-old seedlings of plants co-expressing *pAP2µ::AP2µ-GFP* (AP2µ) or *pREM1.3::REM1.3-GFP* (REM1.3) with *pUBQ10::SRF3^{WT}-2xmCHERRY* (SRF3) or *pUBQ10::SRF3^{WT}-mCITRINE* with *pRPS5a::CLC2-RFP* (CLC2) and merged channels acquired by TIRF microscopy along with the related kymograph of CLC2 with SRF3 under cotreatment CHX 100 µM and BFA 25 µM. Arrows depict colocalization. Scale bars, 5 µm.
(G) Micrographs acquired by TIRF microscopy of 5-day-old seedlings expressing SRF3^{WT}-mCIT or SRF3^{5KR}-mCIT, under +Fe or -Fe supplemented with 100 µM of ferrozine (-FeFZ[100]), or flg22 at 1µM (flg22[1]) for 1 hour and the related quantification, right [two-way ANOVA, Tukey (HSD) test, letters indicate statistical differences ($p < 0.05$)]. Scale bars, 5 µm. In all graphs, the numbers below the box plot represent the number of individual measurements of one image on one single root.
(H) Micrographs acquired by TIRF microscopy of 5-day-old seedlings expressing SRF3^{WT}-mCIT, SRF3-mCIT^{Ub}, SRF3-mCIT$^{Ub\text{-}I44A}$ and the related quantification, below. [two-way Student's test ($p < 0.05$)]. Scale bars, 5 µm.
In all graphs the numbers shown below the box plots represent the count of individual measurements taken from a single image of one root, pooled across three independent experiments.

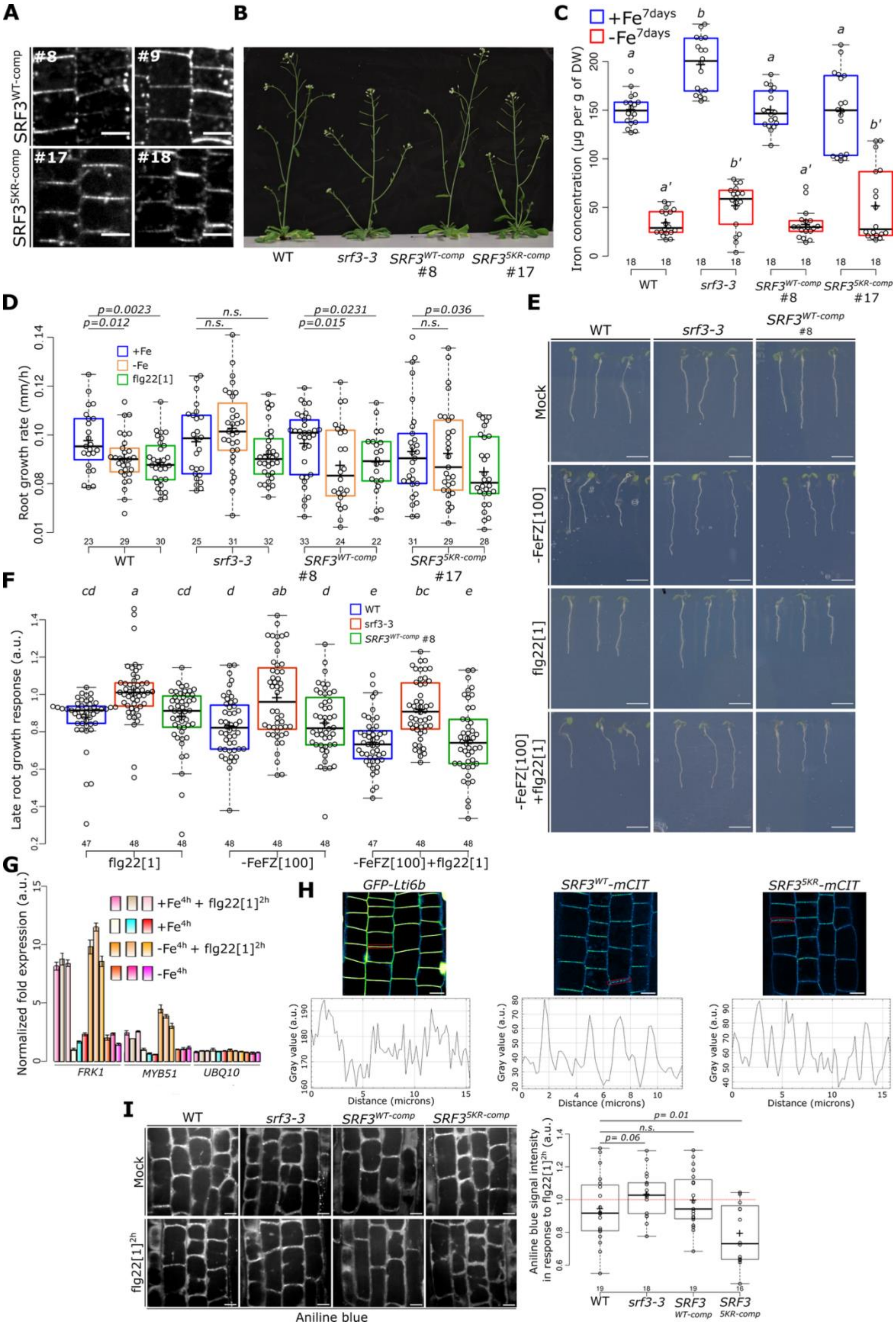
A
SRF3^WT-comp
SRF3^5KR-comp
#8
#9
#17
#18
B
WT
srf3-3
SRF3^WT-comp #8
SRF3^5KR-comp #17
C
+Fe^7days
-Fe^7days
Iron concentration (µg per g of DW)
WT
srf3-3
SRF3^WT-comp #8
SRF3^5KR-comp #17
D
Root growth rate (mm/h)
+Fe
-Fe
flg22[1]
p=0.0023
p=0.012
n.s.
p=0.0231
p=0.015
p=0.036
WT
srf3-3
SRF3^WT-comp #8
SRF3^5KR-comp #17
E
WT
srf3-3
SRF3^WT-comp #8
Mock
-FeFZ[100]
flg22[1]
-FeFZ[100] +flg22[1]
F
Late root growth response (a.u.)
WT
srf3-3
SRF3^WT-comp #8
flg22[1]
-FeFZ[100]
-FeFZ[100]+flg22[1]
G
Normalized fold expression (a.u.)
+Fe^4h + flg22[1]^2h
+Fe^4h
-Fe^4h + flg22[1]^2h
-Fe^4h
FRK1
MYB51
UBQ10
H
GFP-Lti6b
SRF3^WT-mCIT
SRF3^5KR-mCIT
Gray value (a.u.)
Distance (microns)
I
WT
srf3-3
SRF3^WT-comp
SRF3^5KR-comp
Mock
flg22[1]^2h
Aniline blue
Aniline blue signal intensity in response to flg22[1]^2h (a.u.)
p= 0.01
n.s.
p= 0.06
SRF3 WT-comp
SRF3 5KR-comp

Figure S7: SRF3 triggers different signaling pathways under flg22 and low ferrous iron depending on the ubiquitination of the five lysine residues.

(A) Confocal images of root epidermal cells of 5-day-old seedlings expressing pSRF3::SRF3^{WT}-mCITRINE/srf3-3 (SRF3$^{WT\text{-}comp}$) and pSRF3::SRF3^{5KR}-mCITRINE/srf3-3 (SRF3$^{5KR\text{-}comp}$). Numbers indicate the name of the independent transgenic insertion lines. Scale bars, 10 μM.

(B) Pictures of 45-day-old plants grown under controlled conditions of the plants WT, srf3-3, pSRF3::SRF3^{WT}-mCITRINE/srf3-3 (SRF3$^{WT\text{-}comp}$) and pSRF3::SRF3^{5KR}-mCITRINE/srf3-3 (SRF3$^{5KR\text{-}comp}$).

(C) Quantification of the total iron content of 12 days old seedlings of WT, pSRF3::SRF3^{WT}-mCITRINE/srf3-3 (SRF3$^{WT\text{-}comp}$) and pSRF3::SRF3^{5KR}-mCITRINE/srf3-3 (SRF3$^{5KR\text{-}comp}$) in mock condition or low iron (-Fe) for 7 days. [two-way ANOVA, Fisher (LSD) test, letters indicate statistical differences ($p < 0.05$)].

(D) Quantification of the root growth rate of WT, srf3-3, pSRF3::SRF3^{WT}-mCITRINE/srf3-3 (SRF3$^{WT\text{-}comp}$) and pSRF3::SRF3^{5KR}-mCITRINE/srf3-3 (SRF3$^{5KR\text{-}comp}$) in mock condition or low iron (-Fe) or flg22 at 1μM for 12 hours. Numbers indicate independent insertion lines. [Independent one-way Student's test ($p<0.05$)].

(E-F) Representative images of 8-day-old seedlings of WT, srf3-3, pSRF3::SRF3^{WT}-mCITRINE/srf3-3 (SRF3$^{WT\text{-}comp}$) grown under standard conditions for 5 days and then transferred for 3 days to mock conditions or to low iron medium supplemented with Ferrozine at 100μM (–FeFZ[100]) or flg22 at 1 μM or co-treated with low iron medium supplemented with Ferrozine at 100μM (-FeFZ[100]) and flg22 at 1 μM (E). Quantification of the late root growth response. Numbers indicate independent insertion lines (F). [two-way ANOVA followed by a post hoc Fisher LSD test, letters indicate statistical differences ($p < 0.05$)]. Scale bar, 1 cm.

(G) RT-qPCR indicating the normalized fold expression of FRK1, MYB51 and UBQ10 in the indicated conditions of 5-day-old seedlings on root material. Data include 3 technical repeats from 1 biological replicate represented by one bar. Experiment was repeated three times. SRF3 expression levels was normalized to the housekeeping gene TCTP. Error bars represent the standard deviation of the mean.

(H) Confocal images of root epidermal cells in the elongation zone of 5-day-old seedling expressing p35S::GFP-Lti6b (GFP-Lti6b), pUBQ10::SRF3^{WT}-mCITRINE (SRF3^{WT}-mCIT), pUBQ10::SRF3^{5KR}-mCITRINE (SRF3^{5KR}-mCIT) and the corresponding plot profile calculated on the plasma membrane depicted by the red rectangle. Scale bars, 10 μm.

(I) Confocal images of root epidermal cells in the elongation zone of 5-day-old seedling stained with aniline blue under mock and flg22 at 1 μM for 2 hours in WT, srf3-3, pSRF3::SRF3^{WT}-mCITRINE/srf3-3 (SRF3$^{WT\text{-}comp}$) and pSRF3::SRF3^{5KR}-mCITRINE/srf3-3 (SRF3$^{5KR\text{-}comp}$) and the related quantification [One way student's test ($p < 0.1$)]. Scale bars, 10 μm.

In all graphs the numbers shown below the box plots represent the count of individual measurements taken from a single image of one root, pooled across three independent experiments.

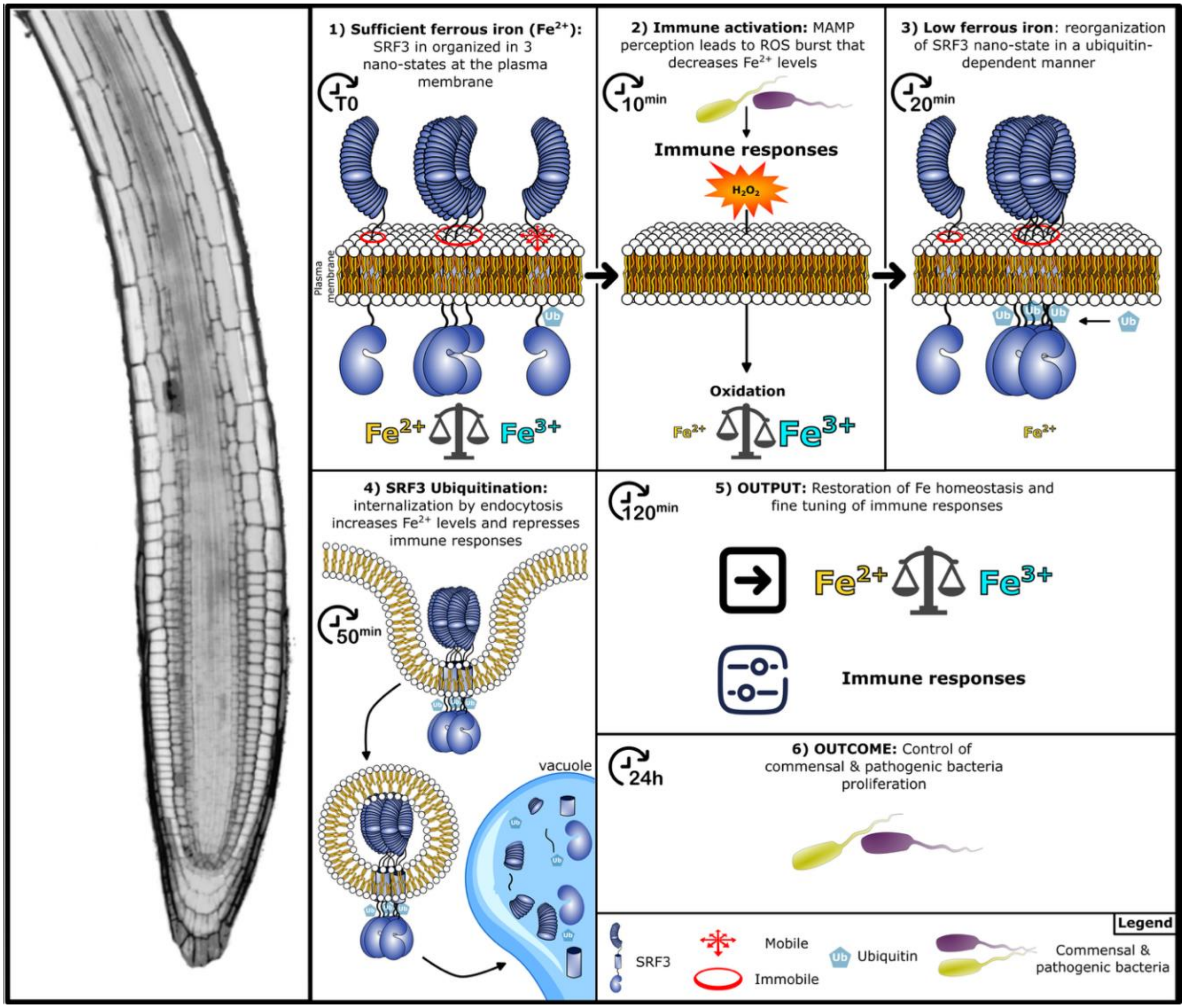

Figure S8: A model for SRF3 plasma membrane nano-organization in the regulation of iron homeostasis, immune responses, and bacterial proliferation.

Supplementary Tables

Table S1: Proteomics analysis of SRF3
Table S2: RNAseq data
Table S3: RNAseq clustering analysis
Table S4: RNAseq data of SRF3 overexpressing line

Table S5: List of pENTR clones

Name of entry vector	Source	Reference
SRF3prom_pDONR4P1R	Platre et al., 2022 (*17*)	N/A
pWER_pDONR4P1R	Marques et al., 2016 (*28*)	Set N2106366
pGL2_pDONR4P1R	Marques et al., 2016 (*28*)	Set N2106366
pPEP_pDONR4P1R	Marques et al., 2016 (*28*)	Set N2106366
pATHB8_pDONR4P1R	Marques et al., 2016 (*28*)	Set N2106366
pSCR_pDONR4P1R	Marques et al., 2016 (*28*)	Set N2106366
pPIN2_pDONR4P1R	Marques et al., 2016 (*28*)	Set N2106366
pCAB3_pDONR4P1R	Gaillochet et al., 2020 (*29*)	N/A
UBQ10prom_ pDONR4P1R	See NASC#	NASC# N2106315
$SRF3^{5KR}$_P1P2	This study	N/A
mCITRINE_ pDONR2RP3	See NASC#	NASC# N2106287
mCITRINE-Ub_ pDONR2RP3	Martins et al., 2015 (*30*)	N/A
mCITRINE-Ub-I44A_ pDONR2RP3	Martins et al., 2015 (*30*)	N/A
mCITRINE-Ub-7KR_ pDONR2RP3	Martins et al., 2015 (*30*)	N/A
mEOS2__ pDONR2RP3	Platre et al., 2019 (*36*)	N/A
2xmCHERRY-4xmyc_ pDONR2RP3	Platre et al., 2022 (*17*)	N/A

Construction of plant transformation vectors (destination vectors) and plant transformation and crosses:

Binary destination vectors for plant transformation were obtained using the multisite LR recombination system (life technologies, http://www.thermofisher.com/) using the pB7m34GW (basta resistant) or pH7m34GW (Hygromycin resistant)

Table S6: Plant transformation vectors (destination vectors)

Name of construct	pDONRP4 P1R	pDONR2 21	pDONRP2R P3	pDEST	Final construct
pSRF3::$SRF3^{5KR}$-mCITRINE	SRF3prom	$SRF3^{5KR}$cds	mCITRINE	pB7m34GW	pSRF3::$SRF3^{5KR}$-mCITRINE/pB7m34GW
pUBQ10::$SRF3^{5KR}$-mCITRINE	pUBQ10	$SRF3^{5KR}$cds	mCITRINE	pB7m34GW	pUBQ10::$SRF3^{5KR}$-mCITRINE/pB7m34GW

pUBQ10::SRF3^{WT}-2xmCHERRY-4xmyc	pUBQ10	SRF3^{WT}cds	2xmCHERRY-4xmyc	pH7m34GW	pUBQ10::SRF3-mCHERRY-4xmyc/pH7m34GW
pWER::SRF3-mCITRINE	pWER	SRF3^{WT}cds	mCITRINE	pB7m34GW	pWER::SRF3-mCITRINE/pB7m34GW
pGL2::SRF3-mCITRINE	pGL2	SRF3^{WT}cds	mCITRINE	pB7m34GW	pGL2::SRF3-mCITRINE/pB7m34GW
pPEP::SRF3-mCITRINE	pPEP	SRF3^{WT}cds	mCITRINE	pB7m34GW	pPEP::SRF3-mCITRINE/pB7m34GW
pATHB8::SRF3-mCITRINE	pATHB8	SRF3^{WT}cds	mCITRINE	pB7m34GW	pATHB8::SRF3-mCITRINE/pB7m34GW
pSCR::SRF3-mCITRINE	pSCR	SRF3^{WT}cds	mCITRINE	pB7m34GW	pSCR::SRF3-mCITRINE/pB7m34GW
pPIN2::SRF3-mCITRINE	pPIN2	SRF3^{WT}cds	mCITRINE	pB7m34GW	pPIN2::SRF3-mCITRINE/pB7m34GW
pCAB3::SRF3-mCITRINE	pCAB3	SRF3^{WT}cds	mCITRINE	pB7m34GW	pCAB3::SRF3-mCITRINE/pB7m34GW
pUBQ10::SRF3^{WT}-mEOS2	pUBQ10	SRF3^{WT}cds	mEOS2	pB7m34GW	pUBQ10::SRF3^{WT}-mEOS2/pB7m34GW
pUBQ10::SRF3^{5KR}-mEOS2	pUBQ10	SRF3^{5KR}cds	mEOS2	pB7m34GW	pUBQ10::SRF3^{5KR} -mEOS2/pB7m34GW
pUBQ10::SRF3-mCITRINE-^{Ub}	pUBQ10	SRF3^{WT}cds	mCITRINE-^{Ub}	pB7m34GW	pUBQ10::SRF3^{WT}-mCITRINE-^{Ub} /pB7m34GW
pUBQ10::SRF3-mCITRINE-$^{Ub\text{-}I44A}$	pUBQ10	SRF3^{WT}cds	mCITRINE-$^{Ub\text{-}I44A}$	pB7m34GW	pUBQ10::SRF3^{WT}-mCITRINE-$^{Ub\text{-}I44A}$ /pB7m34GW

Table S7: Lines made in this study

**Line**	**Background**	**Generation**
pSRF3::SRF3^{5KR}-mCITRINE/pB7m34GW	*srf3-3* in Col-0	Homozygous
pUBQ10::SRF3^{5KR}-mCITRINE/pB7m34GW	Col-0	Homozygous
pUBQ10::SRF3^{WT}-mCITRINE/pB7m34GW	*cpi1-1* in Col-0	Homozygous
pWER::SRF3-mCITRINE/pB7m34GW	Col-0	T2
pGL2::SRF3-mCITRINE/pB7m34GW	Col-0	T2
pPEP::SRF3-mCITRINE/pB7m34GW	Col-0	T2
pATHB8::SRF3-mCITRINE/pB7m34GW	Col-0	T2

pSCR::SRF3-mCITRINE/pB7m34GW	Col-0	T2
pPIN2::SRF3-mCITRINE/pB7m34GW	Col-0	T2
pCAB3::SRF3-mCITRINE/pB7m34GW	Col-0	T2
pUBQ10::SRF3-mCHERRY-4xmyc/pH7m34GW	Col-0	Homozygous
pUBQ10::SRF3-mCHERRY-4xmyc/pH7m34GW	*rbohD/F* in Col-0	Triple Homozygous
pUBQ10::SRF3^{WT}- mCITRINE-^{Ub} /pB7m34GW	Col-0	Homozygous
pUBQ10::SRF3^{WT}- mCITRINE-$^{Ub\text{-}I44A}$ /pB7m34GW	Col-0	Homozygous
pUBQ10::SRF3^{WT}- mCITRINE-$^{Ub\text{-}7KR}$ /pB7m34GW	Col-0	Homozygous
pUBQ10::SRF3^{WT}-mEOS2/pB7m34GW	Col-0	T2
pUBQ10::SRF3^{5KR} -mEOS2/pB7m34GW	Col-0	T2

Table S8: Crossing made in this study

**Line_1**	**Line_2**	**Generation**
2x35s::GFP-Lti6b	pUBQ10::SRF3-mCHERRY-4xmyc	F2
pCLC2::CLC2-GFP	pUBQ10::SRF3-mCHERRY-4xmyc	F2
pUBQ10::SRF3-mCITRINE	*pRPS5a::CLC2-RFP*	F2
pUBQ10::SRF3^{5KR}-mCITRINE	*pRPS5a::CLC2-RFP*	F2
pUBQ10::SRF3-mCITRINE	*pUBQ10::mCHERRY-VTI12*	F2
pUBQ10::SRF3-mCITRINE	*pUBQ10::mCHERRY-RabF2a*	F2
pUBQ10::SRF3-mCITRINE	*pUBQ10::mCHERRY-RabA5d*	F2
pUBQ10::SRF3^{5KR}-mCITRINE	*pUBQ10::mCHERRY-VTI12*	F2
pUBQ10::SRF3^{5KR}-mCITRINE	*pUBQ10::mCHERRY-RabF2a*	F2
pUBQ10::SRF3^{5KR}-mCITRINE	*pUBQ10::mCHERRY-RabA5d*	F2
pUBQ10::SRF3-mCITRINE	*chc1-1*	Double homozygous

pUBQ10::SRF3-mCITRINE	*chc2-3*	Double homozygous
XVE>>AX2	pUBQ10::SRF3-mCHERRY-4xmyc	Double homozygous
pAP2u::AP2u-GFP	pUBQ10::SRF3^{WT}-mCHERRY-4xmyc	F2
pREM1.3::REM1.3-GFP	pUBQ10::SRF3^{WT}-mCHERRY-4xmyc	F2

Table S9: Primer table

SRF3_cDNA_K391392R_p_F	agagctggagaggaaccaaaggtcttacatgaccttgagaggc
SRF3_cDNA_K391392R_p_R	cctgaagggttctctttgaactttttcagatcgaccaggtggtggc
SRF3c_K444R_R	ccgtgaccctctcatccaaaggaggaggtgg
SRF3c_K444R_F	ggatgagagggtcacggttatgccaatcatatc
SRF3_K458R_new_F	acccgttaggaaaacttcccctaaacgcttacccttaacttc
SRF3_K458R_new_R	cggttatgccaatcatatcacccgaaagacccgttaggaaaacttc
SRF3_K471R_F	cccttaacttctgtgaggcactattctattgcttctcttcaacaatacacgg
SRF3_K471R_R	gcaatagaatagtgcctcacagaagttaagggtaagcgtttaggggaag
RT_TCTP_F #2	cccgcaaccttcgattttcg
RT_TCTP_R #2	ggcaggtaattttaataggggacgacgac
RT_SRF3_F	gcatgctcggtagtgtttataggg
RT_SRF3_R	ctaaattctggttcagactgaacacac

REFERENCES

1. B. Galy, M. Conrad, M. Muckenthaler, Mechanisms controlling cellular and systemic iron homeostasis. *Nat. Rev. Mol. Cell Biol.* **25**, 133–155 (2024).

2. H. J. H. Fenton, LXXIII.—Oxidation of tartaric acid in presence of iron. *J. Chem. Soc. Trans.* **65**, 899–910 (1894).

3. F. Haber, J. Weiss, The catalytic decomposition of hydrogen peroxide by iron salts. *Proc. R. Soc. Lond. Ser. Math. Phys. Sci.* **147**, 332–351 (1934).

4. C. C. Murdoch, E. P. Skaar, Nutritional immunity: The battle for nutrient metals at the host–pathogen interface. *Nat. Rev. Microbiol.* **20**, 657–670 (2022).

5. R. L. Gonciarz, A. R. Renslo, Emerging role of ferrous iron in bacterial growth and host-pathogen interaction: New tools for chemical (micro)biology and antibacterial therapy. *Curr. Opin. Chem. Biol.* **61**, 170–178 (2021).

6. K. M. Mayle, A. M. Le, D. T. Kamei, The intracellular trafficking pathway of transferrin. *Biochim. Biophys. Acta* **1820**, 264–281 (2012).

7. H. Fujita, Y. Iwabu, K. Tokunaga, Y. Tanaka, Membrane-associated RING-CH (MARCH) 8 mediates the ubiquitination and lysosomal degradation of the transferrin receptor. *J. Cell Sci.* **126**, 2798–2809 (2013).

8. Y. Wu, H. Jiao, Y. Yue, K. He, Y. Jin, J. Zhang, J. Zhang, Y. Wei, H. Luo, Z. Hao, X. Zhao, Q. Xia, Q. Zhong, J. Zhang, Ubiquitin ligase E3 HUWE1/MULE targets transferrin receptor for degradation and suppresses ferroptosis in acute liver injury. *Cell Death Differ.* **29**, 1705–1718 (2022).

9. C. C. Murdoch, E. P. Skaar, Nutritional immunity: The battle for nutrient metals at the host–pathogen interface. *Nat. Rev. Microbiol.* **20**, 657–670 (2022).

10. J. H. Herlihy, T. A. Long, J. M. McDowell, Iron homeostasis and plant immune responses: Recent insights and translational implications. *J. Biol. Chem.* **295**, 13444–13457 (2020).

11. L. Bai, M. Qiao, R. Zheng, C. Deng, S. Mei, W. Chen, Phylogenomic analysis of transferrin family from animals and plants. *Comp. Biochem. Physiol. Part D Genomics Proteomics* **17**, 1–8 (2016).

12. G. Vert, N. Grotz, F. Dédaldéchamp, F. Gaymard, M. L. Guerinot, J.-F. Briat, C. Curie, IRT1, an Arabidopsis Transporter Essential for Iron Uptake from the Soil and for Plant Growth. *Plant Cell* **14**, 1223–1233 (2002).

13. T. A. Long, H. Tsukagoshi, W. Busch, B. Lahner, D. E. Salt, P. N. Benfey, The bHLH transcription factor POPEYE regulates response to iron deficiency in Arabidopsis roots. *Plant Cell* **22**, 2219–2236 (2010).

14. G. Dubeaux, J. Neveu, E. Zelazny, G. Vert, Metal sensing by the IRT1 transporter-receptor orchestrates its own degradation and plant metal nutrition. *Mol. Cell* **69**, 953–964.e5 (2018).

15. M. Cao, M. P. Platre, H.-H. Tsai, L. Zhang, T. Nobori, L. Armengot, Y. Chen, W. He, L. Brent, N. S. Coll, J. R. Ecker, N. Geldner, W. Busch, Spatial IMA1 regulation restricts root iron acquisition on MAMP perception. *Nature* **625**, 750–759 (2024).

16. T. Nozoye, S. Nagasaka, T. Kobayashi, M. Takahashi, Y. Sato, Y. Sato, N. Uozumi, H. Nakanishi, N. K. Nishizawa, Phytosiderophore efflux transporters are crucial for iron acquisition in graminaceous plants*. *J. Biol. Chem.* **286**, 5446–5454 (2011).

17. M. P. Platre, S. B. Satbhai, L. Brent, M. F. Gleason, M. Cao, M. Grison, M. Glavier, L. Zhang, C. Gaillochet, C. Goeschl, M. Giovannetti, B. Enugutti, J. Neveu, M. von Reth, R. Alcázar, J. E. Parker, G. Vert, E. Bayer, W. Busch, The receptor kinase SRF3 coordinates iron-level and flagellin dependent defense and growth responses in plants. *Nat. Commun.* **45**, 4445 (2022).

18. D. Chinchilla, C. Zipfel, S. Robatzek, B. Kemmerling, T. Nürnberger, J. D. G. Jones, G. Felix, T. Boller, A flagellin-induced complex of the receptor FLS2 and BAK1 initiates plant defence. *Nature* **448**, 497–500 (2007).

19. M. A. Torres, J. L. Dangl, J. D. G. Jones, Arabidopsis gp91phox homologues AtrbohD and AtrbohF are required for accumulation of reactive oxygen intermediates in the plant defense response. *Proc. Natl. Acad. Sci. U.S.A.* **99**, 517–522 (2002).

20. J. P. Ferguson, N. M. Willy, S. P. Heidotting, S. D. Huber, M. J. Webber, C. Kural, Deciphering dynamics of clathrin-mediated endocytosis in a living organism. *J. Cell Biol.* **214**, 347–358 (2016).

21. M. Adamowski, M. Narasimhan, U. Kania, M. Glanc, G. De Jaeger, J. Friml, A functional study of AUXILIN-LIKE1 and 2, two putative clathrin uncoating factors in arabidopsis. *Plant Cell* **30**, 700–716 (2018).

22. W. Dejonghe, I. Sharma, B. Denoo, S. De Munck, Q. Lu, K. Mishev, H. Bulut, E. Mylle, R. De Rycke, M. Vasileva, D. V. Savatin, W. Nerinckx, A. Staes, A. Drozdzecki, D. Audenaert, K. Yperman, A. Madder, J. Friml, D. Van Damme, K. Gevaert, V. Haucke, S. N. Savvides, J. Winne, E. Russinova, Disruption of endocytosis through chemical inhibition of clathrin heavy chain function. *Nat. Chem. Biol.* **15**, 641–649 (2019).

23. S. Mayor, R. G. Parton, J. G. Donaldson, Clathrin-independent pathways of endocytosis. *Cold Spring Harb. Perspect. Biol.* **6**, a016758 (2014).

24. R. Li, P. Liu, Y. Wan, T. Chen, Q. Wang, U. Mettbach, F. Baluška, J. Šamaj, X. Fang, W. J. Lucas, J. Lin, A membrane microdomain-associated protein, *Arabidopsis* Flot1, is involved in a clathrin-independent endocytic pathway and is required for seedling development. *Plant Cell* **24**, 2105–2122 (2012).

25. A. Ciechanover, The unravelling of the ubiquitin system. *Nat. Rev. Mol. Cell Biol.* **16**, 322–324 (2015).

26. J. Gao, A. Chaudhary, P. Vaddepalli, M.-K. Nagel, E. Isono, K. Schneitz, The Arabidopsis receptor kinase STRUBBELIG undergoes clathrin-dependent endocytosis. *J. Exp. Bot.* **70**, 3881–3894 (2019).

27. L. E. Grubb, P. Derbyshire, K. E. Dunning, C. Zipfel, F. L. H. Menke, J. Monaghan, Large-scale identification of ubiquitination sites on membrane-associated proteins in Arabidopsis thaliana seedlings. *Plant Physiol.* **185**, 1483–1488 (2021).

28. M. D. M. Marquès-Bueno, A. K. Morao, A. Cayrel, M. P. Platre, M. Barberon, E. Caillieux, V. Colot, Y. Jaillais, F. Roudier, G. Vert, A versatile Multisite Gateway-compatible promoter and transgenic line collection for cell type-specific functional genomics in Arabidopsis. *Plant J. Cell Mol. Biol.* **85**, 320–333 (2016).

29. C. Gaillochet, Y. Burko, M. P. Platre, L. Zhang, J. Simura, B. Willige, S. V. Kumar, K. Ljung, J. Chory, W. Busch, HY5 and phytochrome activity modulate shoot-to-root coordination during thermomorphogenesis in Arabidopsis. *Development* **147**, dev192625 (2020).

30. S. Martins, E. M. N. Dohmann, A. Cayrel, A. Johnson, W. Fischer, F. Pojer, B. Satiat-Jeunemaître, Y. Jaillais, J. Chory, N. Geldner, G. Vert, Internalization and vacuolar targeting of the brassinosteroid hormone receptor BRI1 are regulated by ubiquitination. *Nat. Commun.* **6**, 6151 (2015).

31. P. Grones, A. De Meyer, R. Pleskot, E. Mylle, M. Kraus, M. Vandorpe, K. Yperman, D. Eeckhout, J. M. Dragwidge, Q. Jiang, J. Nolf, B. Pavie, G. De Jaeger, B. De Rybel, D. Van Damme, The endocytic TPLATE complex internalizes ubiquitinated plasma membrane cargo. *Nat. Plants* **8**, 1467–1483 (2022).

32. K. N. Swatek, D. Komander, Ubiquitin modifications. *Cell Res.* **26**, 399–422 (2016).

33. R. S. Gormal, R. Martinez-Marmol, A. J. Brooks, F. A. Meunier, Location, location, location: Protein kinase nanoclustering for optimised signalling output. *eLife* **13**, e93902 (2024).

34. S. Kim, K. K. Okafor, R. Tabuchi, C. Briones, I.-H. Lee, Phase separation clustering of poly ubiquitin cargos on ternary mixture lipid membranes by synthetically cross-linked ubiquitin binder peptides. *Biochemistry* **64**, 1212–1221 (2025).

35. V. Bayle, J.-B. Fiche, C. Burny, M. P. Platre, M. Nollmann, A. Martinière, Y. Jaillais, Single-particle tracking photoactivated localization microscopy of membrane proteins in living plant tissues. *Nat. Protoc.* **16**, 1600–1628 (2021).

36. M. P. Platre, V. Bayle, L. Armengot, J. Bareille, M. del M. Marquès-Bueno, A. Creff, L. Maneta-Peyret, J.-B. Fiche, M. Nollmann, C. Miège, P. Moreau, A. Martinière, Y. Jaillais, Developmental control of plant Rho GTPase nano-organization by the lipid phosphatidylserine. *Science* **364**, 57–62 (2019).

37. L. P. Sousa, I. Lax, H. Shen, S. M. Ferguson, P. D. Camilli, J. Schlessinger, Suppression of EGFR endocytosis by dynamin depletion reveals that EGFR signaling occurs primarily at the plasma membrane. *Proc. Natl. Acad. Sci. U.S.A.* **109**, 4419–4424 (2012).

38. F. Levet, E. Hosy, A. Kechkar, C. Butler, A. Beghin, D. Choquet, J.-B. Sibarita, SR-Tesseler: A method to segment and quantify localization-based super-resolution microscopy data. *Nat. Methods* **12**, 1065–1071 (2015).

39. B. Xu, C. Cheval, A. Laohavisit, B. Hocking, D. Chiasson, T. S. G. Olsson, K. Shirasu, C. Faulkner, M. Gilliham, A calmodulin-like protein regulates plasmodesmal closure during bacterial immune responses. *New Phytol.* **215**, 77–84 (2017).

40. Y. A. Millet, C. H. Danna, N. K. Clay, W. Songnuan, M. D. Simon, D. Werck-Reichhart, F. M. Ausubel, Innate immune responses activated in arabidopsis roots by microbe-associated molecular patterns. *Plant Cell* **22**, 973–990 (2010).

41. Z. Zhong, S. Zhou, Y. Liang, Y. Wei, Y. Li, T. Long, Q. He, M. Li, Y. Zhou, Y. Yu, L. Fang, X. Liao, B. N. Kreiswirth, L. Chen, H. Ren, Y. Liu, J. Sun, Natural flavonoids disrupt bacterial iron homeostasis to potentiate colistin efficacy. *Sci. Adv.* **9**, eadg4205 (2023).

42. S. Pfeilmeier, I. M.-L. Saur, J. P. Rathjen, C. Zipfel, J. G. Malone, High levels of cyclic-di-GMP in plant-associated Pseudomonas correlate with evasion of plant immunity. *Mol. Plant Pathol.* **17**, 521–531 (2016).

43. C. Pfund, J. Tans-Kersten, F. M. Dunning, J. M. Alonso, J. R. Ecker, C. Allen, A. F. Bent, Flagellin is not a major defense elicitor in Ralstonia solanacearum cells or extracts applied to Arabidopsis thaliana. *Mol. Plant Microbe Interactions.* **17**, 696–706 (2004).

44. L. Li, M. Li, L. Yu, Z. Zhou, X. Liang, Z. Liu, G. Cai, L. Gao, X. Zhang, Y. Wang, S. Chen, J.-M. Zhou, The FLS2-associated kinase BIK1 directly phosphorylates the NADPH oxidase RbohD to control plant immunity. *Cell Host Microbe* **15**, 329–338 (2014).

45. G. Liu, D. L. Greenshields, R. Sammynaiken, R. N. Hirji, G. Selvaraj, Y. Wei, Targeted alterations in iron homeostasis underlie plant defense responses. *J. Cell Sci.* **120**, 596–605 (2007).

46. D. Israel, S. H. Lee, T. M. Robson, J. J. Zwiazek, Plasma membrane aquaporins of the PIP1 and PIP2 subfamilies facilitate hydrogen peroxide diffusion into plant roots. *BMC Plant Biol.* **22**, 566 (2022).

47. S. J. Dixon, B. R. Stockwell, The role of iron and reactive oxygen species in cell death. *Nat. Chem. Biol.* **10**, 9–17 (2014).

48. Y. Duan, Z. Wang, Y. Fang, Z. Pei, H. Hu, Q. Xu, H. Liu, X. Chen, C. Luo, J. Huang, L. Zheng, X. Chen, A secreted fungal laccase targets the receptor kinase OsSRF3 to inhibit OsBAK1–OsSRF3-mediated immunity in rice. *Nat. Commun.* **15**, 7891 (2024).

49. H. Wang, Y. Chen, X. Wu, Z. Long, C. Sun, H. Wang, S. Wang, P. R. J. Birch, Z. Tian, A potato STRUBBELIG-RECEPTOR FAMILY member, StLRPK1, associates with StSERK3A/BAK1 and activates immunity. *J. Exp. Bot.* **69**, 5573–5586 (2018).

50. W. Shi, M. Bilstein-Schloemer, S. C. Stolze, M. P. Platre, M. Hofmann, G. Doehlemann, H. Nakagami, I. M. L. Saur, Targeting of the barley cell-surface receptor SRF3 by the Blumeria hordei effector AVRA13 overlaps with AVRA13 recognition by MLA and the induction of NLR-mediated cell death. bioRxiv 2026.01.08.698370 [Preprint] (2026). https://doi.org/10.64898/2026.01.08.698370.

51. J. M. Dragwidge, Y. Wang, L. Brocard, A. De Meyer, R. Hudeček, D. Eeckhout, P. Grones, M. Buridan, C. Chambaud, P. Pejchar, M. Potocký, J. Winkler, M. Vandorpe, N. Serre, M. Fendrych, A. Bernard, G. De Jaeger, R. Pleskot, X. Fang, D. Van Damme, Biomolecular condensation orchestrates clathrin-mediated endocytosis in plants. *Nat. Cell Biol.* **26**, 438–449 (2024).

52. Y. Cui, H. Qian, J. Yin, C. Xu, P. Luo, X. Zhang, M. Yu, B. Su, X. Li, J. Lin, Single-molecule analysis reveals the phosphorylation of FLS2 governs its spatiotemporal dynamics and immunity. *eLife* **12**, RP91072 (2024).

53. Y. Wang, J. Gao, X. Guo, T. Tong, X. Shi, L. Li, M. Qi, Y. Wang, M. Cai, J. Jiang, C. Xu, H. Ji, H. Wang, Regulation of EGFR nanocluster formation by ionic protein-lipid interaction. *Cell Res.* **24**, 959–976 (2014).

54. S. Sigismund, T. Woelk, C. Puri, E. Maspero, C. Tacchetti, P. Transidico, P. P. D. Fiore, S. Polo, Clathrin-independent endocytosis of ubiquitinated cargos. *Proc. Natl. Acad. Sci. U.S.A.* **102**, 2760–2765 (2005).

55. M. N. Hindt, G. Z. Akmakjian, K. L. Pivarski, T. Punshon, I. Baxter, D. E. Salt, M. L. Guerinot, BRUTUS and its paralogs, BTS LIKE1 and BTS LIKE2, encode important negative regulators of the iron deficiency response in Arabidopsis thaliana. *Metallomics* **9**, 876–890 (2017).

56. Y. Fichman, R. J. Myers, D. G. Grant, R. Mittler, Plasmodesmata-localized proteins and ROS orchestrate light-induced rapid systemic signaling in *Arabidopsis*. *Sci. Signal.* **14**, eabf0322 (2021).

57. P. J. P. L. Teixeira, N. R. Colaianni, T. F. Law, J. M. Conway, S. Gilbert, H. Li, I. Salas-González, D. Panda, N. M. Del Risco, O. M. Finkel, G. Castrillo, P. Mieczkowski, C. D. Jones, J. L. Dangl, Specific modulation of the root immune system by a community of commensal bacteria. *Proc. Natl. Acad. Sci. U.S.A.* **118**, e2100678118 (2021).

58. R. Alcázar, A. V. García, I. Kronholm, J. de Meaux, M. Koornneef, J. E. Parker, M. Reymond, Natural variation at Strubbelig Receptor Kinase 3 drives immune-triggered incompatibilities between Arabidopsis thaliana accessions. *Nat. Genet.* **42**, 1135–1139 (2010).

59. E. R. Larson, E. V. Zelm, C. Roux, A. Marion-Poll, M. R. Blatt, Clathrin heavy chain subunits coordinate endo- and exocytic traffic and affect stomatal movement. *Plant Physiol.* **175**, 708–720 (2017).

60. C. A. Konopka, S. K. Backues, S. Y. Bednarek, Dynamics of arabidopsis dynamin-related protein 1C and a clathrin light chain at the plasma membrane. *Plant Cell* **20**, 1363–1380 (2008).

61. N. Geldner, V. Dénervaud-Tendon, D. L. Hyman, U. Mayer, Y.-D. Stierhof, J. Chory, Rapid, combinatorial analysis of membrane compartments in intact plants with a multicolor marker set. *Plant J. Cell Mol. Biol.* **59**, 169–178 (2009).

62. S. R. Cutler, D. W. Ehrhardt, J. S. Griffitts, C. R. Somerville, Random GFP::cDNA fusions enable visualization of subcellular structures in cells of Arabidopsis at a high frequency. *Proc. Natl. Acad. Sci. U.S.A.* **97**, 3718–3723 (2000).

63. J. Dettmer, A. Hong-Hermesdorf, Y.D. Stierhof, K. Schumacher, Vacuolar H+-ATPase Activity Is Required for Endocytic and Secretory Trafficking in Arabidopsis. *Plant Cell* **18**, 715–730 (2006).

64. B. D. Gruber, R. F. H. Giehl, S. Friedel, N. von Wirén, Plasticity of the arabidopsis root system under nutrient deficiencies. *Plant Physiol.* **163**, 161–179 (2013).

65. M. P. Platre, P. Mehta, Z. Halvorson, L. Zhang, L. Brent, M. Gleason F, K. Faizi, C. Goulding, W. Busch, Root Walker: An automated pipeline for large scale quantification of early root growth responses at high spatial and temporal resolution. *Plant J.* **117**, 632–646 (2024).

66. A. Martinière, I. Lavagi, G. Nageswaran, D. J. Rolfe, L. Maneta-Peyret, D.-T. Luu, S. W. Botchway, S. E. D. Webb, S. Mongrand, C. Maurel, M. L. Martin-Fernandez, J. Kleine-Vehn, J. Friml, P. Moreau, J. Runions, Cell wall constrains lateral diffusion of plant plasma-membrane proteins. *Proc. Natl. Acad. Sci. U.S.A.* **109**, 12805–12810 (2012).

67. M. Cao, R. Chen, P. Li, Y. Yu, R. Zheng, D. Ge, W. Zheng, X. Wang, Y. Gu, Z. Gelová, J. Friml, H. Zhang, R. Liu, J. He, T. Xu, TMK1-mediated auxin signalling regulates differential growth of the apical hook. *Nature* **568**, 240–243 (2019).

68. C. K. Gautam, H.-H. Tsai, W. Schmidt, A quick method to quantify iron in arabidopsis seedlings. *Bio Protoc.* **12**, e4342 (2022).

69. D. Kim, G. Pertea, C. Trapnell, H. Pimentel, R. Kelley, S. L. Salzberg, TopHat2: Accurate alignment of transcriptomes in the presence of insertions, deletions and gene fusions. *Genome Biol.* **14**, R36 (2013).

70. S. Anders, P. T. Pyl, W. Huber, HTSeq—a Python framework to work with high-throughput sequencing data. *Bioinformatics* **31**, 166–169 (2015).

71. M. D. Robinson, D. J. McCarthy, G. K. Smyth, edgeR: A Bioconductor package for differential expression analysis of digital gene expression data. *Bioinformatics* **26**, 139–140 (2010).

72. F. Zhou, A. Emonet, V. Dénervaud Tendon, P. Marhavy, D. Wu, T. Lahaye, N. Geldner, Co-incidence of damage and microbial patterns controls localized immune responses in roots. *Cell* **180**, 440–453.e18 (2020).

73. C. J. Harbort, M. Hashimoto, H. Inoue, Y. Niu, R. Guan, A. D. Rombolà, S. Kopriva, M. J. E. E. E. Voges, E. S. Sattely, R. Garrido-Oter, P. Schulze-Lefert, Root-secreted coumarins and the microbiota interact to improve iron nutrition in arabidopsis. *Cell Host Microbe* **28**, 825–837.e6 (2020).

74. K. Hiruma, N. Gerlach, S. Sacristán, R. T. Nakano, S. Hacquard, B. Kracher, U. Neumann, D. Ramírez, M. Bucher, R. J. O'Connell, P. Schulze-Lefert, Root endophyte colletotrichum tofieldiae confers plant fitness benefits that are phosphate status dependent. *Cell* **165**, 464–474 (2016).

75. A. P. Z. Cruz, V. Ferreira, M. J. Pianzzola, M. I. Siri, N. S. Coll, M. Valls, A Novel, Sensitive method to evaluate potato germplasm for bacterial wilt resistance using a Luminescent Ralstonia solanacearum reporter strain. *Mol. Plant Microbe Interact.* **27**, 277–285 (2014).

76. F. Monteiro, M. Solé, I. van Dijk, M. Valls, A chromosomal insertion toolbox for promoter probing, mutant complementation, and pathogenicity studies in ralstonia solanacearum. *Mol. Plant Microbe Interact.* **25**, 557–568 (2012).
